# FP-ADMET: a compendium of fingerprint-based ADMET prediction models

**DOI:** 10.1186/s13321-021-00557-5

**Published:** 2021-09-28

**Authors:** Vishwesh Venkatraman

**Affiliations:** Norwegian University of Science and Technology, Realfagbygget, Gløshaugen, Høgskoleringen, 7491 Trondheim, Norway

**Keywords:** ADMET, Machine learning, Molecular fingerprints

## Abstract

**Motivation:**

The absorption, distribution, metabolism, excretion, and toxicity (ADMET) of drugs plays a key role in determining which among the potential candidates are to be prioritized. In silico approaches based on machine learning methods are becoming increasing popular, but are nonetheless limited by the availability of data. With a view to making both data and models available to the scientific community, we have developed FPADMET which is a repository of molecular fingerprint-based predictive models for ADMET properties.

**Summary:**

In this article, we have examined the efficacy of fingerprint-based machine learning models for a large number of ADMET-related properties. The predictive ability of a set of 20 different binary fingerprints (based on substructure keys, atom pairs, local path environments, as well as custom fingerprints such as all-shortest paths) for over 50 ADMET and ADMET-related endpoints have been evaluated as part of the study. We find that for a majority of the properties, fingerprint-based random forest models yield comparable or better performance compared with traditional 2D/3D molecular descriptors.

**Availability:**

The models are made available as part of open access software that can be downloaded from https://gitlab.com/vishsoft/fpadmet.

**Supplementary Information:**

The online version contains supplementary material available at 10.1186/s13321-021-00557-5.

## Introduction

Properties such as absorption, distribution, metabolism, excretion and toxicity (ADMET), are an important component of pharmaceutical drug design. It is often reported that the failure to meet requisite ADMET criteria are a common cause for the high attrition rates of drug candidates [1]. Early ADMET profiling is indeed desirable so as to mitigate the risk of attrition. Various medium and high-throughput in vitro ADMET screens have therefore been developed, that have contributed to the available experimental data. These are nonetheless quite expensive especially when thousands of compounds are involved. Furthermore, reducing animal testing has now become a priority.

With the aim of facilitating rapid and inexpensive means of ADMET profiling, various in silico tools have been developed [2]. Using databases of experimentally measured ADMET properties [3], various quantitative structure-activity/property relationship (QSAR/QSPR) models have been generated that can predict a range of ADMET properties for novel chemical entities. Other efforts have made use of ADMET predictions to evaluate drug-likeness of a compound [4, 5]. While some of the models are available as part of commercial software packages based on proprietary datasets, there has been a significant push for open source software and web services [6–12].

Among the popular services, ADMETLab [12] offers 53 prediction models that are calculated using a multi-task graph attention network and operates on graph-structured data. The method is able to generate customized fingerprints from the general features for a specific task. Another web tool, SwissADME [9] evaluates pharmacokinetics, drug-likeness of small molecules. The predictions are based on a combination of fragmental methods (for solubility), as well as machine-learning based binary classification methods for other ADMET properties (cytochrome-P450 inhibitor, P-glycoprotein substrate). In ADMETSar [11], models for applications in both drug discovery and environmental risk assessment are built using MACCS and Morgan fingerprints. The toxicity models used in ProTox [13] are developed based on chemical similarities between compounds with known toxic effects and the presence of toxic fragments. Other models for hepatotoxicity, cytotoxicity, mutagenicity, and carcinogenicity rely on fingerprints (MACCS/Morgan). Extended connectivity fingerprints form the basis for the prediction of 15 ADMET properties in the vNN server [10] where models are trained using variable nearest neighbourhood method. pkCSM [6], on the other hand, uses graph-based signatures to develop predictive models of central ADMET properties. Other software such as MDCKPred [14], CarcinoPred-EL [15], CapsCarcino [16] focus on a single property such as the prediction of permeability coefficient and carcinogenic compounds. Overall, the molecular representations underlying these models include various molecular and physicochemical descriptors such as fingerprints, graph signatures, and other 2D/3D indices [17, 18]. Among these, fingerprint representations which are seen as an alternative to descriptors for QSPR studies, have been quite popular given their ease of computation and predictive value.

A number of fingerprints ranging from substructure/path to feature-class/circular have been proposed many of which are used in similarity searching [19, 20]. For ADMET studies however, the fingerprints studied so far have largely been restricted to a select few. In this study, we have evaluated the predictive efficacy of 20 different fingerprints ranging from substructure and extended/functional connectivity fingerprints to various path based encodings (depth-first search, shortest path, local path environments) [21]. The fingerprint-based regression/classification models were calculated for over 50 ADMET and ADMET-related endpoints (using data collated from various literature sources) and is to our knowledge one of the most comprehensive compilations analysed. For a majority of the endpoints, the prediction results were found to be comparable with more sophisticated descriptor formulations. Although the pharmacophore fingerprints yielded consistently poor results, others such as the PUBCHEM, MACCS and ECFP/FCFP encodings were found to yield the best results for most properties. The models and related software have been bundled into a downloadable package and is released under the GNU license.

## Approach

### Molecular representation

In this study, we have examined 20 different fingerprints (see Table 1) that are routinely used as similarity search tools in drug discovery. The ECFP- and FCFP-class fingerprints are circular topological fingerprints, where the former focuses on the atom properties (e.g. atomic number, charge, hydrogen count), whereas in the functional connectivity FPs, the emphasis is on properties that relate to ligand binding (e.g. hydrogen donor/acceptor, polarity, aromaticity). MACCS and PUBCHEM fingerprints are substructure fingerprints that cover a wide range of features such as element counts and ring systems, atom pairing, or atom environment etc. Other fingerprints include path based fingerprints such as the depth-first search fingerprints (DFS), all-shortest path encoding (ASP), radial fingerprints (Molprint2D), topological atom pairs (AP2D) and triplets (AT2D), pharmacophore pair and triplet encodings as well as local path environments [21]. Fingerprint calculations were performed using in-house code written in Java and makes use of the Chemistry Development Kit library [22]. The software merges existing fingerprints in the library with those calculated by the software *jCompoundMapper* [21].

**Table 1:** Fingerprints used in this study to model different ADMET related properties

Fingerprint	Size
MACCS	166
PUBCHEM	881
Klekota-Roth (KR)	4860
MOLPRINT (RAD2D)	4096
Atom pair (AP), atom triplet (AT)	4096
Local path environments (LSTAR)	4096
All-shortest path (ASP)	4096
Depth first search (DFS)	4096
Extended conectivity (ECFP: 0, 2, 4, 6)	1024
Functional class (FCFP: 0, 2, 4, 6)	1024
Pharmacophore: 2PPHAR/3PPHAR (2/3 point)	4096
ESTATE	79

Descriptions and implementation details of the different fingerprints are provided in the article by Hinselmann et al [21] and the references therein

### Data curation

Data for different endpoints were collected from previously published articles and databases with a primary source being the Online Chemical Database (OCHEM) [3]. The molecules were subsequently cleaned and duplicates (where present) were removed. Tables 2 and 3 lists the various endpoints and associated data sources considered in this study. Brief descriptions of the endpoints and the results from previous modelling efforts are provided in Additional file 1. Since, early identification of severe toxicity is a key requirement for the safety evaluation of drug candidates, we have evaluated a number of toxicity models covering a range of endpoints such as cardiac, hepatotoxicity, endocrine, urinary tract, carcinogenicity and cytotoxicity. While a majority of the models are binary classification models, for some endpoints such the metabolic intrinsic clearance, acute oral toxicity in rats, plasma protein binding and elimination half-life, multiclass models are proposed.

**Table 2:** Summary of the ADMET endpoints studied

Endpoint	Model	#Compounds	Group	Data source
Blood brain barrier	BC	7236	Distribution	[3, 31]
Oral bioavailability	BC	1822	Absorption	[3, 32]
Anticommensal effect	BC	1181	Toxicity	[33, 34]
CYP450 (1A2) inhibition	BC	17119	Metabolism	[35]
CYP450 (2C19) inhibition	BC	17119	Metabolism	[35]
CYP450 (2C9) inhibition	BC	17119	Metabolism	[35]
CYP450 (2D6) inhibition	BC	17119	Metabolism	[35]
CYP450 (3A4) inhibition	BC	17119	Metabolism	[35]
CYP450 (2C8) inhibition	BC	533	Metabolism	[36]
HIA	BC	1516	Absorption	[3, 37]
BCRP inhibition	BC	2799	Metabolism	[38]
Metabolic intrinsic clearance	MC	5278	Excretion	[39]
Human liver microsomal stability	BC	3654		[40]
PGP inhibitor	BC	2930	Distribution	[3, 41]
PGP substrate	BC	2198	Distribution	[3, 41]
DMSO solubility	BC	59047		[42]
Phosphate buffer solubility	BC	57584		[43]
Skin sensitization (LLNA)	BC	1033	Toxicity	[44]
Skin sensitization (KeratinSens)	BC	190	Toxicity	[44]
Skin sensitization (HRIPT)	BC	138	Toxicity	[44]
Skin sensitization (h-CLAT)	BC	160	Toxicity	[44]
Skin sensitization (DPRA)	BC	194	Toxicity	[44]
Rat acute oral toxicity ($\text {LD}_{{50}}$)	MC	11363	Toxicity	[3, 45]
AMES mutagenecity	BC	7950	Toxicity	[46]
Cytotoxicity (HepG2)	BC	6081	Toxicity	[10]
Cytotoxicity (CRL-7250 cell line)	BC	5241	Toxicity	[47]
Cytotoxicity (HACAT cell line)	BC	5241	Toxicity	[47]
Cytotoxicity (HEK cell line)	BC	5241	Toxicity	[47]
Cytotoxicity (NIK cell line)	BC	5241	Toxicity	[47]
DILI	BC	2478	Toxicity	[48]
Hemolytic toxicity (saponins)	BC	452	Toxicity	[49]
hERG cardiotoxicity	BC	7889	Toxicity	[50]
hERG liability	BC	9204		[51]
Mitochondrial toxicity	BC	6467	Toxicity	[52]
Urinary tract toxicity	BC	213	Toxicity	[53, 54]
Phototoxicity	BC	516	Toxicity	[55]
Phototoxicity	BC	1419	Toxicity	[55]
Toxic myopathy	BC	232	Toxicity	[56]
Myelotoxicity	BC	907	Toxicity	[57]
Phospholipidosis	BC	1719	Toxicity	[58]
Choleostasis	BC	1926	Toxicity	[59]
Rhabdomyolysis	BC	1504	Toxicity	[60]
Respiratory toxicity	BC	1241	Toxicity	[61]
Ototoxicity	BC	2612	Toxicity	[62]
MATE1 inhibition	BC	853	Metabolism	[63]
Hepatic steatosis	BC	512	Toxicity	[64]
Carcinogenecity	BC	1003	Toxicity	[15]
OATP1B1 inhibition	BC	1339	Metabolism	[65]
OATP2B1 inhibition	BC	230	Metabolism	[65]
OATP1B3 inhibition	BC	1249	Metabolism	[65]
BSEP inhibition	BC	1634	Metabolism	[66]
OCT2 inhibition	BC	907	Metabolism	[67]
PPB	MC	8103	Distribution	[3, 68]
Elimination half-life *Human*	MC	2127	Excretion	[69]
Elimination half-life *Mouse*	MC	808	Excretion	[69]
Elimination half-life *Rat*	MC	1308	Excretion	[69]

Here BC and MC refer to binary and multiclass classification respectively

*OATP* organic anion transporting polypeptide, *CYP-450* cytochrome-P450, *BCRP* breast cancer resistance protein, *BSEP* bile salt export pump, *DILI* drug-induced liver injury, *OCT* organic cation transporter 2, *MATE1* multidrug toxin extrusion transporter, *hERG* human Ether-á-go-go-related gene, *HIA* human intestinal absorption, *PPB* plasma protein binding, *PGP* p-glycoprotein, *LLNA* local lymph node assay, *DPRA* direct peptide reactivity assay, *h-CLAT* human cell line activation, *HRIPT* human repeat insult patch test, *HEK 293* human embryonic kidney 293 cell, *MATE1* multidrug and toxin extrusion transporter 1

**Table 3:** Summary of the ADMET and other endpoints for which fingerprint-based regression models were evaluated

Endpoint	#Compounds	Group	Data source
Aqueous solubility ($\log$S)	9982		[70]
Intrinsic clearance ($CL_{int}$)	244	Excretion	[71]
Skin penetration ($\log \ k_p$)	211	Toxicity	[72]
Human serum albumin	198		[73, 74]
Human placenta barrier (clearance index)	88	Distribution	[75]
Cancer potency in mouse ($\text {TD}_{{50}}$)	402	Toxicity	[76]
Cancer potency in rat ($\text {TD}_{{50}}$)	511	Toxicity	[76]
Steady state volume distribution ($\text {VD}_{{ss}}$)	1951	Distribution	[3, 77]
Distribution coefficient ($\log$ D)	7321		[3, 78]
Fraction unbound in human plasma	2319	Distribution	[79]
Fraction unbound in the brain	253	Distribution	[80]
Human liver microsomal clearance	5348	Excretion	[30]
Rat liver microsomal clearance	2166	Excretion	[30]
Mouse liver microsomal clearance	790	Excretion	[30]
CACO-2 permeability	2578	Absorption	[30]
$\text {pK}_a$	11041		[81, 82]
MDCK cell line permeability	701	Absorption	[3]
Human renal clearance (${CL}_r$)	636	Excretion	[83]
Hemolytic toxicity ($\log HD_{50}$)	875	Toxicity	[84]

*MDCK* Madin-Darby canine kidney

For other endpoints, regression models have been evaluated (see Table 3). These include the CACO-2 permeability which is commonly used to predict the absorption of orally administered drugs and other xenobiotics, the fraction of unbound drug in plasma, the liver microsomal clearance (typically used to predict hepatic clearance in humans), in vitro human skin permeability and the cancer potency. Models for other ADMET-related properties have also been studied. For instance, properties such as the dissociation constant ($\text {pK}_a$) affect solubility ($\log$ S), permeability, distribution coefficient ($\log$ D) and oral absorption. These in turn along with other properties such as the human serum albumin (HSA) binding impact pharmacokinetic behaviour and drug bioavailability.

### Modelling

In order to build the models, the Random Forest algorithm [23] was chosen which is an ensemble learning method for both classification and regression. The algorithm makes use of bagging and feature randomness to build multiple decision trees (each trained on a random subset of data) and merges them together. The models were trained using the ranger [24] library in the statistical computing environment R [25]. The number of trees used to compute the final average predicted value was set to 500. For each endpoint, the data was split randomly into separate training (80%) and test (20%) sets. A fivefold cross-validation was used to identify the best performing model. In order to rule out any selection bias, we repeated random splitting 3 times and the results were averaged to gain an understanding of the variability. Furthermore, *y*-randomization tests were conducted to assess the robustness of the final model. To address the problem with unequal distribution of samples between classes, data augmentation of the minority class was carried out using the synthetic minority oversampling technique (SMOTE) [26].

For regression models, the performance was assessed using the squared regression coefficient ($R^2$) for the correlation between experimental and predicted values. the root mean squared error (RMSE) and the mean absolute error (MAE). For classification models, metrics that are sensitive to the class imbalance have been used. These include the balanced accuracy (BACC) given by:

1 $$BACC = \frac{1}{m} \sum _i^m \frac{k_i}{n_i}$$

where $k_i$ is the number of correct predictions in class *i*, *m* is the number of classes and $n_i$ is the number of examples in class *i*. In addition, other metrics such as the overall accuracy, the sensitivity (the true positive rate—TPR) and specificity (the true negative rate—TNR) and the area under the curve (AUC) are also reported (see Additional file 1).

Every model has a finite applicability domain (AD) within which its predictions can be trusted. For regression models, we quantify the prediction intervals (95%) using the quantile regression forests approach [27]. Here, a shorter prediction interval indicates the higher stability of prediction. In the case of classification, two values: confidence and credibility are associated with the predicted label based on the conformal prediction framework [28, 29]. While the confidence provides a measure of how likely a prediction is compared to all other possible classifications, the credibility measure (equal to the highest *p*-value of any one of the possible classifications being the true label) provides an indication of how good the training set is for classifying the given example.

## Results and discussion

For the various endpoints, the relevant performance metrics associated with the best fingerprint-based models are summarized in Tables 4 (for classification models) and 5 (for regression models). The complete performance summary for the training and validations sets is listed in Additional file 1: Tables S1 and S2. For all cases, permutation tests confirmed (p-values < 0.001) that the probability that the model was obtained by chance is quite low. Overall, high classification accuracies ($BACC > 0.80$) are obtained for the blood brain barrier permeability, plasma protein binding, CYP450 inhibition (3A4/2C19/1A2/2C9/2C8 isoforms), human intestinal absorption, breast cancer resistance protein inhibition, p-glycoprotein inhibitor/substrate and hemolytic/respiratory toxicity. For some of the other endpoints such as the mitochondrial/urinary tract toxicity, human liver microsomal stability, metabolic intrinsic clearance, AMES mutagenecity, cytotoxicity (multiple cell lines), hERG cardiotoxicity/liability, drug induced liver injury, myelotoxicity, phospholipidosis, rhabdomyolysis, OATP1B1/OATP1B3 inhibition, BSEP and OCT2 inhibition, moderate ($BACC = 0.71 \;\text{to}\; -0.78$) performances were observed. Properties such as skin sensitization, acute oral toxicity, phototoxicity in humans, ototoxicity, choleostasis, hepatic steatosis, and carcinogenecity yielded somewhat average results. In the case of regression models, performances were largely on the poorer side with the exception of $\text {pK}_a$, $\log$ S, $\log$ D, human serum albumin and skin penetration, $R^2_{cv} > 0.70$.

**Table 4:** Performance metrics for the best performing fingerprint-based classification models

Endpoint	FP	Calibration		Validation	
		BACC	AUC	BACC	AUC
Blood brain barrier	PUBCHEM	0.82	0.90	0.81	0.92
Oral bioavailability	PUBCHEM	0.71	0.77	0.71	0.78
Anticommensal effect	PUBCHEM	0.76	0.82	0.74	0.81
CYP450 (1A2)	PUBCHEM	0.85	0.93	0.85	0.93
CYP450 (2C19)	ECFP4	0.81	0.88	0.81	0.89
CYP450 (2C9)	PUBCHEM	0.78	0.88	0.79	0.89
CYP450 (2D6)	FCFP4	0.73	0.86	0.73	0.87
CYP450 (3A4)	FCFP6	0.80	0.89	0.80	0.90
CYP450 (2C8)	PUBCHEM	0.79	0.89	0.77	0.90
HIA	MACCS	0.84	0.89	0.83	0.89
BCRP inhibition	FCFP4	0.89	0.95	0.90	0.96
Metabolic intrinsic clearance	FCFP4	0.74	0.82	0.74	0.84
Human liver microsomal stability	AT2D	0.77	0.83	0.77	0.84
PGP inhibitor	PUBCHEM	0.84	0.91	0.85	0.92
PGP substrate	ASP	0.80	0.87	0.80	0.88
DMSO solubility	ECFP2	0.72	0.78	0.73	0.80
Phosphate buffer solubility	PUBCHEM	0.79	0.87	0.79	0.87
Skin sensitization (LLNA)	PUBCHEM	0.69	0.76	0.67	0.74
Skin sensitization (KeratinSens)	LSTAR	0.64	0.65	0.57	0.60
Skin sensitization (HRIPT)	ECFP0	0.70	0.74	0.67	0.72
Skin sensitization (hCLAT)	MACCS	0.65	0.70	0.61	0.68
Skin sensitization (DPRA)	FCFP4	0.68	0.72	0.68	0.72
Rat acute oral toxicity ($\text {LD}_{{50}}$)	PUBCHEM	0.69	0.78	0.68	0.81
AMES mutagenecity	PUBCHEM	0.79	0.86	0.79	0.87
Cytotoxicity (HepG2)	AT2D	0.78	0.85	0.78	0.85
Cytotoxicity (CRL-7250 cell line)	AT2D	0.79	0.87	0.78	0.86
Cytotoxicity (HACAT cell line)	AT2D	0.77	0.85	0.77	0.85
Cytotoxicity (HEK cell line)	PUBCHEM	0.77	0.87	0.76	0.86
Cytotoxicity (NIK cell line)	PUBCHEM	0.78	0.87	0.78	0.87
DILI	PUBCHEM	0.78	0.86	0.79	0.88
Hemolytic toxicity (saponins)	FCFP6	0.84	0.88	0.85	0.90
hERG cardiotoxicity	FCFP6	0.79	0.86	0.80	0.88
hERG liability	PUBCHEM	0.76	0.87	0.76	0.88
Mitochondrial toxicity	PUBCHEM	0.79	0.90	0.77	0.90
Urinary tract toxicity	FCFP4	0.71	0.77	0.70	0.73
Phototoxicity in vitro	KR	0.70	0.76	0.69	0.80
Phototoxicity human	PUBCHEM	0.69	0.75	0.67	0.75
Toxic myopathy	DFS	0.68	0.74	0.63	0.74
Myelotoxicity	FCFP4	0.72	0.79	0.71	0.80
phospholipidosis	FCFP2	0.78	0.86	0.77	0.88
Cholestasis	RAD2D	0.67	0.73	0.67	0.74
Rhabdomyolysis	MACCS	0.71	0.80	0.70	0.83
Respiratory toxicity	MACCS	0.82	0.88	0.82	0.89
Ototoxicity	PUBCHEM	0.69	0.74	0.67	0.72
MATE1	DFS	0.64	0.67	0.65	0.65
Hepatic steatosis	MACCS	0.63	0.67	0.59	0.68
Carcinogenecity	PUBCHEM	0.67	0.71	0.68	0.75
OATP1B1 inhibition	ECFP6	0.72	0.80	0.73	0.82
OATP2B1 inhibition	ECFP6	0.67	0.68	0.65	0.70
OATP1B3 inhibition	PUBCHEM	0.74	0.83	0.77	0.87
BSEP inhibition	ECFP4	0.85	0.93	0.88	0.95
OCT2 inhibition	PUBCHEM	0.73	0.81	0.73	0.79
PPB	PUBCHEM	0.82	0.92	0.84	0.92
Elimination half-life Human	ASP	0.75	0.86	0.76	0.88
Elimination half-life Mouse	ECFP2	0.74	0.86	0.72	0.84
Elimination half-life Rat	KR	0.74	0.86	0.74	0.83

The values reported are the balanced accuracies (BACC) and area under the ROC curve (AUC) (average of 3 independent runs) for the calibration/validation sets

**Table 5:** Performance metrics for the best performing fingerprint-based regression models

Endpoint	FP	Calibration			Validation		
		R^2^	RMSE	MAE	R^2^	RMSE	MAE
$\log \ S$	PUBCHEM	0.77	1.15	0.81	0.78	1.12	0.78
Intrinsic clearance ($CL_{int}$)	RAD2D	0.48	0.83	0.65	0.29	1.02	0.82
Skin penetration ($\log \ k_p$)	PUBCHEM	0.73	0.60	0.48	0.75	0.56	0.43
Human serum albumin	AP2D	0.71	0.33	0.23	0.69	0.39	0.26
Human placenta barrier	KR	0.41	0.24	0.20	0.24	0.32	0.22
Cancer potency in mouse ($TD_{50}$)	AT2D	0.33	0.98	0.75	0.27	0.96	0.72
Cancer potency in rat ($TD_{50}$)	AT2D	0.41	1.08	0.83	0.35	1.14	0.87
Steady state volume distribution ($VD_{ss}$)	ASP	0.58	0.44	0.29	0.45	0.51	0.32
Distribution coefficient ($\log \ D$)	PUBCHEM	0.76	0.73	0.53	0.77	0.71	0.50
Fraction unbound in human plasma	PUBCHEM	0.60	0.46	0.35	0.63	0.44	0.34
Fraction unbound in the brain	PUBCHEM	0.48	0.58	0.46	0.56	0.56	0.45
Human liver microsomal clearance	KR	0.51	1.08	0.80	0.56	1.05	0.79
Mouse liver microsomal clearance	AT2D	0.52	1.21	0.92	0.53	1.16	0.88
Rat liver microsomal clearance	KR	0.64	1.08	0.83	0.67	1.01	0.76
CACO-2 permeability	FCFP4	0.44	0.68	0.46	0.42	0.69	0.46
${pK}_a$	ECFP2	0.71	1.85	1.15	0.74	1.78	1.11
MDCK cell line permeability	ECFP4	0.62	0.61	0.44	0.68	0.56	0.39
Human renal clearance	MACCS	0.25	0.54	0.43	0.27	0.53	0.42
Hemolytic toxicity ($\log \ {HD}_{50}$)	ASP	0.68	0.47	0.35	0.68	0.44	0.34

The values reported are the squared correlation ($R^2$), RMSE and MAE (average of 3 independent runs) for the calibration/validation sets

**Fig. 1:**
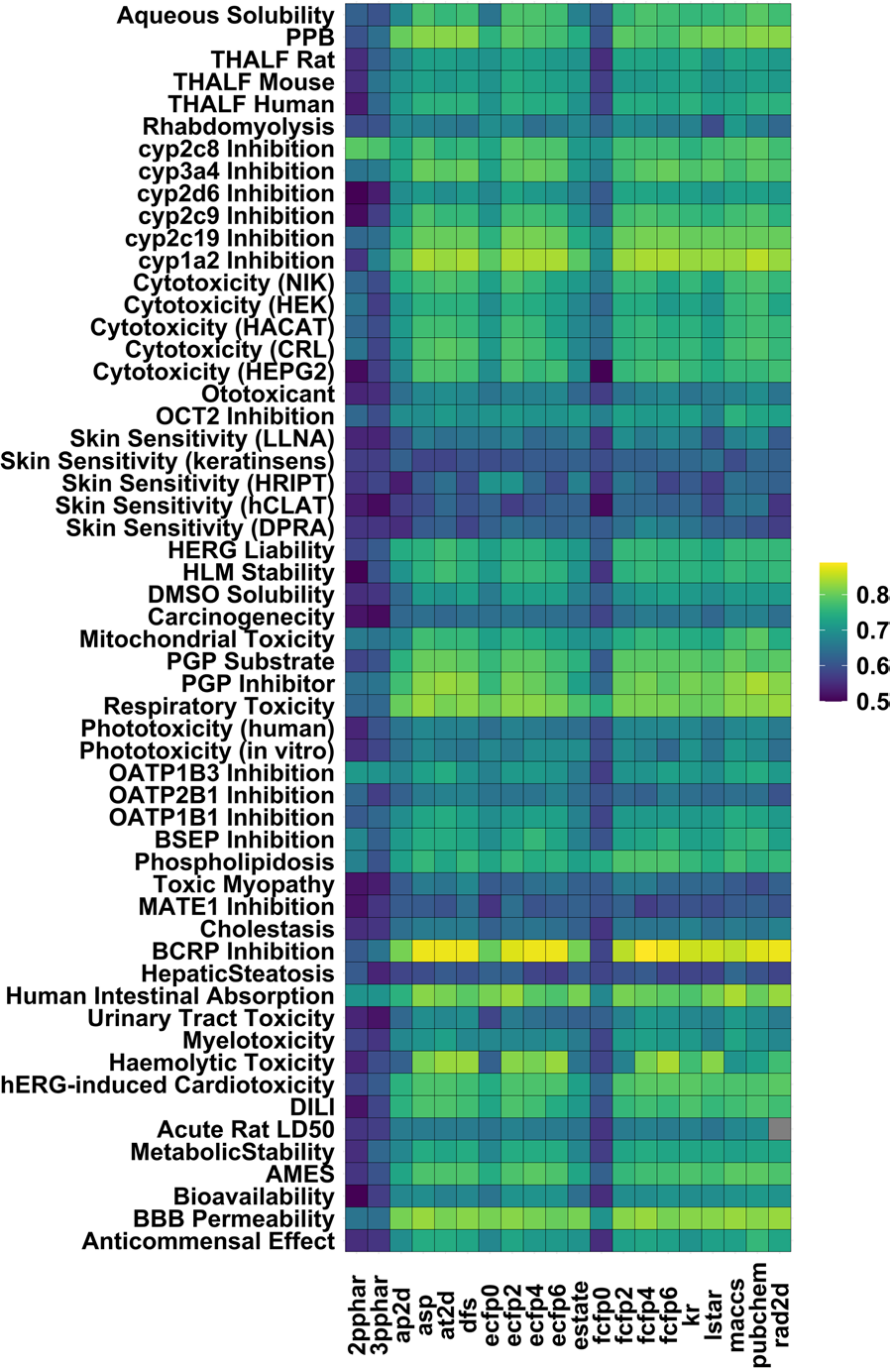
Heatmap showing the cross-validated balanced accuracies (average of 3 independent runs) achieved by different fingerprint-based models for the endpoints studied

**Fig. 2:**
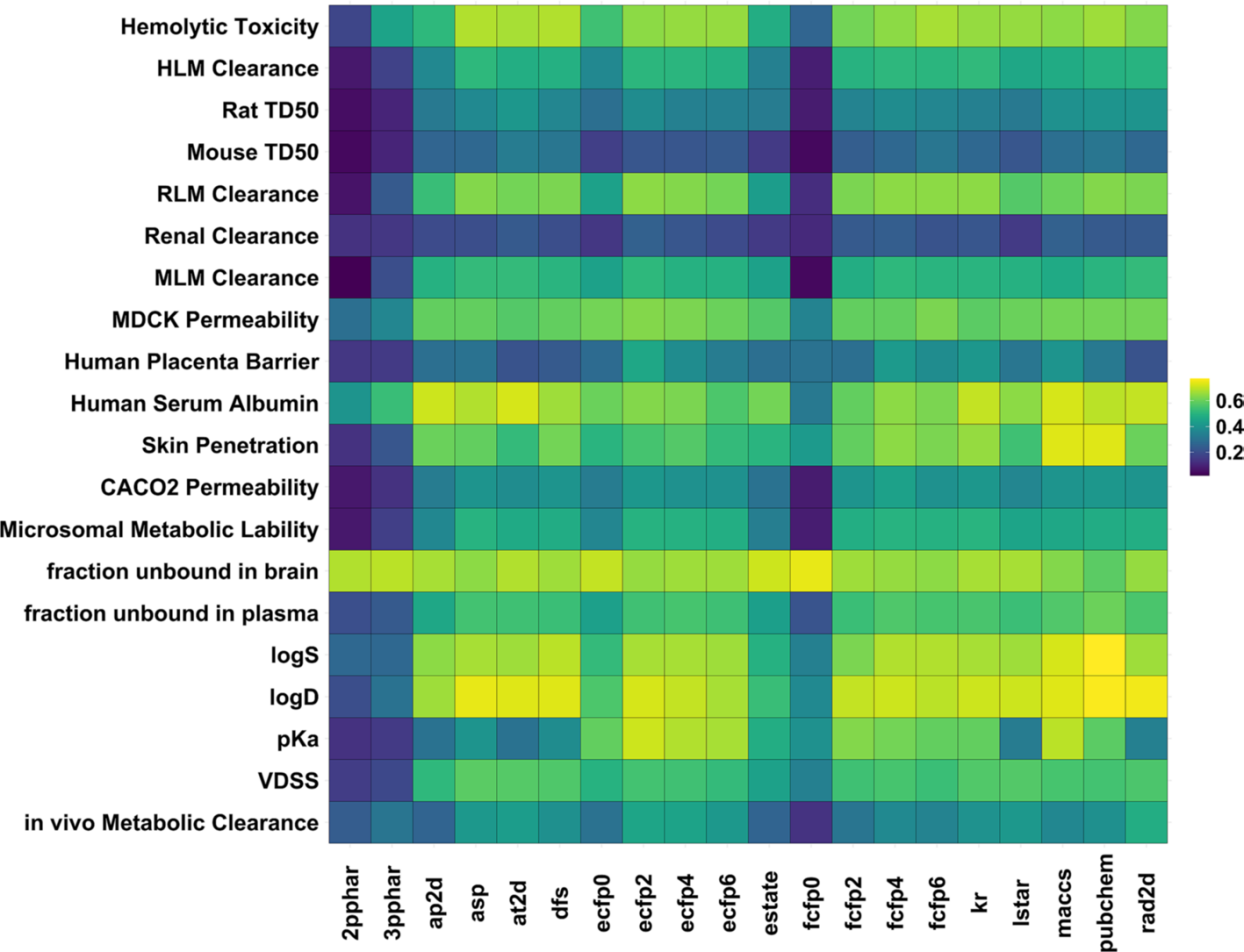
Heatmap showing the cross-validated correlation coefficients (average of 3 independent runs) achieved by different fingerprint-based models for the endpoints studied

To identify which of the fingerprints perform well on the different datasets, we plotted heatmaps (see Figs. 1 and 2) of the balanced accuracies (for classification models) and squared correlations (in the case of regression) obtained for the different endpoints. While the pharmacaphore fingerprints (2PPHAR/3PPHAR) perform poorly on all datasets, fingerprints based on substructure keys (PUBCHEM, MACCS, KR) show moderate to high accuracies for a majority of the modelled endpoints. Although the performances for regression models are somewhat less encouraging, here too the $R^2_{cv}$ for PUBCHEM, ECFP4, and ASP fingerprints yield better models than the other fingerprints tested.

**Fig. 3:**
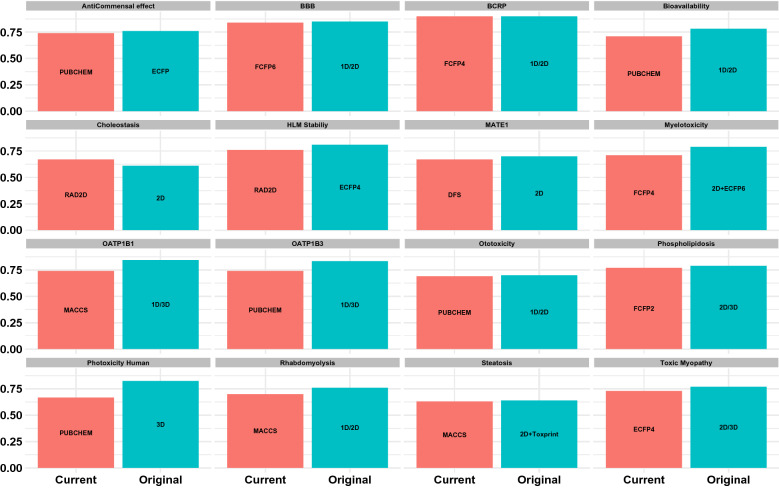
Comparison of the accuracies achieved by the fingerprint based models in this study (“Current”) with those created using standard molecular graph based descriptors (“Original”) published in the literature. For OATP inhibtion, descriptors consist of constitutional, geometrical, electrostatic, and physicochemical indices. For phototoxicity, descriptors contain HOMO-LUMO gaps, spectral integrals, ionization potential, electron affinity and CATS descriptors. For properties such as toxic myopathy and MATE1 inhibition, the values compared are the accuracies and AUCs respectively

We further compared the performances achieved by the fingerprint models with those obtained for the 2D/3D descriptor based approaches. The barplots in Fig. 3 compare the accuracies achieved by the fingerprint models with values reported by the models published earlier. While results for most properties are comparable, for some endpoints such as myelotoxicity, ototoxicity, myopathy accuracies obtained using 2D/3D descriptors are only marginally better. Indeed better results are obtained for rhabdomyolysis, phospholipidosis, phototoxicity with other descriptor based models. For phototoxicity in particular, quantum chemistry-based 3D descriptors are used which can add to the time taken. It must however be pointed out that some of the better performing models take advantage of deep learning. Attempts to improve results for selected properties were carried out using support vector machines. However, the models were not always found to improve on the random forest approach.

For the regression models calculated for selected properties: $\text {pK}_a$, $\log$S, $\log$D, skin penetration, human serum albumin, MDCK permeability $\text {HD}_{{50}}$, we assessed the prediction reliability based on the prediction intervals. Plots of the prediction intervals with respect to the observed response values for the test sets (see Additional file 1: Figure S1) showed that most of the samples lie within the 95% prediction interval which indicates that the constructed prediction intervals are reliable. For classification models, we focused on excluding compounds whose labels are predicted with low confidence and credibility. Thus, different thresholds for *p*-values (0.5, 0.6, 0.7, 0.8, 0.9) were applied and the corresponding fraction of molecules that would be withheld from further testing was recorded. A plot of the overall error rates and the percentage reduction in compounds excluded from further processing (see Additional file 1: Figure S2) shows that for many of the endpoints modelled, the predictive performance is not significantly impacted even at cutoffs of 0.50. Such a strategy that allows for compound selection based on static thresholds for the confidence/credibility offer a way to reduce the number of compounds that typically undergo experimental testing.

## Software usage

FP-ADMET is available as open access software (GNU GPL v3.0) and can be downloaded from https://gitlab.com/vishsoft/fpadmet. Use of FP-ADMET proceeds in two steps (i) fingerprint calculation followed by (ii) predicting the ADMET endpoint of interest. The software is command line driven and is governed by a shell script (*runadmet.sh*) that can be run as:

bash runadmet.sh -f molecule.smi -p ## -a

The input to the script is a file (*molecule.smi*) containing SMILES strings. The ## is a number between 1 (predict Anticommensal Effect) and 56 (predict skin penetration) and corresponds to the prediction task. The results are written to a text file where each line contains molecule name and the predicted response. The “-a” option allows for the calculation of prediction intervals (in the case of regression) and confidence (for classification). For classification, conformal prediction is used to calculate a confidence (how certain the model is that the prediction is a singleton) and a credibility. For example, predicting AMES mutagenecity (task number 4) for a series of molecules produces the following results (see Table 6). The label “inactive” for compound G00001 suggests that the compound is predicted to be non-mutagenic. A confidence value of 0.95 suggests that the classifier is quite certain that the prediction is likely to be a single label. A relatively low value of credibility (0.57) suggests that the compounds like G00001 are not sufficiently represented in the training set and that the user needs to treat the prediction with caution. In the case of regression, a 95% prediction interval (predictions at the 0.025 and 97.5 percentiles for $pK_a$) is calculated and provides a range for the predictions on an individual observation. Narrow prediction intervals indicate a lower uncertainty associated with the prediction.

**Table 6:** Example showing the property ($pK_a$ and anticommensal effect) predictions and associated uncertainties for 3 molecules

Name	Anticommensal effect	Confidence	Credibility	$\hat{pK_a}$	Q = 0.025	Q = 0.975
G00001	Inactive	0.95	0.57	9.62	4.89	11.49
G00002	Active	0.95	0.51	4.41	− 1.60	13.06
G00003	Inactive	0.95	0.57	3.37	1.66	6.10

$Q=0.025$ and $Q=0.975$ are the predictions calculated at percentiles 0.025 and 0.975 and allow for 95% prediction intervals

## Conclusion

In this article, we have evaluated the performance of various molecular fingerprints for predicting a number of ADMET and ADMET-related endpoints. A total of 1500 models were analysed spanning 75 responses and 20 fingerprints. The results show that the machine learning performance using the different fingerprint encodings rival those of traditional descriptor-based methods. Future work will focus on combining different data sets in a multitask modeling approach which has been shown to yield statistically superior results compared with single-task models [12, 30]. In order to facilitate ADMET evaluation, the best performing models have been compiled into an open access software package called FPADMET that can be downloaded from https://gitlab.com/vishsoft/fpadmet.

## Supplementary Information

**Additional file 1.** File contains brief descriptions of the properties modelled, additional performance statistics and figures referred to in the text.
