# Supplementary material for: FP-ADMET: a compendium of fingerprint-based ADMET prediction models
Source: J Cheminform. 2021 Sep 28;13:75. doi: 10.1186/s13321-021-00557-5 (PMC8479898; doi:10.1186/s13321-021-00557-5)
Supplement: Supplementary file 1 — Additional file 1. File contains brief descriptions of the properties modelled, additional performance statistics and figures referred to in the text. [file 13321_2021_557_MOESM1_ESM.pdf]

Supplementary Material for

FP-ADMET: A Compendium of Fingerprint-based ADMET Prediction Models

Vishwesh Venkatraman

Department of Chemistry, Norwegian University of Science and Technology (NTNU), 7491 Trondheim, Norway

1 ADMET Properties Studied

The following sections briefly describe the different ADMET endpoints studied. For details on the experimental protocols, values and units, and data preparation please see the cited articles and the references therein.

1.1 Anticommensal effect on the human gut microbiota (HGM)

Some of the existing drugs that are designed to target human cells can notably alter the composition of the human gut microbiota. This anticommensal effect of the drugs should therefore be avoided. Anticommensal compounds are defined those that can inhibit at least one bacterial strain in the human gut, while a commensal compound is assumed if this compound cannot suppress any one of the 40 typical bacterial strains in their experimental assays. Compounds with anticommensal or commensal effects on human gut microbiota (*in vitro*) are taken from the work of **?**. Zheng et al. [2018] have examined the use of ECFP based approaches to predict the anticommmensal effect with a machine-learning based consensus classification model yielding accuracies of 80% or more.

1.2 Blood Brain Barrier Permeability

Diffusion of drugs across blood brain barrier (endothelium separating the blood from the central nervous system) is quite restrictive. Early assessment of BBB permeation is therefore of great importance, not only for CNS-active agents, but also for drugs that must be forbidden entry into the CNS (to prevent harmful side effects). Conventional *in vitro* experiments such as Caco-2 permeability assays, transepithelial electrical resistance (TEER) assays, Madindarby canine kidney (MDCK) and parallel artificial membrane permeability assays (PAMPA) are used to evaluate BBB permeability. Each compound in the dataset taken from Shaker et al. [2020] and Sushko et al. [2011] has a BBB permeability class (given as "No" and "Yes" for BBB- and BBB+, respectively). Accuracies of around 89% have been achieved using 1D/2D descriptors based on a Light Gradient Boosting Machine learning modelShaker et al. [2020].

1.3 Breast Cancer Resistance Protein Inhibition

The Breast Cancer Resistance Protein (BCRP) is seen to responsible for the multidrug resistance phenomenon observed in some cancers and is also involved in drug-drug interactions in the liver. Here, the dataset used by Jiang et al. [2020] that contains a structurally diverse set of 1098 BCRP inhibitors and 1701 non-inhibitors (based on cell-based assays) has been used for modelling (see also Montanari and Ecker [2014]). In their study, the SVM classifier yielded the best predictions (MCC = 0.812 and AUC = 0.958 for the test set).

1.4 Human Oral Bioavailability

Oral bioavailability (an important pharmacokinetic property F) measures the extent to which the active moiety is absorbed and becomes available in the systemic circulation. Since most drugs

are administered orally, bioavailability decreases and methods that can differentiate between high ($F \geq 50\%$) and low ($F < 50\%$) bioavailability can help reduce undesirable attrition (see Varma et al. [2010] for experimental details). For a set of 1448 molecules, models based on 2D descriptors yielded accuracies of around 80% for both training and test setsFalcón-Cano et al. [2020].

1.5 CYP450 Metabolism

The cytochrome P450 superfamily oxidize steroids, fatty acids, and xenobiotics, and are important for the clearance of various compounds. Adverse side effects of drug–drug interactions induced by human CYP450 inhibition is a major cause for concern. Here, the models predict inhibition of major CYP450 isoforms, namely, 1A2, 2C9, 2C19, 2D6, 2C8, and 3A4. A major data source is the study by Veith et al. [2009] wherein the authors employed a Cytochrome panel assay (`https://pubchem.ncbi.nlm.nih.gov/bioassay/1851`) that used various human CYP450 isozymes to measure the dealkylation of various pro-luciferin substrates to luciferin. Here the compounds labeled "active" are regarded as inhibitors and "inactive" as noninhibitors. Additional data for CYP2C8 inhibition was taken from Zhang et al. [2021], wherein the authors developed fingerprint based classification models achieving AUCs of 0.85 and above.

1.6 DMSO Solubility

Solubility of drug candidates or chemical probes is an important parameter considered by pharmaceutical companies. Insoluble compounds cannot be used in an automatized high-throughput screening (HTS) and therefore experimental measurements cannot be obtained for such compounds. Earlier Tetko et al. [2013] employed machine learning methods for the prediction of solubility in DMSO (solubility in 100% DMSO). The dataset is highly imbalanced with around 1.7–5.8% insoluble compounds. Using different kinds of descriptors balanced accuracies of around 70% were achieved.

1.7 Solubility in phosphate buffer

Data for solubility was obtained from PUBCHEM bioassay database (`https://pubchem.ncbi.nlm.nih.gov/bioassay/1996`) where over 50000 compounds were tested for their solubility in an aqueous buffer (Phosphate Buffered Saline, pH 7.4). The compounds were provided as DMSO stocks and the solubility was measured using quantitative chemiluminescent nitrogen detection. In the assay, compounds with a mean solubility in aqueous buffer > 10 μg/mL at pH 7.4 are considered as active (moderate/high solubility). For this dataset, a Bayesian model using 8 physicochemical descriptors and the FCFP_6 fingerprint achieved an AUC of around 0.84Perryman et al. [2020].

1.8 Fraction Unbound in Human Plasma/Brain

The fraction unbound in plasma is an important determinant of drug efficacy in pharmacokinetic and pharmacodynamic studies. Knowing the value of the unbound drug fraction in the brain is essential for estimating its effects and toxicity on the central nervous system. For a set of 253 measurements ($f_{u,brain}$) taken from literature, Esaki et al. [2019] achieved $R^2 \sim 0.60$ using 2D descriptors. For

the prediction of the fraction unbound in human plasma ($f_{u,plasma}$) a data set of 2738 experimental values was taken from Watanabe et al. [2018]. While Watanabe et al. [2018] focus on classification models (accuracies of 0.80), Yun et al. [2021] focus on regression models obtaining a mean absolute error (MAE) of 6.7% on a subset of the Watanabe dataset.

1.9 Human Intestinal Absorption

Human intestinal absorption (HIA) is an alternative indicator for oral bioavailability to some extent and thus plays an important role in pre-clinical drug evaluation. Experimental data have relied on *In vitro* human colon adenocarcinoma (Caco-2) cell lines and parallel artificial membrane permeability assays. Data from Sushko et al. [2011] and Wang et al. [2017] were merged to yield a set of 1516 compounds. A HIA value of 30% was used as the criterion to divide chemical agents into the good absorption (positive) and the poor-absorption (negative) classes. In their study, models based on 2D descriptors were found to yield AUCs $>$ 0.84 for both training and validation sets.

1.10 Human Liver Microsomal Clearance

Microsomal lability assays are widely employed in drug discovery to identify compounds that will have desirable pharmacokinetics in humans. Here, models have been established to predict the microsomal lability in different species: rat, mouse and humans. The regression models proposed here are based on public data from ChEMBL (*in vitro*) and have been studied by Wenzel et al. [2019]. The authors made use of atom pairs and pharmacophoric donor-acceptor pairs as descriptors and trained deep learning models to achieve R^2 of 0.59, 0.71 and 0.49 for the human, rat and mouse datasets respectively.

1.11 Human Liver Microsomal Stability

The human liver microsomal (HLM) stability assay is commonly used for assessing clearance of chemicals by the human liver. The dataset collated by Liu et al. [2015] contains compound stability data as measured by $T_{1/2}$, against phase I metabolism in human liver microsomes. Here, a threshold value of 30 min was used to categorize a compound as stable ($T\frac{1}{2} > 30$ min) or unstable ($T\frac{1}{2} \leq 30$ min) in an HLM (*in vitro*) assay.

1.12 Hepatocellular organic anion transporting polypeptide Inhibition

Hepatocellular organic anion transporting polypeptides (OATP1B1, OATP1B3, and OATP2B1) are important for liver function and the regulation of the drug elimination process. OATP1B inhibition or induction may be the underlying mechanism of clinically relevant drug-drug interactions and have been linked with hyperbilirubinemia. Bioactivity measurements (K_m and IC_{50} data) were compiled from multiple databases (ChEMBL, the UCSF-FDA TransPortal database, DrugBank, Metrabase and IUPHAR) and other literature sourcesTürková et al. [2018]. A compound was defined as active if the bioactivity was $<$10 μM.

1.13 Drug affinity to Human Serum Albumin

Human serum albumin (HSA) is the most abundant protein in human blood plasma. Binding of drugs to HSA strongly influences their pharmacokinetic behaviour and is associated with drug-drug interactions and drug safety. The model predicts $\log K_{HSA}$: the binding constant obtained from the retention time on an immobilized HSA column using affinity chromatographySerra et al. [2019], Ciura et al. [2020].

1.14 Drug Interactions With the Human Bile Salt Export Pump

Drug-induced liver injury is frequently associated with inhibition of bile salt transporters such as the bile salt export pump (BSEP) which is mainly expressed in the liver. In the dataset taken from McLoughlin et al. [2021] compounds were categorized as BSEP inhibitors if their IC_{50}s (*in vitro* assays) were $\leq 10\ \mu$M and as non-inhibitors if $IC_{50} > 50\ \mu$MMcLoughlin et al. [2021].

1.15 Drug transport across the human placenta barrier

The human placental barrier permeability of drugs is essential for drug safety during pregnancy. The regression model predicts placental transfer expressed as clearance index values that are measured using *ex vivo* human placental perfusion methodGiaginis et al. [2009].

1.16 *in Vivo* Intrinsic Clearance

Hepatic metabolism is a key determinant for drug clearance. Predicting the intrinsic metabolic clearance CL_{int} would go a long way in assessing drug safety and pharmacodynamics. The CL_{int} data were negative-log transformed prior to modeling. Data Analysis was conducted on the CL_{int} values obtained from an extensive pharmacokinetic data set compiled by Varma et al. [2010]. To distinguish drug metabolic stability, i.e., those with $CL_{int} < 1500$ mL/min were considered stableHsiao et al. [2013].

1.17 logD

The lipophilicity at pH = 7.4 (the physiological pH of blood serum), is a major determinant of various ADMET parameters of drug candidates. It is said to be a better descriptor of the lipophilicity of a molecule. Relevant data was taken from Fu et al. [2019], Sushko et al. [2011] whose XGBoost model yielded R^2 of 0.90.

1.18 logS

The aqueous solubility of compounds has a key role in various domains. The database AqSolDB-Sorkun et al. [2019] contains experimental solubility values that were all standardized to the LogS units. Compounds can be classified according to solubility values (LogS):

1. Compounds with 0 and higher solubility values are highly soluble

2. Those in the range of 0 to -2 are soluble
3. Those in the range of -2 to -4 are slightly soluble
4. Insoluble if less than -4

1.19 CACO-2 Permeability/Madin-Darby canine kidney cell line permeability

The absorption of oral drugs occurs in the small intestine where the drug has to traverse the semipermeable intestinal columnar epithelial cells. *In vitro* permeability assays mimic the oral absorption process. Examples include Caco-2 (human colon adenocarcinoma), MDCK (Madin-Darby canine kidney). For MDCK, the model predicts MDCK permeability coefficientsSushko et al. [2011] (apparent permeability, P_{app}). A second regression model proposed here is based on public data containing the apparent permeability coefficient (P_{app}) from ChEMBL (*in vitro*) and has been previously studied by Wenzel et al. [2019]. Prior to modelling, the P_{app} values were log transformed.

1.20 Metabolic Intrinsic Clearance

A key consideration at the screening stages of drug discovery is *in vitro* metabolic stability, often measured in human liver microsomes. The intrinsic clearance (CL_{int}) measurements for over 5000 compounds were obtained from ChEMBL. Only those data that were measured at 37 degrees and pH 7.4, in the absence of inhibitors, and in the presence of reduced NADPH as a cofactor were retainedEsaki et al. [2018]. Classification models were created for CL_{int} based on:

1. stable if $CL_{int} < 20$ mL/min/mg
2. moderate if $20 < CL_{int} < 300$ mL/min/mg
3. unstable if $CL_{int} \geq 300$ mL/min/mg

1.21 Multidrug and Toxin Extrusion Transporter 1 Inhibition

The human multidrug and toxin extrusion (MATE) transporter 1 contributes to the tissue distribution and excretion of many drugs. Inhibition of MATE1 can impact potential drug–drug interactions and cause alterations in drug exposure and accumulation in various tissues. Data for MATE1 inhibitors was compiled based on high-throughput screen using a fluorescent probe ASP+. Compounds that exhibited 50% or more inhibition of ASP+ uptake at 20 μM were considered as strong inhibitors (hits) of MATE1Wittwer et al. [2013].

1.22 Organic Cation Transporter 2 inhibition

The kidney represents an important site for drug-drug interactions. Most registered drugs are eliminated via the kidneys. Hepatic metabolism is usually followed by renal excretion of the formed

metabolites. Consequently, inhibition of renal drug transport can result in significantly altered systemic levels of the parent drug molecule and of potentially active metabolites, with ultimate effects on the drug's pharmacological and toxicological profile. Here, the data was taken from Kido et al. [2011], who screened a library of 910 prescription drugs and drug-like compounds using a high-throughput assay of renal organic cation transport. Compounds that decreased ASP+ transport or more at 20 μM were considered as OCT2 inhibitors.

1.23 Substrates/inhibitors of P-glycoprotein

P-glycoprotein (P-gp) has significant impact on the multidrug resistance and pharmacokinetics of drugsChen et al. [2012]. Compounds actively transported by P-gp are classified as substrates, whereas those that compromise the transporting function of P-gp are classified as inhibitors. Data was sourced from Sushko et al. [2011] and the compounds were divided into substrates ("P") and non-substrates ("N") based on the work byEsposito et al. [2020]: compounds with an efflux ratio $ER \geq 5$ were classified as substrates while compounds with an efflux ratio $ER \leq 1$ were classified as non-substrates. To distinguish between P-gp substrate or inhibitor, a second dataset was again compiled from Sushko et al. [2011] and Wang et al. [2019]. Here, the collected data refer to the inhibition of transport of an ABCB1 probe substrate in a cell line expressing PgpBroccatelli et al. [2011], Rautio et al. [2006]. Compounds with IC50 value higher than 100 μM are considered noninhibitors.

1.24 Prediction of pK_a

The acid–base dissociation constant, pK_a, is a key parameter to define the ionization state of a compound and directly affects its biopharmaceutical profile. pK_a values were taken from Mansouri et al. [2019] and Sushko et al. [2011].

1.25 Human plasma protein binding

Plasma protein binding (PPB) is a key player of drug ADME. Drugs with high PPB, especially those with a narrow therapeutic window, tend to be associated with safety issues and adverse effects. In order to determine the extent of PPB of new drug candidates, the data Sushko et al. [2011], Yuan et al. [2020] was divided into three sets of:

1. high binding level (PPB>0.8)
2. moderate binding level (PPB = 0.4-0.8)
3. low binding level (PPB<0.4)

1.26 Human Renal Clearance

Renal clearance (CLr) plays an important role in the elimination of drugs. The dataset includes 636 compounds where the CLr was determined as the ratio of the unchanged amount excreted in urine to the area under the plasma concentration-time curve following intravenous or oral administration

of the compounds. While the paper by Chen et al. [2020], focuses more on a classification model, we have retained the regression approach.

1.27 Skin Sensitization

For regulatory acceptance of industrial chemicals, the potential for substances to cause skin sensitization needs to assessed. The skin permeability of an active ingredient for instance, can be impacted by its ionization in a dose solution. Models have been developed with *in vitro*, *in chemico*, mice and human *in vivo* data, to predict human effects. Here, different models were developed for

1. the direct peptide reactivity assay (DPRA)
2. KeratinoSens
3. human cell line activation (h-CLAT)
4. mouse local lymph node assay (LLNA)
5. human repeat insult patch test (HRIPT)

Borba et al. [2020] developed a consensus naive Bayes model that predicts human effects using *in vitro*, *in chemico*, and mice and human *in vivo* data. Other skin related parameters include experimentally derived permeability data (obtained through *in vitro* diffusion studies of excised human skin) on diverse compounds taken from Lindh et al. [2017] and Baba et al. [2017]. The model predicts the rate of transdermal delivery i.e. the permeation rate (log K_p) of chemical compounds through human skin.

1.28 Cancer potency/Carcinogenicity

To assess the cancer potency of genotoxic impurities in drug substances, Bercu et al. [2010] compiled TD_{50} data of rats and mice. The TD_{50} values in mg/kg/day were converted to logarithmic form (pTD_{50}). Here, $pTD_{50} > 4.53$ are considered to be potent. A second dataset taken from Zhang et al. [2017], contains compounds that are divided into carcinogens and non-carcinogens. This data was originally extracted from the Carcinogenic Potency Database(`https://www.nlm.nih.gov/databases/download/cpdb.html`) and includes chronic, long-term animal cancer tests (both positive and negative for carcinogenicity).

1.29 Steady state volume distribution

The steady state volume of distribution (VD_{ss}), is a pharmacokinetic parameter representing an individual drug's propensity to either remain in the plasma or redistribute to other tissue compartments. 1. High VD_{ss}: More distribution to other tissue 2. Low VD_{ss} : Less distribution to other tissues. Data for human VD_{ss} (from IV administration) was obtained from Sushko et al. [2011], Lombardo and Jing [2016], Simeon et al. [2019].

1.30 LD_{50} rat acute oral toxicity

The median lethal dose for rodent oral acute toxicity (LD_{50}) is required to categorize chemicals in terms of the potential hazard posed to human health after acute exposure. The data containing a large list of rat acute oral LD50 measurements on $\sim$ 12000 chemicals is sourced from the U.S. Environmental Protection Agency (EPA) National Center for Computational Toxicology (NCCT)Sushko et al. [2011], Kleinstreuer et al. [2018], Gadaleta et al. [2019]. Here, we use the EPA hazard classification EPA's 4-category hazard classification:

1. Category I (LD_{50} $\leq$50 mg/kg) is the highest toxicity category
2. Category II (moderately toxic) includes chemicals with $50 < LD_{50} \leq 500$ mg/kg
3. Category III (slightly toxic) includes chemicals with $500 < LD_{50} \leq 5000$ mg/kg
4. Safe chemicals $LD_{50} > 5000$ mg/kg are included in Category IV

1.31 AMES mutagenecity

Mutagenecity is a prime indicator of toxicity that can cause mutations in the DNA of the test organism. The Ames test (Salmonella/Microsome test) has been employed as an indicator of the carcinogenic potential in mammals. Mutagenecity data for over 9000 compounds were collated by combining data from Sushko et al. [2011] and Xu et al. [2012]. The classification model predicts whether the given compound is mutagenic.

1.32 Cytotoxicity

Cytotoxicity is the degree to which a compound causes damage to cells. Chemical-induced cytotoxicityLee et al. [2019], Sun et al. [2020] was experimentally determined using the CellTiter-Glo (CTG) technology on four different cell lines:

1. Human embryonic kidney 293 cell (HEK 293)
2. NIH/3T3 (3-day transfer, inoculum 3×10^5 cells)
3. CRL-7250
4. HaCat (Cultured Human Keratinocyte cells)

Active and inactive compounds were identified according to their curve classes and measured AC_{50} values. Additional data of *in vitro* toxicity against HepG2 cells was taken from the ChEMBL bioactivity database**?**. Here, compounds with $IC_{50} < 10\mu$M in the assay were considered as cytotoxic (positive class) and non-toxic compounds as negatives.

1.33 Drug induced Liver injury (DILI)

Early stage discovery of DILI is much desired and can be divided into 3 main classes:. hepatocellular, mixed, and cholestatic liver injury. Experimental data is largely based on *in vitro* models ranging from simple plate-based methods such as the B-CLEAR transporter assay, or microscope imaging-based assays to those using multiple parameters to characterise the various facets of DILI. The classification model focuses on distinguishing between DILI-positive (drugs with adverse hepatic effects) and DILI-negative compounds. DILI has several manifestations:

Drug-induced Choleostasis Cholestatic liver injury i.e. cholestasis, is the disruption of the bile flow. In the study by Kotsampasakou and Ecker [2017], any compound that was negative for DILI was also considered negative for choleostasis (since choleastasis is a possible manifestation for DILI).

Myopathy Drug-induced toxic myopathy (rare adverse drug reaction) can cause skeletal muscle damage and pain. The Hazardous Substances Data Bank and DrugBank were used as primary sources for identifying compounds known to demonstrate adverse effect of toxic myopathyHu and Yan [2011]. Another related muscle related issue is that of drug-induced rhabdomyolysis which causes death of muscle fibers and release of their contents into the bloodstream. Approved drugs in DrugBank were used as negative data, while data on rhabdomyolysis-inducing drugs were collated from multiple studies (see Cui et al. [2019]).

Hemolytic Toxicity This form of toxicity can cause lysis of the erythrocyte membrane and subsequent release of hemoglobin into blood plasma, leading to multiple acute and chronic adverse effects. Two models have been proposed (i) Prediction of hemolytic toxicity of small molecules with experimental values of HD_{50}Zheng et al. [2020] (dose causing 50% of maximum hemolysis experimentally measured on erythrocytes in hemolytic assays). log HD_{50} was treated as a response variable. (ii) hemolytic toxicity of saponinsZheng et al. [2019] (compounds bearing a hydrophobic steroid/triterpenoid moiety and hydrophilic carbohydrate branches). The compounds however can have deleterious toxicity that impedes their further applications in medicine. Hemolytic saponins were identified as those with $HD_{50} < 200$ μM.

Myelotoxicity This form of toxicity usually leads to decrease in the production of platelets, red cells, and white cells. In the study byZhang et al. [2015], non-myelotoxic agents were defined as those that were (1) widely used in the clinic, (2) had no toxicity warnings from the U.S. Food and Drug Administration, and (3) had no adverse effect on hematopoiesis.

Mitochondrial Toxicity Mitochondria play a central role in maintaining cellular homeostasis. A side effect of certain antiretroviral drugs causes the mitochondria of a body's cells to become damaged or decline significantly in number. Hemmerich et al. [2020] compiled data from the ChEMBL release 22 merged with a confirmatory assay from the Tox21 dataset for mitochondrial membrane potential disruption (AID=720637). Balanced accuracies of 0.89 were obtained with a deep learning model.

Drug-induced Ototoxicity Some drugs currently in clinical use can cause cellular degeneration of cochlear and/or vestibular system, leading to temporary or permanent hearing loss or other

ear problems. The naive Bayes model proposed by Zhang et al. [2020] was based on the 7 molecular descriptors and ECFP fingerprints produced 90.2% overall prediction accuracy.

Respiratory Toxicity Drug-induced respiratory toxicity can cause significant toxicological issues. Clinical symptoms mainly include wheezing, bronchoconstriction, asthmatic attacks and pulmonary eosinophilia. Zhang et al. [2018] compiled the dataset by merging *in vitro*data from Pneumotox, (`http://www.pneumotox.com`) and Dik et al. [2015, 2014] (using bronchial epithelial cell line 16HBE14o-). the chemicals are classified as either respiratory sensitizers or non-sensitizers (respiratory irritants + non-sensitizers).

Drug Phototoxicity Photosensitization is a significant safety concern for drug development in certain chronic disease indications where patients are exposed to sunlight. Drug photosensitization (photoirritation/photoallergy) can result in side effects such as rashes or dermatitis. Here we focus on two models: 1. *in vitro* phototoxicity (3T3 NRU phototoxicity reports) 2. human photosensitization (clinical photosensitization alerts) Schmidt et al. [2019].

Urinary tract toxicity Nephrotoxicity is the poisonous effect of some substances, both toxic chemicals and medications, on kidney function. According to the regulations of the U.S. Environmental Protection Agency, compounds were categorized into two classes: 1. Compounds with LD50 $\leq$ 500 mg/kg were defined as urinary tract toxicants 2. Compounds with LD50 $>$ 500 mg/kg were set as urinary tract non-toxicantsLei et al. [2017]

Phospholipidosis The drug-induced accumulation of phospholipids in lysosomes of various tissues is predominantly observed after prolonged exposure. Fusani et al. [2017] provide a dataset combining compounds screened *in vitro* with additional data set described in Orogo et al. [2012].

Hepatic Steatosis Fatty liver also known as hepatic steatosis, occurs when fat builds up in the liver. It is a rare form of DILI. Data contains 1041 compounds for which *in vivo* rodent studies with repeated oral exposure has been carried outJain et al. [2020].

1.34 hERG-induced cardiotoxicity/hERG liability

Blockade of the human ether-á-go-go-related gene (hERG) channel by small molecules leads to fatal cardiotoxicity. For the assessment of hERG liabilities, a little over 9000 compounds was compiled by integrating the hERG bioactivity data from the ChEMBL database and additional data obtained by high-throughput thallium flux assay (that detects inhibition of the hERG channel by measuring the flow of thallium)Siramshetty et al. [2020]. A second dataset was used for the prediction of hERG blockersCai et al. [2019] wherein experimental hERG blockage bioactivities were collated from (i) patch-clamp measurements from ChEMBL bioactivity database; (ii) radioligand binding measurements on mammalian and nonmammalian cell lines; (iii) hERG K+ channel binding affinity, and (iv) literature-derived data. The compounds whose $IC_{50} \leq 10\mu$M were regarded as hERG blockers.

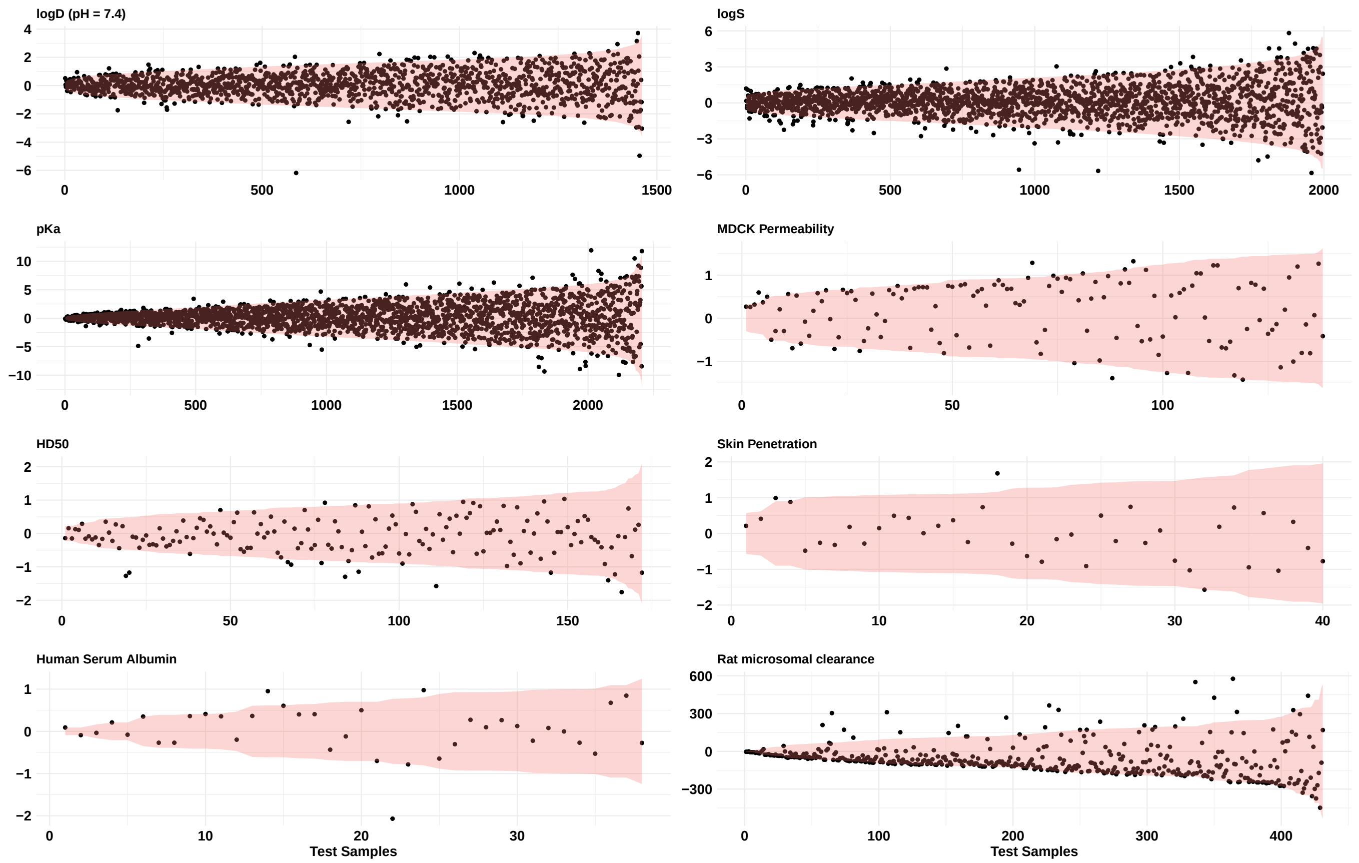

Figure S1: Plot shows the prediction intervals and the original response values. We see that for most of the models, a majority of the samples lie within the 95% prediction interval. For ease of comparison, the data has been center such that the mean of the prediction interval is at 0.0.

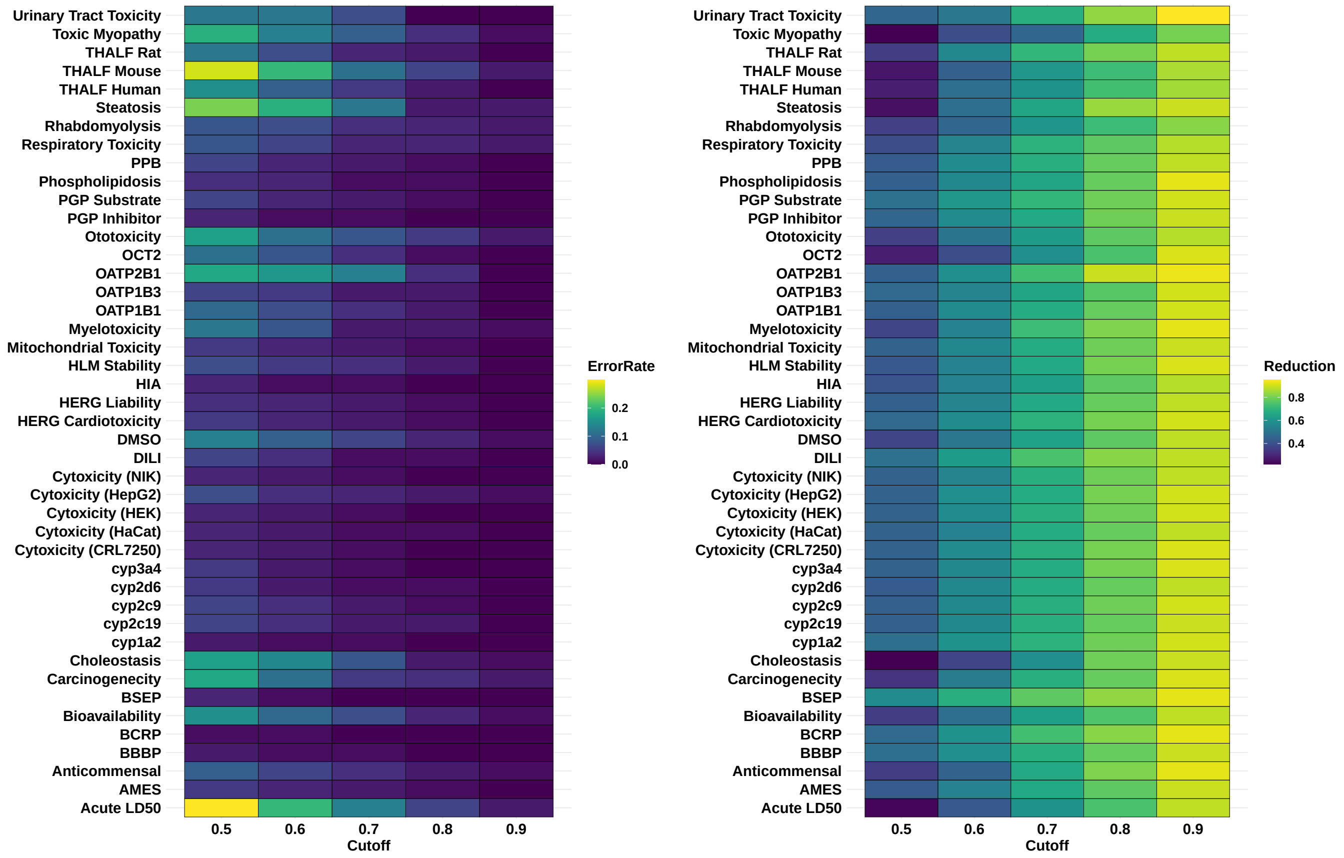

Figure S2: For the test sets corresponding to the different endpoints, each prediction was accompanied by a confidence and credibility. Applying different thresholds, the top ranked compounds are assessed in terms of the error rate (figure on the left). Correspondingly on the right, we see the reduction in the number of compounds that are excluded from further testing. As the cutoff is lowered the errors increase.

Table S1: *Performance of classification models in training and validation sets.*

Endpoint	FP	Calibration								Validation							
		κ	BACC	ACC	Se	Sp	Precision	Recall	AUC	κ	BACC	ACC	Se	Sp	Precision	Recall	AUC
Anticommensal Effect	2PPHAR	0.11	0.55	0.66	0.87	0.22	0.69	0.87	0.57	0.04	0.52	0.63	0.85	0.18	0.68	0.85	0.52
	3PPHAR	0.15	0.56	0.68	0.91	0.22	0.70	0.91	0.63	0.09	0.54	0.67	0.93	0.15	0.69	0.93	0.62
	AP2D	0.40	0.69	0.74	0.84	0.55	0.79	0.84	0.76	0.44	0.71	0.77	0.88	0.53	0.79	0.88	0.80
	ASP	0.48	0.74	0.76	0.80	0.68	0.84	0.80	0.82	0.53	0.76	0.80	0.88	0.64	0.83	0.88	0.84
	AT2D	0.48	0.74	0.76	0.80	0.69	0.84	0.80	0.80	0.47	0.74	0.76	0.81	0.66	0.83	0.81	0.81
	DFS	0.44	0.73	0.75	0.80	0.65	0.82	0.80	0.80	0.52	0.76	0.79	0.84	0.68	0.84	0.84	0.82
	ECFP0	0.36	0.68	0.71	0.75	0.62	0.80	0.75	0.74	0.39	0.70	0.72	0.77	0.64	0.81	0.77	0.76
	ECFP2	0.48	0.73	0.77	0.85	0.61	0.82	0.85	0.80	0.40	0.69	0.75	0.87	0.50	0.78	0.87	0.80
	ECFP4	0.44	0.71	0.76	0.87	0.56	0.80	0.87	0.80	0.47	0.72	0.78	0.90	0.55	0.80	0.90	0.80
	ECFP6	0.45	0.71	0.77	0.88	0.54	0.80	0.88	0.79	0.45	0.70	0.78	0.92	0.49	0.79	0.92	0.79
	ESTATE	0.38	0.70	0.71	0.72	0.68	0.82	0.72	0.76	0.33	0.68	0.69	0.71	0.64	0.80	0.71	0.76
	FCFP0	0.11	0.55	0.63	0.79	0.31	0.70	0.79	0.58	0.09	0.54	0.65	0.87	0.21	0.69	0.87	0.59
	FCFP2	0.45	0.72	0.76	0.83	0.62	0.82	0.83	0.80	0.40	0.69	0.75	0.85	0.54	0.79	0.85	0.76
	FCFP4	0.47	0.73	0.77	0.85	0.60	0.81	0.85	0.80	0.42	0.70	0.76	0.88	0.51	0.79	0.88	0.80
	FCFP6	0.46	0.72	0.78	0.89	0.55	0.80	0.89	0.79	0.45	0.72	0.77	0.87	0.56	0.80	0.87	0.80
	KR	0.43	0.70	0.76	0.86	0.54	0.79	0.86	0.78	0.38	0.68	0.74	0.86	0.49	0.78	0.86	0.78
	LSTAR	0.46	0.72	0.77	0.86	0.58	0.81	0.86	0.79	0.45	0.71	0.78	0.91	0.50	0.79	0.91	0.80
	MACCS	0.45	0.72	0.76	0.83	0.62	0.81	0.83	0.79	0.44	0.72	0.76	0.85	0.58	0.80	0.85	0.81
	PUBCHEM	0.53	0.76	0.79	0.86	0.66	0.84	0.86	0.82	0.50	0.74	0.78	0.86	0.62	0.82	0.86	0.81
	RAD2D	0.48	0.73	0.78	0.88	0.58	0.81	0.88	0.81	0.49	0.73	0.79	0.89	0.58	0.81	0.89	0.83
BCRP Inhibition	2PPHAR	0.18	0.61	0.59	0.66	0.55	0.39	0.66	0.64	0.06	0.54	0.59	0.31	0.77	0.21	0.31	0.63
	3PPHAR	0.33	0.65	0.70	0.41	0.89	0.71	0.41	0.80	0.33	0.65	0.70	0.43	0.87	0.69	0.43	0.79
	AP2D	0.62	0.81	0.82	0.80	0.83	0.76	0.80	0.89	0.65	0.83	0.83	0.81	0.85	0.77	0.81	0.90
	ASP	0.77	0.88	0.89	0.84	0.93	0.88	0.84	0.94	0.77	0.88	0.89	0.83	0.93	0.89	0.83	0.95
	AT2D	0.76	0.88	0.88	0.86	0.90	0.85	0.86	0.94	0.77	0.88	0.89	0.85	0.92	0.87	0.85	0.94
	DFS	0.76	0.88	0.89	0.84	0.92	0.87	0.84	0.93	0.79	0.89	0.90	0.84	0.93	0.89	0.84	0.95
	ECFP0	0.59	0.80	0.80	0.81	0.79	0.72	0.81	0.88	0.58	0.80	0.80	0.80	0.79	0.71	0.80	0.88
	ECFP2	0.75	0.87	0.88	0.85	0.90	0.85	0.85	0.94	0.73	0.86	0.87	0.81	0.91	0.86	0.81	0.95
	ECFP4	0.76	0.88	0.89	0.84	0.92	0.87	0.84	0.94	0.74	0.86	0.88	0.81	0.92	0.87	0.81	0.93
	ECFP6	0.77	0.88	0.89	0.82	0.94	0.89	0.82	0.94	0.78	0.88	0.90	0.82	0.95	0.91	0.82	0.95
	ESTATE	0.60	0.81	0.80	0.81	0.80	0.72	0.81	0.88	0.59	0.80	0.80	0.82	0.78	0.71	0.82	0.88
	FCFP0	0.16	0.58	0.62	0.38	0.77	0.52	0.38	0.62	0.12	0.56	0.60	0.36	0.76	0.49	0.36	0.58
	FCFP2	0.70	0.85	0.86	0.83	0.88	0.81	0.83	0.92	0.71	0.85	0.86	0.80	0.90	0.84	0.80	0.94
	FCFP4	0.78	0.89	0.89	0.85	0.92	0.87	0.85	0.95	0.81	0.90	0.91	0.88	0.93	0.89	0.88	0.96
	FCFP6	0.78	0.88	0.89	0.84	0.93	0.88	0.84	0.94	0.78	0.88	0.90	0.83	0.94	0.90	0.83	0.94
	KR	0.72	0.86	0.87	0.81	0.91	0.85	0.81	0.93	0.77	0.88	0.89	0.84	0.92	0.87	0.84	0.94
	LSTAR	0.74	0.86	0.88	0.79	0.93	0.89	0.79	0.93	0.76	0.87	0.89	0.80	0.94	0.90	0.80	0.93
	MACCS	0.69	0.85	0.85	0.83	0.86	0.80	0.83	0.93	0.71	0.86	0.86	0.84	0.88	0.82	0.84	0.94
	PUBCHEM	0.75	0.87	0.88	0.85	0.90	0.85	0.85	0.94	0.76	0.88	0.89	0.83	0.92	0.87	0.83	0.94
	RAD2D	0.76	0.88	0.89	0.83	0.92	0.87	0.83	0.94	0.78	0.89	0.90	0.86	0.93	0.88	0.86	0.95
Oral Bioavailability	2PPHAR	0.01	0.50	0.53	0.93	0.08	0.53	0.93	0.50	-0.03	0.48	0.51	0.93	0.04	0.52	0.93	0.49
	3PPHAR	0.14	0.57	0.58	0.85	0.28	0.57	0.85	0.63	0.17	0.58	0.60	0.86	0.30	0.58	0.86	0.67
	AP2D	0.35	0.68	0.68	0.70	0.65	0.69	0.70	0.73	0.34	0.67	0.67	0.76	0.58	0.67	0.76	0.75
	ASP	0.37	0.69	0.69	0.69	0.68	0.71	0.69	0.76	0.43	0.71	0.72	0.73	0.69	0.73	0.73	0.79
	AT2D	0.34	0.67	0.67	0.61	0.72	0.72	0.61	0.75	0.33	0.67	0.67	0.63	0.71	0.71	0.63	0.75
	DFS	0.36	0.68	0.68	0.71	0.66	0.70	0.71	0.75	0.38	0.68	0.69	0.83	0.54	0.67	0.83	0.76
	ECFP0	0.30	0.65	0.65	0.63	0.67	0.68	0.63	0.71	0.35	0.67	0.67	0.64	0.71	0.71	0.64	0.74
	ECFP2	0.40	0.70	0.70	0.77	0.62	0.70	0.77	0.76	0.43	0.71	0.72	0.80	0.63	0.71	0.80	0.79
	ECFP4	0.40	0.70	0.70	0.72	0.68	0.72	0.72	0.77	0.38	0.69	0.69	0.73	0.66	0.70	0.73	0.76
	ECFP6	0.39	0.70	0.70	0.76	0.63	0.70	0.76	0.76	0.37	0.68	0.69	0.78	0.58	0.68	0.78	0.76
	ESTATE	0.29	0.64	0.64	0.65	0.63	0.67	0.65	0.70	0.26	0.63	0.63	0.65	0.61	0.65	0.65	0.69
	FCFP0	0.11	0.55	0.57	0.79	0.32	0.57	0.79	0.62	0.11	0.55	0.57	0.79	0.32	0.57	0.79	0.63
	FCFP2	0.39	0.69	0.70	0.74	0.65	0.70	0.74	0.76	0.37	0.68	0.69	0.79	0.58	0.68	0.79	0.76
	FCFP4	0.39	0.69	0.70	0.77	0.62	0.70	0.77	0.76	0.37	0.68	0.69	0.79	0.57	0.68	0.79	0.76
	FCFP6	0.41	0.70	0.70	0.75	0.65	0.71	0.75	0.77	0.36	0.68	0.68	0.74	0.61	0.68	0.74	0.75
	KR	0.39	0.69	0.70	0.78	0.61	0.69	0.78	0.76	0.43	0.71	0.72	0.79	0.64	0.71	0.79	0.79
	LSTAR	0.39	0.69	0.70	0.74	0.64	0.70	0.74	0.76	0.47	0.73	0.74	0.80	0.67	0.73	0.80	0.78
	MACCS	0.40	0.70	0.70	0.72	0.67	0.71	0.72	0.77	0.38	0.69	0.69	0.74	0.64	0.70	0.74	0.76
	PUBCHEM	0.42	0.71	0.71	0.76	0.67	0.72	0.76	0.77	0.42	0.71	0.71	0.78	0.64	0.71	0.78	0.78
	RAD2D	0.40	0.70	0.70	0.81	0.59	0.69	0.81	0.77	0.40	0.70	0.70	0.78	0.61	0.70	0.78	0.77
CYP2C8 Inhibition	2PPHAR	0.58	0.79	0.80	0.85	0.72	0.81	0.85	0.87	0.48	0.74	0.75	0.82	0.65	0.77	0.82	0.84
	3PPHAR	0.55	0.78	0.79	0.83	0.72	0.81	0.83	0.86	0.55	0.77	0.78	0.85	0.68	0.79	0.85	0.86
	AP2D	0.46	0.73	0.75	0.86	0.59	0.75	0.86	0.82	0.46	0.72	0.75	0.85	0.59	0.75	0.85	0.82
	ASP	0.56	0.78	0.79	0.88	0.67	0.79	0.88	0.87	0.57	0.77	0.80	0.90	0.65	0.78	0.90	0.90
	AT2D	0.51	0.74	0.77	0.88	0.61	0.76	0.88	0.86	0.46	0.72	0.75	0.87	0.58	0.75	0.87	0.86
	DFS	0.54	0.76	0.79	0.89	0.63	0.78	0.89	0.86	0.57	0.78	0.80	0.89	0.67	0.79	0.89	0.86
	ECFP0	0.47	0.73	0.74	0.80	0.66	0.77	0.80	0.82	0.37	0.69	0.70	0.74	0.63	0.74	0.74	0.78

Continued on next page

Table S1 – *Continued from previous page*

Endpoint	FP	Calibration												Validation			
		κ	BACC	ACC	Se	Sp	AUC	κ	BACC	ACC	Se	Sp	AUC				
	ECFP2	0.58	0.79	0.80	0.86	0.72	0.82	0.86	0.87	0.53	0.77	0.77	0.81	0.72	0.80	0.81	0.87
	ECFP4	0.57	0.78	0.80	0.88	0.68	0.80	0.88	0.88	0.58	0.78	0.80	0.88	0.69	0.80	0.88	0.90
	ECFP6	0.57	0.78	0.80	0.89	0.66	0.79	0.89	0.89	0.61	0.80	0.81	0.91	0.68	0.80	0.91	0.91
	ESTATE	0.42	0.71	0.72	0.80	0.62	0.75	0.80	0.81	0.38	0.69	0.70	0.77	0.61	0.73	0.77	0.79
	FCFP0	0.41	0.70	0.72	0.82	0.58	0.74	0.82	0.74	0.44	0.71	0.74	0.88	0.55	0.73	0.88	0.77
	FCFP2	0.55	0.77	0.79	0.87	0.66	0.79	0.87	0.86	0.61	0.80	0.81	0.88	0.73	0.82	0.88	0.90
	FCFP4	0.56	0.77	0.79	0.87	0.68	0.80	0.87	0.88	0.55	0.77	0.79	0.88	0.65	0.78	0.88	0.88
	FCFP6	0.55	0.77	0.79	0.87	0.66	0.79	0.87	0.88	0.64	0.81	0.83	0.93	0.69	0.81	0.93	0.92
	KR	0.51	0.75	0.76	0.83	0.67	0.78	0.83	0.84	0.48	0.74	0.75	0.81	0.67	0.77	0.81	0.82
	LSTAR	0.55	0.77	0.79	0.88	0.66	0.79	0.88	0.86	0.63	0.81	0.82	0.90	0.71	0.82	0.90	0.91
	MACCS	0.56	0.78	0.79	0.84	0.71	0.81	0.84	0.86	0.55	0.78	0.78	0.80	0.76	0.82	0.80	0.86
	PUBCHEM	0.59	0.79	0.81	0.87	0.71	0.81	0.87	0.89	0.56	0.77	0.79	0.87	0.68	0.79	0.87	0.90
	RAD2D	0.56	0.77	0.79	0.89	0.66	0.79	0.89	0.87	0.56	0.77	0.80	0.92	0.62	0.78	0.92	0.87
	2PPHAR	0.12	0.56	0.58	0.33	0.79	0.57	0.33	0.62	0.11	0.55	0.58	0.28	0.82	0.57	0.28	0.63
	3PPHAR	0.33	0.67	0.66	0.72	0.62	0.61	0.72	0.72	0.32	0.66	0.66	0.73	0.60	0.60	0.73	0.71
	AP2D	0.56	0.78	0.78	0.73	0.82	0.78	0.73	0.86	0.57	0.78	0.79	0.73	0.84	0.79	0.73	0.87
	ASP	0.69	0.84	0.85	0.80	0.88	0.85	0.80	0.92	0.70	0.85	0.85	0.81	0.89	0.86	0.81	0.93
	AT2D	0.66	0.83	0.83	0.78	0.87	0.84	0.78	0.91	0.67	0.83	0.84	0.79	0.88	0.84	0.79	0.92
	DFS	0.68	0.84	0.84	0.80	0.87	0.84	0.80	0.92	0.68	0.84	0.84	0.81	0.86	0.83	0.81	0.92
	ECFP0	0.59	0.79	0.80	0.77	0.82	0.78	0.77	0.87	0.58	0.79	0.79	0.76	0.82	0.78	0.76	0.87
	ECFP2	0.68	0.84	0.84	0.82	0.87	0.83	0.82	0.92	0.70	0.85	0.85	0.83	0.87	0.84	0.83	0.92
	ECFP4	0.69	0.84	0.84	0.81	0.87	0.84	0.81	0.92	0.68	0.84	0.84	0.81	0.87	0.84	0.81	0.92
CYP1A2	ECFP6	0.68	0.84	0.84	0.80	0.87	0.84	0.80	0.92	0.67	0.83	0.84	0.79	0.87	0.84	0.79	0.92
Inhibition	ESTATE	0.58	0.79	0.79	0.76	0.82	0.78	0.76	0.87	0.59	0.80	0.80	0.75	0.84	0.79	0.75	0.88
	FCFP0	0.37	0.69	0.69	0.67	0.70	0.65	0.67	0.76	0.37	0.69	0.69	0.67	0.70	0.65	0.67	0.76
	FCFP2	0.66	0.83	0.83	0.82	0.84	0.81	0.82	0.91	0.67	0.84	0.84	0.82	0.85	0.82	0.82	0.92
	FCFP4	0.68	0.84	0.84	0.82	0.86	0.82	0.82	0.92	0.69	0.85	0.85	0.83	0.86	0.83	0.83	0.93
	FCFP6	0.68	0.84	0.84	0.80	0.87	0.84	0.80	0.92	0.68	0.84	0.84	0.80	0.87	0.84	0.80	0.92
	KR	0.66	0.83	0.83	0.80	0.86	0.82	0.80	0.91	0.66	0.83	0.83	0.81	0.85	0.82	0.81	0.91
	LSTAR	0.66	0.83	0.83	0.77	0.88	0.84	0.77	0.91	0.67	0.83	0.84	0.78	0.88	0.85	0.78	0.92
	MACCS	0.67	0.83	0.84	0.80	0.87	0.84	0.80	0.91	0.67	0.83	0.84	0.81	0.86	0.83	0.81	0.92
	PUBCHEM	0.69	0.85	0.85	0.82	0.87	0.84	0.82	0.93	0.70	0.85	0.85	0.82	0.88	0.85	0.82	0.93
	RAD2D	0.65	0.82	0.83	0.78	0.87	0.83	0.78	0.91	0.66	0.83	0.84	0.79	0.87	0.84	0.79	0.92
	2PPHAR	0.02	0.51	0.68	0.03	0.98	0.44	0.03	0.65	0.01	0.51	0.68	0.03	0.98	0.43	0.03	0.64
	3PPHAR	0.16	0.56	0.70	0.20	0.93	0.57	0.20	0.68	0.14	0.56	0.70	0.17	0.94	0.59	0.17	0.69
	AP2D	0.44	0.71	0.76	0.57	0.85	0.65	0.57	0.82	0.46	0.72	0.78	0.56	0.88	0.69	0.56	0.83
	ASP	0.57	0.78	0.82	0.66	0.89	0.74	0.66	0.88	0.58	0.78	0.82	0.66	0.90	0.76	0.66	0.89
	AT2D	0.54	0.76	0.81	0.60	0.91	0.76	0.60	0.87	0.57	0.77	0.82	0.64	0.91	0.77	0.64	0.88
	DFS	0.55	0.76	0.81	0.62	0.90	0.74	0.62	0.87	0.58	0.78	0.82	0.66	0.90	0.76	0.66	0.89
	ECFP0	0.42	0.70	0.76	0.52	0.88	0.67	0.52	0.82	0.42	0.70	0.76	0.52	0.88	0.67	0.52	0.83
	ECFP2	0.54	0.77	0.81	0.65	0.88	0.72	0.65	0.87	0.56	0.77	0.82	0.65	0.89	0.74	0.65	0.88
	ECFP4	0.55	0.77	0.81	0.64	0.89	0.74	0.64	0.87	0.58	0.78	0.82	0.66	0.90	0.76	0.66	0.88
CYP2C9	ECFP6	0.54	0.76	0.81	0.62	0.90	0.74	0.62	0.87	0.56	0.76	0.82	0.62	0.91	0.77	0.62	0.88
Inhibition	ESTATE	0.41	0.70	0.76	0.53	0.86	0.64	0.53	0.81	0.42	0.70	0.76	0.53	0.87	0.65	0.53	0.82
	FCFP0	0.26	0.62	0.71	0.35	0.89	0.59	0.35	0.75	0.27	0.62	0.72	0.35	0.89	0.60	0.35	0.75
	FCFP2	0.55	0.77	0.81	0.67	0.88	0.72	0.67	0.87	0.56	0.77	0.81	0.67	0.88	0.72	0.67	0.87
	FCFP4	0.57	0.78	0.82	0.66	0.89	0.73	0.66	0.88	0.58	0.78	0.83	0.66	0.90	0.76	0.66	0.89
	FCFP6	0.56	0.77	0.82	0.65	0.89	0.74	0.65	0.88	0.56	0.77	0.82	0.65	0.89	0.74	0.65	0.88
	KR	0.54	0.76	0.81	0.63	0.89	0.73	0.63	0.87	0.57	0.77	0.82	0.65	0.90	0.75	0.65	0.88
	LSTAR	0.53	0.75	0.81	0.58	0.91	0.76	0.58	0.87	0.56	0.76	0.82	0.60	0.92	0.78	0.60	0.89
	MACCS	0.54	0.77	0.80	0.66	0.87	0.71	0.66	0.86	0.54	0.76	0.80	0.65	0.87	0.70	0.65	0.86
	PUBCHEM	0.57	0.78	0.82	0.68	0.89	0.73	0.68	0.88	0.59	0.79	0.82	0.69	0.89	0.74	0.69	0.89
	RAD2D	0.53	0.75	0.81	0.60	0.91	0.75	0.60	0.88	0.55	0.76	0.82	0.62	0.91	0.76	0.62	0.89
	2PPHAR	0.26	0.63	0.62	0.71	0.56	0.56	0.71	0.66	0.27	0.64	0.63	0.72	0.56	0.56	0.72	0.67
	3PPHAR	0.28	0.64	0.64	0.62	0.66	0.59	0.62	0.71	0.29	0.64	0.65	0.61	0.68	0.60	0.61	0.71
	AP2D	0.51	0.75	0.76	0.73	0.78	0.72	0.73	0.83	0.52	0.76	0.76	0.74	0.78	0.72	0.74	0.84
	ASP	0.61	0.80	0.80	0.80	0.80	0.76	0.80	0.88	0.62	0.81	0.81	0.82	0.81	0.77	0.82	0.88
	AT2D	0.59	0.80	0.80	0.79	0.81	0.76	0.79	0.87	0.59	0.80	0.80	0.79	0.80	0.76	0.79	0.87
	DFS	0.59	0.80	0.80	0.81	0.79	0.75	0.81	0.87	0.60	0.80	0.80	0.82	0.78	0.75	0.82	0.87
	ECFP0	0.51	0.75	0.76	0.72	0.79	0.73	0.72	0.83	0.51	0.76	0.76	0.73	0.79	0.73	0.73	0.83
	ECFP2	0.61	0.81	0.81	0.80	0.81	0.77	0.80	0.88	0.62	0.81	0.81	0.80	0.82	0.77	0.80	0.89
	ECFP4	0.61	0.81	0.81	0.80	0.81	0.77	0.80	0.88	0.62	0.81	0.81	0.81	0.81	0.77	0.81	0.89
CYP2C19	ECFP6	0.60	0.80	0.80	0.80	0.81	0.76	0.80	0.88	0.62	0.81	0.81	0.79	0.83	0.78	0.79	0.89
Inhibition	ESTATE	0.49	0.74	0.75	0.71	0.78	0.72	0.71	0.82	0.51	0.76	0.76	0.72	0.79	0.73	0.72	0.83
	FCFP0	0.38	0.69	0.68	0.80	0.59	0.61	0.80	0.75	0.39	0.70	0.69	0.79	0.61	0.61	0.79	0.76
	FCFP2	0.59	0.80	0.80	0.79	0.80	0.76	0.79	0.87	0.60	0.80	0.80	0.79	0.81	0.76	0.79	0.87
	FCFP4	0.61	0.81	0.81	0.81	0.81	0.77	0.81	0.88	0.62	0.81	0.81	0.81	0.82	0.78	0.81	0.89
	FCFP6	0.61	0.81	0.81	0.80	0.82	0.77	0.80	0.88	0.62	0.81	0.81	0.80	0.82	0.77	0.80	0.89
	KR	0.60	0.80	0.80	0.80	0.80	0.76	0.80	0.88	0.61	0.80	0.81	0.79	0.81	0.77	0.79	0.88

Continued on next page

Table S1 – *Continued from previous page*

Endpoint	FP	Calibration												Validation			
		κ	BACC	ACC	Se	Sp	AUC	κ	BACC	ACC	Se	Sp	AUC				
	LSTAR	0.60	0.80	0.80	0.80	0.80	0.76	0.80	0.87	0.61	0.80	0.81	0.79	0.82	0.77	0.79	0.88
	MACCS	0.59	0.80	0.80	0.78	0.81	0.77	0.78	0.87	0.60	0.80	0.80	0.79	0.81	0.77	0.79	0.87
	PUBCHEM	0.60	0.80	0.80	0.79	0.81	0.77	0.79	0.87	0.59	0.80	0.80	0.78	0.81	0.77	0.78	0.88
	RAD2D	0.60	0.80	0.80	0.78	0.82	0.77	0.78	0.88	0.62	0.81	0.81	0.80	0.82	0.78	0.80	0.88
	2PPHAR	0.01	0.50	0.80	0.01	1.00	0.62	0.01	0.64	0.02	0.51	0.80	0.01	1.00	0.76	0.01	0.65
	3PPHAR	0.10	0.53	0.80	0.10	0.97	0.46	0.10	0.67	0.10	0.53	0.80	0.09	0.98	0.51	0.09	0.68
	AP2D	0.42	0.69	0.84	0.43	0.94	0.63	0.43	0.80	0.44	0.69	0.85	0.42	0.95	0.69	0.42	0.82
	ASP	0.50	0.71	0.86	0.46	0.96	0.77	0.46	0.84	0.51	0.72	0.87	0.46	0.97	0.78	0.46	0.86
	AT2D	0.47	0.69	0.86	0.41	0.97	0.78	0.41	0.83	0.50	0.71	0.87	0.44	0.97	0.81	0.44	0.86
	DFS	0.49	0.71	0.86	0.46	0.96	0.75	0.46	0.84	0.46	0.69	0.86	0.42	0.97	0.75	0.42	0.84
	ECFP0	0.39	0.68	0.82	0.43	0.92	0.58	0.43	0.78	0.40	0.67	0.84	0.37	0.96	0.69	0.37	0.81
	ECFP2	0.51	0.73	0.86	0.50	0.95	0.73	0.50	0.85	0.51	0.72	0.86	0.48	0.96	0.75	0.48	0.86
	ECFP4	0.50	0.71	0.86	0.46	0.96	0.75	0.46	0.85	0.52	0.72	0.87	0.47	0.97	0.80	0.47	0.87
CYP2D6	ECFP6	0.48	0.70	0.86	0.43	0.97	0.77	0.43	0.84	0.50	0.71	0.87	0.44	0.97	0.81	0.44	0.87
Inhibition	ESTATE	0.37	0.67	0.82	0.41	0.92	0.57	0.41	0.77	0.39	0.66	0.84	0.37	0.96	0.69	0.37	0.80
	FCFP0	0.28	0.61	0.82	0.27	0.96	0.60	0.27	0.74	0.28	0.61	0.82	0.27	0.95	0.59	0.27	0.74
	FCFP2	0.51	0.73	0.86	0.51	0.95	0.71	0.51	0.85	0.55	0.74	0.87	0.53	0.96	0.76	0.53	0.86
	FCFP4	0.53	0.73	0.87	0.51	0.96	0.76	0.51	0.86	0.54	0.73	0.88	0.49	0.97	0.80	0.49	0.87
	FCFP6	0.53	0.72	0.87	0.48	0.97	0.79	0.48	0.86	0.54	0.73	0.87	0.50	0.97	0.80	0.50	0.87
	KR	0.50	0.72	0.86	0.50	0.95	0.71	0.50	0.85	0.50	0.72	0.86	0.48	0.96	0.75	0.48	0.86
	LSTAR	0.47	0.70	0.86	0.44	0.96	0.74	0.44	0.84	0.48	0.70	0.86	0.42	0.97	0.79	0.42	0.85
	MACCS	0.51	0.72	0.86	0.48	0.96	0.74	0.48	0.85	0.52	0.73	0.87	0.49	0.96	0.75	0.49	0.87
	PUBCHEM	0.51	0.72	0.86	0.48	0.96	0.75	0.48	0.86	0.52	0.73	0.87	0.49	0.96	0.76	0.49	0.87
	RAD2D	0.50	0.71	0.87	0.46	0.97	0.78	0.46	0.85	0.53	0.72	0.87	0.47	0.97	0.81	0.47	0.88
	2PPHAR	0.29	0.65	0.64	0.67	0.63	0.55	0.67	0.70	0.27	0.64	0.64	0.67	0.61	0.54	0.67	0.69
	3PPHAR	0.31	0.66	0.67	0.59	0.72	0.59	0.59	0.73	0.31	0.65	0.67	0.59	0.72	0.59	0.59	0.73
	AP2D	0.47	0.73	0.75	0.66	0.81	0.70	0.66	0.82	0.49	0.74	0.76	0.68	0.81	0.71	0.68	0.84
	ASP	0.60	0.80	0.81	0.75	0.84	0.77	0.75	0.89	0.62	0.80	0.82	0.73	0.88	0.80	0.73	0.90
	AT2D	0.58	0.79	0.80	0.70	0.87	0.79	0.70	0.88	0.59	0.79	0.81	0.69	0.88	0.80	0.69	0.88
	DFS	0.60	0.80	0.81	0.73	0.86	0.78	0.73	0.89	0.61	0.80	0.81	0.75	0.86	0.78	0.75	0.89
	ECFP0	0.44	0.72	0.73	0.62	0.81	0.69	0.62	0.81	0.43	0.71	0.73	0.60	0.81	0.69	0.60	0.81
	ECFP2	0.59	0.79	0.80	0.72	0.86	0.78	0.72	0.89	0.59	0.79	0.81	0.70	0.88	0.80	0.70	0.89
	ECFP4	0.60	0.80	0.81	0.73	0.87	0.79	0.73	0.89	0.61	0.80	0.82	0.70	0.90	0.82	0.70	0.90
CYP3A4	ECFP6	0.59	0.79	0.81	0.73	0.86	0.78	0.73	0.89	0.59	0.79	0.80	0.69	0.88	0.80	0.69	0.89
Inhibition	ESTATE	0.43	0.71	0.73	0.62	0.80	0.68	0.62	0.80	0.43	0.71	0.73	0.63	0.80	0.68	0.63	0.80
	FCFP0	0.32	0.67	0.65	0.77	0.57	0.55	0.77	0.72	0.32	0.67	0.65	0.81	0.53	0.54	0.81	0.71
	FCFP2	0.55	0.77	0.79	0.71	0.84	0.75	0.71	0.87	0.56	0.77	0.79	0.70	0.85	0.76	0.70	0.87
	FCFP4	0.59	0.79	0.80	0.73	0.85	0.77	0.73	0.89	0.62	0.81	0.82	0.74	0.88	0.80	0.74	0.90
	FCFP6	0.60	0.80	0.81	0.74	0.86	0.78	0.74	0.89	0.60	0.80	0.81	0.74	0.86	0.78	0.74	0.90
	KR	0.57	0.78	0.80	0.71	0.85	0.77	0.71	0.88	0.56	0.77	0.79	0.68	0.87	0.78	0.68	0.88
	LSTAR	0.59	0.79	0.80	0.74	0.85	0.77	0.74	0.88	0.60	0.80	0.81	0.72	0.87	0.80	0.72	0.89
	MACCS	0.54	0.77	0.78	0.70	0.84	0.75	0.70	0.87	0.57	0.78	0.79	0.71	0.85	0.76	0.71	0.87
	PUBCHEM	0.59	0.79	0.80	0.74	0.84	0.76	0.74	0.89	0.60	0.80	0.81	0.75	0.85	0.77	0.75	0.89
	RAD2D	0.58	0.78	0.80	0.69	0.88	0.79	0.69	0.89	0.58	0.78	0.80	0.67	0.89	0.81	0.67	0.89
	2PPHAR	0.10	0.55	0.72	0.83	0.27	0.83	0.83	0.57	0.06	0.53	0.73	0.85	0.21	0.82	0.85	0.58
	3PPHAR	0.15	0.56	0.77	0.90	0.22	0.83	0.90	0.61	0.12	0.55	0.77	0.90	0.20	0.83	0.90	0.61
	AP2D	0.25	0.64	0.74	0.80	0.49	0.87	0.80	0.71	0.27	0.64	0.76	0.83	0.46	0.87	0.83	0.72
	ASP	0.31	0.66	0.78	0.86	0.46	0.87	0.86	0.72	0.28	0.62	0.80	0.91	0.33	0.86	0.91	0.75
	AT2D	0.32	0.66	0.79	0.86	0.46	0.87	0.86	0.73	0.32	0.67	0.78	0.85	0.48	0.88	0.85	0.75
	DFS	0.30	0.66	0.78	0.85	0.47	0.87	0.85	0.72	0.35	0.65	0.83	0.94	0.37	0.87	0.94	0.73
	ECFP0	0.20	0.62	0.72	0.78	0.45	0.86	0.78	0.67	0.15	0.57	0.78	0.91	0.23	0.84	0.91	0.67
	ECFP2	0.27	0.64	0.77	0.85	0.42	0.87	0.85	0.70	0.29	0.64	0.79	0.88	0.39	0.86	0.88	0.75
	ECFP4	0.28	0.65	0.76	0.83	0.48	0.87	0.83	0.71	0.27	0.62	0.80	0.90	0.35	0.86	0.90	0.71
Drug Induced	ECFP6	0.32	0.64	0.81	0.92	0.37	0.86	0.92	0.71	0.35	0.65	0.83	0.93	0.37	0.86	0.93	0.72
Choleostasis	ESTATE	0.20	0.62	0.71	0.76	0.49	0.86	0.76	0.68	0.15	0.59	0.69	0.75	0.43	0.85	0.75	0.66
	FCFP0	0.10	0.56	0.68	0.75	0.36	0.84	0.75	0.59	0.03	0.52	0.69	0.79	0.25	0.82	0.79	0.57
	FCFP2	0.29	0.65	0.79	0.87	0.42	0.87	0.87	0.71	0.29	0.64	0.80	0.89	0.38	0.86	0.89	0.68
	FCFP4	0.31	0.65	0.79	0.87	0.44	0.87	0.87	0.72	0.29	0.63	0.80	0.91	0.36	0.86	0.91	0.73
	FCFP6	0.30	0.65	0.78	0.86	0.44	0.87	0.86	0.70	0.38	0.67	0.83	0.92	0.43	0.87	0.92	0.77
	KR	0.33	0.67	0.79	0.87	0.46	0.87	0.87	0.73	0.32	0.65	0.80	0.89	0.41	0.87	0.89	0.73
	LSTAR	0.26	0.64	0.76	0.83	0.45	0.87	0.83	0.70	0.29	0.62	0.82	0.93	0.31	0.85	0.93	0.70
	MACCS	0.29	0.66	0.77	0.84	0.48	0.87	0.84	0.72	0.28	0.64	0.77	0.85	0.44	0.87	0.85	0.71
	PUBCHEM	0.29	0.65	0.78	0.86	0.43	0.87	0.86	0.73	0.32	0.66	0.80	0.89	0.43	0.87	0.89	0.74
	RAD2D	0.33	0.67	0.79	0.86	0.48	0.88	0.86	0.73	0.36	0.66	0.82	0.91	0.42	0.87	0.91	0.74
	2PPHAR	0.13	0.59	0.76	0.81	0.38	0.91	0.81	0.60	0.19	0.64	0.78	0.81	0.47	0.93	0.81	0.67
	3PPHAR	0.18	0.60	0.83	0.89	0.31	0.91	0.89	0.67	0.18	0.60	0.83	0.90	0.30	0.91	0.90	0.70
	AP2D	0.27	0.68	0.80	0.84	0.53	0.94	0.84	0.76	0.23	0.63	0.84	0.89	0.36	0.92	0.89	0.77
	ASP	0.31	0.67	0.85	0.90	0.44	0.93	0.90	0.75	0.26	0.63	0.86	0.93	0.33	0.92	0.93	0.75
	AT2D	0.28	0.66	0.84	0.89	0.42	0.93	0.89	0.75	0.31	0.70	0.84	0.88	0.51	0.94	0.88	0.79

Continued on next page

Drug Induced
Rhabdomyolysis

Table S1 – *Continued from previous page*

Endpoint	FP	Calibration											Validation				
		κ	BACC	ACC	Se	Sp	AUC	κ	BACC	ACC	Se	Sp	AUC				
	DFS	0.26	0.65	0.83	0.88	0.41	0.92	0.88	0.72	0.23	0.62	0.85	0.91	0.33	0.92	0.91	0.73
	ECFP0	0.23	0.69	0.75	0.77	0.62	0.94	0.77	0.75	0.19	0.66	0.73	0.75	0.57	0.94	0.75	0.74
	ECFP2	0.28	0.68	0.82	0.86	0.50	0.93	0.86	0.77	0.25	0.66	0.82	0.86	0.46	0.93	0.86	0.77
	ECFP4	0.26	0.64	0.84	0.89	0.40	0.92	0.89	0.74	0.32	0.67	0.86	0.91	0.44	0.93	0.91	0.80
	ECFP6	0.23	0.66	0.80	0.84	0.48	0.93	0.84	0.71	0.14	0.58	0.83	0.90	0.26	0.91	0.90	0.71
	ESTATE	0.21	0.68	0.74	0.75	0.61	0.94	0.75	0.75	0.20	0.68	0.73	0.75	0.60	0.94	0.75	0.74
	FCFP0	0.20	0.63	0.80	0.84	0.42	0.92	0.84	0.69	0.16	0.60	0.80	0.85	0.34	0.92	0.85	0.69
	FCFP2	0.28	0.69	0.81	0.85	0.53	0.94	0.85	0.76	0.24	0.66	0.81	0.85	0.47	0.93	0.85	0.76
	FCFP4	0.30	0.68	0.84	0.88	0.48	0.93	0.88	0.77	0.26	0.64	0.85	0.90	0.39	0.92	0.90	0.74
	FCFP6	0.25	0.66	0.81	0.86	0.46	0.93	0.86	0.74	0.29	0.67	0.84	0.88	0.46	0.93	0.88	0.75
	KR	0.28	0.67	0.83	0.88	0.46	0.93	0.88	0.77	0.31	0.69	0.84	0.88	0.50	0.94	0.88	0.78
	LSTAR	0.17	0.59	0.81	0.88	0.31	0.91	0.88	0.66	0.29	0.63	0.87	0.94	0.32	0.92	0.94	0.76
	MACCS	0.32	0.71	0.82	0.85	0.58	0.94	0.85	0.80	0.27	0.70	0.81	0.84	0.55	0.94	0.84	0.83
	PUBCHEM	0.29	0.67	0.83	0.88	0.47	0.93	0.88	0.76	0.35	0.72	0.85	0.89	0.54	0.94	0.89	0.82
	RAD2D	0.25	0.63	0.85	0.91	0.34	0.92	0.91	0.72	0.34	0.66	0.88	0.94	0.38	0.93	0.94	0.78
Drug Induced Liver Injury	2PPHAR	0.04	0.52	0.62	0.11	0.93	0.50	0.11	0.55	0.05	0.52	0.62	0.09	0.95	0.52	0.09	0.55
	3PPHAR	0.19	0.58	0.66	0.26	0.91	0.65	0.26	0.68	0.16	0.57	0.65	0.24	0.91	0.60	0.24	0.68
	AP2D	0.50	0.75	0.77	0.66	0.83	0.71	0.66	0.84	0.46	0.73	0.75	0.65	0.81	0.68	0.65	0.82
	ASP	0.57	0.78	0.80	0.74	0.83	0.73	0.74	0.87	0.57	0.78	0.80	0.70	0.86	0.75	0.70	0.87
	AT2D	0.55	0.78	0.79	0.74	0.81	0.71	0.74	0.86	0.64	0.82	0.83	0.79	0.86	0.77	0.79	0.89
	DFS	0.54	0.77	0.78	0.73	0.82	0.71	0.73	0.86	0.57	0.78	0.80	0.70	0.86	0.75	0.70	0.86
	ECFP0	0.45	0.73	0.74	0.70	0.76	0.64	0.70	0.81	0.52	0.76	0.78	0.71	0.81	0.70	0.71	0.84
	ECFP2	0.58	0.78	0.81	0.70	0.87	0.77	0.70	0.86	0.57	0.78	0.80	0.68	0.88	0.77	0.68	0.87
	ECFP4	0.54	0.77	0.79	0.69	0.84	0.73	0.69	0.85	0.59	0.79	0.81	0.69	0.89	0.79	0.69	0.86
	ECFP6	0.49	0.74	0.77	0.61	0.87	0.74	0.61	0.82	0.54	0.76	0.79	0.63	0.89	0.77	0.63	0.84
	ESTATE	0.42	0.71	0.73	0.63	0.78	0.64	0.63	0.79	0.43	0.72	0.73	0.64	0.79	0.65	0.64	0.80
	FCFP0	0.20	0.60	0.64	0.43	0.76	0.53	0.43	0.64	0.19	0.59	0.64	0.37	0.81	0.54	0.37	0.63
	FCFP2	0.53	0.76	0.78	0.69	0.83	0.72	0.69	0.84	0.58	0.79	0.81	0.71	0.86	0.76	0.71	0.87
	FCFP4	0.54	0.77	0.79	0.67	0.86	0.75	0.67	0.85	0.59	0.79	0.81	0.70	0.88	0.77	0.70	0.88
	FCFP6	0.53	0.76	0.78	0.70	0.84	0.72	0.70	0.84	0.59	0.78	0.81	0.66	0.91	0.81	0.66	0.87
	KR	0.56	0.78	0.80	0.68	0.87	0.76	0.68	0.86	0.56	0.78	0.80	0.69	0.86	0.76	0.69	0.87
	LSTAR	0.53	0.76	0.78	0.69	0.84	0.73	0.69	0.85	0.58	0.78	0.81	0.64	0.92	0.83	0.64	0.87
	MACCS	0.55	0.77	0.79	0.73	0.82	0.71	0.73	0.86	0.63	0.82	0.82	0.79	0.84	0.76	0.79	0.89
	PUBCHEM	0.57	0.78	0.80	0.71	0.86	0.75	0.71	0.86	0.58	0.79	0.81	0.69	0.88	0.78	0.69	0.88
	RAD2D	0.56	0.77	0.80	0.66	0.88	0.77	0.66	0.86	0.59	0.79	0.81	0.68	0.89	0.79	0.68	0.88
Hepatic Steatosis	2PPHAR	0.17	0.61	0.77	0.81	0.40	0.91	0.81	0.64	0.09	0.55	0.79	0.86	0.25	0.90	0.86	0.59
	3PPHAR	0.05	0.54	0.80	0.88	0.19	0.89	0.88	0.54	-0.06	0.47	0.81	0.91	0.03	0.88	0.91	0.38
	AP2D	0.13	0.58	0.77	0.82	0.34	0.90	0.82	0.60	0.19	0.62	0.78	0.83	0.42	0.92	0.83	0.76
	ASP	0.17	0.59	0.81	0.88	0.30	0.90	0.88	0.63	0.21	0.60	0.83	0.90	0.31	0.91	0.90	0.66
	AT2D	0.19	0.61	0.79	0.84	0.39	0.91	0.84	0.65	0.18	0.60	0.80	0.86	0.33	0.91	0.86	0.58
	DFS	0.14	0.60	0.74	0.79	0.42	0.91	0.79	0.62	0.13	0.57	0.80	0.87	0.28	0.90	0.87	0.62
	ECFP0	0.17	0.62	0.74	0.78	0.47	0.92	0.78	0.67	0.16	0.56	0.86	0.95	0.17	0.89	0.95	0.59
	ECFP2	0.20	0.62	0.81	0.87	0.36	0.91	0.87	0.66	0.20	0.60	0.81	0.87	0.33	0.91	0.87	0.63
	ECFP4	0.15	0.58	0.81	0.88	0.28	0.90	0.88	0.63	0.11	0.55	0.81	0.90	0.19	0.89	0.90	0.57
	ECFP6	0.12	0.57	0.78	0.84	0.30	0.90	0.84	0.62	0.22	0.61	0.85	0.92	0.31	0.91	0.92	0.64
	ESTATE	0.14	0.61	0.72	0.75	0.47	0.91	0.75	0.65	0.11	0.59	0.70	0.73	0.44	0.91	0.73	0.62
	FCFP0	0.09	0.58	0.69	0.73	0.43	0.91	0.73	0.60	0.01	0.51	0.70	0.75	0.28	0.89	0.75	0.62
	FCFP2	0.16	0.60	0.78	0.83	0.38	0.91	0.83	0.66	0.07	0.55	0.80	0.87	0.22	0.89	0.87	0.58
	FCFP4	0.18	0.61	0.80	0.86	0.36	0.91	0.86	0.66	0.15	0.58	0.82	0.90	0.25	0.90	0.90	0.60
	FCFP6	0.15	0.58	0.81	0.89	0.28	0.90	0.89	0.61	0.14	0.58	0.80	0.87	0.28	0.90	0.87	0.66
	KR	0.15	0.58	0.81	0.88	0.29	0.90	0.88	0.62	0.15	0.57	0.82	0.90	0.25	0.90	0.90	0.70
	LSTAR	0.16	0.58	0.87	0.95	0.20	0.90	0.95	0.59	0.05	0.52	0.83	0.93	0.11	0.89	0.93	0.61
	MACCS	0.25	0.63	0.84	0.90	0.35	0.91	0.90	0.67	0.18	0.59	0.83	0.91	0.28	0.90	0.91	0.68
	PUBCHEM	0.18	0.60	0.81	0.87	0.33	0.91	0.87	0.63	0.13	0.55	0.83	0.91	0.19	0.89	0.91	0.65
	RAD2D	0.18	0.58	0.86	0.94	0.21	0.90	0.94	0.64	0.17	0.57	0.86	0.94	0.19	0.90	0.94	0.61
Human Intestinal Absorption	2PPHAR	0.37	0.70	0.81	0.53	0.86	0.46	0.53	0.75	0.41	0.71	0.82	0.55	0.87	0.49	0.55	0.78
	3PPHAR	0.38	0.70	0.80	0.55	0.86	0.45	0.55	0.76	0.43	0.73	0.83	0.57	0.89	0.51	0.57	0.78
	AP2D	0.49	0.75	0.85	0.61	0.90	0.57	0.61	0.83	0.54	0.77	0.87	0.62	0.92	0.62	0.62	0.84
	ASP	0.62	0.82	0.89	0.71	0.93	0.67	0.71	0.88	0.64	0.80	0.90	0.65	0.95	0.75	0.65	0.88
	AT2D	0.60	0.81	0.88	0.71	0.91	0.64	0.71	0.87	0.62	0.81	0.89	0.70	0.93	0.67	0.70	0.86
	DFS	0.59	0.79	0.88	0.65	0.93	0.68	0.65	0.86	0.66	0.82	0.90	0.70	0.95	0.74	0.70	0.88
	ECFP0	0.55	0.81	0.85	0.75	0.87	0.57	0.75	0.87	0.66	0.87	0.89	0.83	0.90	0.64	0.83	0.92
	ECFP2	0.63	0.83	0.89	0.74	0.92	0.67	0.74	0.90	0.65	0.82	0.90	0.70	0.94	0.72	0.70	0.89
	ECFP4	0.57	0.79	0.87	0.68	0.91	0.63	0.68	0.86	0.67	0.84	0.90	0.74	0.94	0.72	0.74	0.91
	ECFP6	0.56	0.78	0.87	0.63	0.93	0.66	0.63	0.86	0.59	0.79	0.88	0.65	0.93	0.67	0.65	0.89
	ESTATE	0.56	0.81	0.86	0.74	0.88	0.58	0.74	0.87	0.52	0.79	0.84	0.70	0.88	0.54	0.70	0.86
	FCFP0	0.31	0.68	0.76	0.56	0.80	0.38	0.56	0.74	0.28	0.66	0.76	0.52	0.81	0.37	0.52	0.71
	FCFP2	0.60	0.81	0.88	0.71	0.92	0.65	0.71	0.88	0.62	0.81	0.89	0.68	0.94	0.70	0.68	0.89
	FCFP4	0.59	0.80	0.88	0.69	0.92	0.65	0.69	0.88	0.62	0.82	0.88	0.72	0.92	0.66	0.72	0.88

Continued on next page

Table S1 – *Continued from previous page*

Endpoint	FP	Calibration										Validation					
		κ	BACC	ACC	Se	Sp	AUC	κ	BACC	ACC	Se	Sp	AUC				
	FCFP6	0.57	0.79	0.87	0.66	0.92	0.64	0.66	0.86	0.60	0.79	0.89	0.63	0.95	0.72	0.63	0.88
	KR	0.57	0.78	0.88	0.63	0.93	0.67	0.63	0.87	0.63	0.81	0.89	0.68	0.94	0.70	0.68	0.88
	LSTAR	0.59	0.81	0.87	0.71	0.91	0.63	0.71	0.87	0.56	0.77	0.88	0.60	0.94	0.66	0.60	0.85
	MACCS	0.64	0.84	0.89	0.77	0.91	0.66	0.77	0.89	0.63	0.83	0.89	0.74	0.92	0.66	0.74	0.89
	PUBCHEM	0.58	0.80	0.87	0.68	0.91	0.63	0.68	0.87	0.60	0.81	0.88	0.70	0.92	0.65	0.70	0.87
	RAD2D	0.62	0.83	0.88	0.74	0.92	0.66	0.74	0.89	0.62	0.80	0.89	0.67	0.94	0.70	0.67	0.86
Human Liver Microsomal Stability	2PPHAR	0.01	0.50	0.62	0.94	0.07	0.63	0.94	0.51	0.00	0.50	0.62	0.95	0.05	0.63	0.95	0.50
	3PPHAR	0.22	0.60	0.68	0.92	0.27	0.69	0.92	0.69	0.19	0.58	0.67	0.93	0.24	0.68	0.93	0.68
	AP2D	0.42	0.70	0.73	0.82	0.59	0.78	0.82	0.78	0.44	0.71	0.75	0.84	0.59	0.78	0.84	0.80
	ASP	0.52	0.75	0.78	0.88	0.63	0.80	0.88	0.83	0.54	0.76	0.79	0.87	0.65	0.81	0.87	0.85
	AT2D	0.55	0.77	0.79	0.84	0.71	0.83	0.84	0.83	0.53	0.77	0.78	0.84	0.69	0.83	0.84	0.84
	DFS	0.52	0.75	0.78	0.86	0.64	0.81	0.86	0.82	0.55	0.77	0.79	0.87	0.67	0.82	0.87	0.85
	ECFP0	0.42	0.71	0.73	0.79	0.63	0.78	0.79	0.78	0.45	0.72	0.74	0.80	0.64	0.80	0.80	0.80
	ECFP2	0.53	0.76	0.79	0.88	0.63	0.80	0.88	0.84	0.57	0.78	0.81	0.90	0.65	0.82	0.90	0.87
	ECFP4	0.52	0.76	0.79	0.87	0.64	0.81	0.87	0.84	0.54	0.76	0.79	0.87	0.65	0.81	0.87	0.86
	ECFP6	0.51	0.75	0.78	0.89	0.60	0.79	0.89	0.83	0.52	0.75	0.79	0.91	0.59	0.79	0.91	0.85
	ESTATE	0.40	0.70	0.72	0.78	0.62	0.78	0.78	0.77	0.42	0.71	0.73	0.78	0.64	0.79	0.78	0.78
	FCFP0	0.13	0.56	0.62	0.77	0.35	0.67	0.77	0.64	0.11	0.55	0.62	0.82	0.28	0.66	0.82	0.64
	FCFP2	0.52	0.75	0.78	0.86	0.65	0.81	0.86	0.83	0.55	0.77	0.80	0.88	0.65	0.81	0.88	0.85
	FCFP4	0.54	0.76	0.80	0.88	0.64	0.81	0.88	0.84	0.55	0.77	0.80	0.88	0.65	0.81	0.88	0.85
	FCFP6	0.51	0.75	0.78	0.88	0.61	0.80	0.88	0.84	0.52	0.75	0.79	0.91	0.58	0.79	0.91	0.85
	KR	0.51	0.75	0.78	0.87	0.63	0.80	0.87	0.83	0.46	0.72	0.76	0.87	0.57	0.78	0.87	0.83
	LSTAR	0.51	0.74	0.79	0.92	0.56	0.78	0.92	0.83	0.48	0.72	0.77	0.91	0.54	0.77	0.91	0.82
	MACCS	0.52	0.76	0.78	0.84	0.67	0.82	0.84	0.83	0.52	0.76	0.78	0.84	0.67	0.82	0.84	0.84
	PUBCHEM	0.51	0.75	0.78	0.85	0.64	0.80	0.85	0.83	0.50	0.74	0.77	0.86	0.63	0.80	0.86	0.83
	RAD2D	0.54	0.76	0.79	0.90	0.61	0.80	0.90	0.84	0.50	0.74	0.78	0.91	0.56	0.78	0.91	0.84
Breast cancer resistance protein inhibition	2PPHAR	0.18	0.61	0.59	0.66	0.55	0.39	0.66	0.64	0.06	0.54	0.59	0.31	0.77	0.21	0.31	0.63
	3PPHAR	0.33	0.65	0.70	0.41	0.89	0.71	0.41	0.80	0.33	0.65	0.70	0.43	0.87	0.69	0.43	0.79
	AP2D	0.62	0.81	0.82	0.80	0.83	0.76	0.80	0.89	0.65	0.83	0.83	0.81	0.85	0.77	0.81	0.90
	ASP	0.77	0.88	0.89	0.84	0.93	0.88	0.84	0.94	0.77	0.88	0.89	0.83	0.93	0.89	0.83	0.95
	AT2D	0.76	0.88	0.88	0.86	0.90	0.85	0.86	0.94	0.77	0.88	0.89	0.85	0.92	0.87	0.85	0.94
	DFS	0.76	0.88	0.89	0.84	0.92	0.87	0.84	0.93	0.79	0.89	0.90	0.84	0.93	0.89	0.84	0.95
	ECFP0	0.59	0.80	0.80	0.81	0.79	0.72	0.81	0.88	0.58	0.80	0.80	0.80	0.79	0.71	0.80	0.88
	ECFP2	0.75	0.87	0.88	0.85	0.90	0.85	0.85	0.94	0.73	0.86	0.87	0.81	0.91	0.86	0.81	0.95
	ECFP4	0.76	0.88	0.89	0.84	0.92	0.87	0.84	0.94	0.74	0.86	0.88	0.81	0.92	0.87	0.81	0.93
	ECFP6	0.77	0.88	0.89	0.82	0.94	0.89	0.82	0.94	0.78	0.88	0.90	0.82	0.95	0.91	0.82	0.95
	ESTATE	0.60	0.81	0.80	0.81	0.80	0.72	0.81	0.88	0.59	0.80	0.80	0.82	0.78	0.71	0.82	0.88
	FCFP0	0.16	0.58	0.62	0.38	0.77	0.52	0.38	0.62	0.12	0.56	0.60	0.36	0.76	0.49	0.36	0.58
	FCFP2	0.70	0.85	0.86	0.83	0.88	0.81	0.83	0.92	0.71	0.85	0.86	0.80	0.90	0.84	0.80	0.94
	FCFP4	0.78	0.89	0.89	0.85	0.92	0.87	0.85	0.95	0.81	0.90	0.91	0.88	0.93	0.89	0.88	0.96
	FCFP6	0.78	0.88	0.89	0.84	0.93	0.88	0.84	0.94	0.78	0.88	0.90	0.83	0.94	0.90	0.83	0.94
	KR	0.72	0.86	0.87	0.81	0.91	0.85	0.81	0.93	0.77	0.88	0.89	0.84	0.92	0.87	0.84	0.94
	LSTAR	0.74	0.86	0.88	0.79	0.93	0.89	0.79	0.93	0.76	0.87	0.89	0.80	0.94	0.90	0.80	0.93
	MACCS	0.69	0.85	0.85	0.83	0.86	0.80	0.83	0.93	0.71	0.86	0.86	0.84	0.88	0.82	0.84	0.94
	PUBCHEM	0.75	0.87	0.88	0.85	0.90	0.85	0.85	0.94	0.76	0.88	0.89	0.83	0.92	0.87	0.83	0.94
	RAD2D	0.76	0.88	0.89	0.83	0.92	0.87	0.83	0.94	0.78	0.89	0.90	0.86	0.93	0.88	0.86	0.95
Blood Brain Barrier Permeability	2PPHAR	0.31	0.64	0.76	0.42	0.87	0.52	0.42	0.70	0.30	0.63	0.77	0.38	0.89	0.52	0.38	0.70
	3PPHAR	0.32	0.64	0.78	0.39	0.90	0.56	0.39	0.76	0.32	0.64	0.77	0.39	0.90	0.55	0.39	0.76
	AP2D	0.64	0.81	0.87	0.69	0.93	0.76	0.69	0.90	0.64	0.81	0.88	0.68	0.94	0.78	0.68	0.91
	ASP	0.66	0.83	0.88	0.74	0.92	0.74	0.74	0.90	0.66	0.83	0.88	0.73	0.92	0.76	0.73	0.90
	AT2D	0.61	0.81	0.85	0.74	0.89	0.68	0.74	0.89	0.62	0.81	0.86	0.71	0.91	0.71	0.71	0.89
	DFS	0.63	0.82	0.86	0.75	0.90	0.70	0.75	0.89	0.67	0.82	0.88	0.71	0.94	0.79	0.71	0.90
	ECFP0	0.60	0.81	0.85	0.75	0.88	0.66	0.75	0.89	0.61	0.81	0.86	0.71	0.90	0.70	0.71	0.90
	ECFP2	0.65	0.82	0.87	0.73	0.92	0.74	0.73	0.91	0.65	0.82	0.87	0.73	0.92	0.74	0.73	0.92
	ECFP4	0.65	0.81	0.88	0.69	0.93	0.76	0.69	0.89	0.67	0.80	0.89	0.64	0.97	0.87	0.64	0.92
	ECFP6	0.62	0.80	0.87	0.67	0.93	0.76	0.67	0.88	0.66	0.80	0.89	0.63	0.97	0.87	0.63	0.92
	ESTATE	0.60	0.81	0.85	0.73	0.89	0.68	0.73	0.89	0.57	0.78	0.84	0.67	0.90	0.67	0.67	0.87
	FCFP0	0.37	0.70	0.75	0.60	0.80	0.49	0.60	0.78	0.36	0.69	0.76	0.56	0.82	0.50	0.56	0.76
	FCFP2	0.64	0.82	0.87	0.73	0.91	0.73	0.73	0.91	0.64	0.81	0.87	0.69	0.93	0.76	0.69	0.90
	FCFP4	0.66	0.83	0.88	0.74	0.92	0.74	0.74	0.91	0.67	0.84	0.88	0.76	0.92	0.74	0.76	0.93
	FCFP6	0.66	0.81	0.88	0.68	0.95	0.80	0.68	0.90	0.71	0.84	0.90	0.73	0.95	0.83	0.73	0.93
	KR	0.64	0.82	0.87	0.73	0.91	0.73	0.73	0.91	0.65	0.83	0.87	0.74	0.91	0.73	0.74	0.92
	LSTAR	0.64	0.82	0.86	0.74	0.91	0.71	0.74	0.88	0.62	0.81	0.86	0.70	0.91	0.72	0.70	0.88
	MACCS	0.66	0.83	0.87	0.75	0.91	0.73	0.75	0.91	0.64	0.81	0.87	0.69	0.93	0.77	0.69	0.92
	PUBCHEM	0.66	0.82	0.88	0.71	0.93	0.76	0.71	0.90	0.64	0.81	0.87	0.69	0.93	0.76	0.69	0.92
	RAD2D	0.66	0.82	0.88	0.72	0.93	0.76	0.72	0.91	0.62	0.80	0.86	0.69	0.92	0.73	0.69	0.90
Phosphate buffer Solubility	2PPHAR	0.19	0.62	0.58	0.52	0.71	0.81	0.52	0.65	0.18	0.61	0.58	0.53	0.70	0.80	0.53	0.65
	3PPHAR	0.18	0.60	0.59	0.57	0.64	0.78	0.57	0.64	0.17	0.60	0.59	0.56	0.65	0.78	0.56	0.65
	AP2D	0.32	0.68	0.67	0.66	0.70	0.83	0.66	0.74	0.34	0.69	0.68	0.67	0.71	0.84	0.67	0.75

Continued on next page

Table S1 – *Continued from previous page*

Endpoint	FP	Calibration						Validation									
		κ	BACC	ACC	Se	Sp	AUC	κ	BACC	ACC	Se	Sp	AUC				
	ASP	0.51	0.78	0.77	0.77	0.78	0.89	0.77	0.85	0.52	0.78	0.78	0.78	0.78	0.89	0.78	0.86
	AT2D	0.48	0.76	0.76	0.77	0.76	0.88	0.77	0.84	0.50	0.77	0.77	0.77	0.76	0.88	0.77	0.84
	DFS	0.49	0.77	0.76	0.75	0.78	0.89	0.75	0.84	0.50	0.77	0.77	0.76	0.79	0.89	0.76	0.85
	ECFP0	0.27	0.65	0.65	0.64	0.66	0.81	0.64	0.70	0.29	0.66	0.66	0.65	0.68	0.82	0.65	0.72
	ECFP2	0.50	0.78	0.77	0.76	0.79	0.89	0.76	0.86	0.52	0.78	0.78	0.76	0.81	0.90	0.76	0.87
	ECFP4	0.51	0.78	0.77	0.76	0.79	0.90	0.76	0.86	0.52	0.78	0.78	0.77	0.80	0.90	0.77	0.87
	ECFP6	0.50	0.77	0.77	0.76	0.78	0.89	0.76	0.85	0.51	0.78	0.77	0.77	0.79	0.90	0.77	0.86
	ESTATE	0.31	0.67	0.68	0.68	0.67	0.82	0.68	0.73	0.33	0.68	0.68	0.69	0.68	0.83	0.69	0.74
	FCFP0	0.16	0.61	0.50	0.33	0.89	0.88	0.33	0.64	0.16	0.61	0.52	0.39	0.83	0.84	0.39	0.64
	FCFP2	0.46	0.76	0.74	0.72	0.79	0.89	0.72	0.84	0.45	0.75	0.74	0.71	0.80	0.89	0.71	0.84
	FCFP4	0.50	0.78	0.77	0.76	0.80	0.90	0.76	0.86	0.51	0.78	0.78	0.77	0.79	0.90	0.77	0.86
	FCFP6	0.50	0.77	0.76	0.75	0.80	0.89	0.75	0.85	0.52	0.78	0.78	0.76	0.80	0.90	0.76	0.86
	KR	0.47	0.76	0.75	0.74	0.79	0.89	0.74	0.84	0.48	0.76	0.75	0.74	0.79	0.89	0.74	0.84
	LSTAR	0.48	0.76	0.76	0.74	0.78	0.89	0.74	0.84	0.49	0.77	0.77	0.76	0.78	0.89	0.76	0.85
	MACCS	0.49	0.77	0.76	0.76	0.78	0.89	0.76	0.85	0.50	0.77	0.77	0.77	0.78	0.89	0.77	0.86
	PUBCHEM	0.53	0.79	0.78	0.78	0.80	0.90	0.78	0.87	0.54	0.79	0.79	0.79	0.80	0.90	0.79	0.87
	RAD2D	0.49	0.77	0.76	0.75	0.78	0.89	0.75	0.85	0.50	0.77	0.77	0.76	0.78	0.89	0.76	0.85
	2PPHAR	0.02	0.55	0.46	0.66	0.45	0.06	0.66	0.57	0.02	0.55	0.45	0.65	0.44	0.06	0.65	0.56
	3PPHAR	0.03	0.56	0.55	0.57	0.55	0.07	0.57	0.58	0.02	0.55	0.58	0.51	0.59	0.06	0.51	0.57
	AP2D	0.07	0.63	0.63	0.64	0.63	0.09	0.64	0.68	0.07	0.64	0.63	0.65	0.62	0.09	0.65	0.69
	ASP	0.14	0.71	0.74	0.68	0.74	0.13	0.68	0.78	0.14	0.70	0.76	0.64	0.76	0.13	0.64	0.77
	AT2D	0.12	0.70	0.71	0.69	0.71	0.12	0.69	0.76	0.14	0.72	0.73	0.70	0.73	0.13	0.70	0.78
	DFS	0.13	0.72	0.71	0.72	0.71	0.12	0.72	0.77	0.14	0.71	0.74	0.67	0.75	0.13	0.67	0.77
	ECFP0	0.09	0.66	0.66	0.66	0.66	0.10	0.66	0.72	0.09	0.66	0.67	0.65	0.67	0.10	0.65	0.72
	ECFP2	0.14	0.72	0.72	0.71	0.72	0.12	0.71	0.78	0.15	0.73	0.73	0.74	0.73	0.13	0.74	0.80
	ECFP4	0.14	0.72	0.74	0.70	0.74	0.13	0.70	0.78	0.14	0.71	0.74	0.68	0.74	0.13	0.68	0.78
	ECFP6	0.13	0.72	0.72	0.72	0.72	0.12	0.72	0.78	0.15	0.73	0.75	0.71	0.75	0.14	0.71	0.80
DMSO Solubility	ESTATE	0.09	0.67	0.66	0.67	0.66	0.10	0.67	0.72	0.09	0.67	0.67	0.68	0.67	0.10	0.68	0.73
	FCFP0	0.06	0.62	0.60	0.64	0.60	0.08	0.64	0.65	0.05	0.62	0.60	0.64	0.59	0.08	0.64	0.64
	FCFP2	0.11	0.69	0.69	0.68	0.69	0.11	0.68	0.75	0.10	0.69	0.68	0.69	0.68	0.11	0.69	0.76
	FCFP4	0.13	0.71	0.72	0.69	0.72	0.12	0.69	0.78	0.12	0.70	0.72	0.68	0.72	0.12	0.68	0.77
	FCFP6	0.14	0.72	0.74	0.69	0.74	0.13	0.69	0.78	0.14	0.71	0.74	0.67	0.75	0.13	0.67	0.78
	KR	0.12	0.71	0.71	0.70	0.71	0.12	0.70	0.77	0.13	0.70	0.73	0.67	0.73	0.12	0.67	0.76
	LSTAR	0.11	0.69	0.70	0.68	0.70	0.11	0.68	0.76	0.13	0.70	0.74	0.66	0.74	0.12	0.66	0.76
	MACCS	0.13	0.71	0.72	0.70	0.72	0.12	0.70	0.78	0.13	0.71	0.72	0.70	0.72	0.12	0.70	0.77
	PUBCHEM	0.13	0.71	0.72	0.70	0.72	0.12	0.70	0.78	0.14	0.73	0.73	0.73	0.73	0.13	0.73	0.80
	RAD2D	0.13	0.71	0.73	0.68	0.73	0.12	0.68	0.77	0.14	0.72	0.74	0.70	0.74	0.13	0.70	0.79
	2PPHAR	0.02	0.52	0.76	0.24	0.81	NaN	0.24	0.59	0.02	0.53	0.76	0.25	0.82	NA	0.25	0.65
	3PPHAR	0.09	0.56	0.81	0.24	0.87	0.16	0.24	0.57	0.12	0.57	0.81	0.27	0.87	0.18	0.27	0.56
	AP2D	0.16	0.61	0.80	0.37	0.85	0.21	0.37	0.64	0.14	0.59	0.81	0.31	0.86	0.19	0.31	0.69
	ASP	0.16	0.61	0.80	0.38	0.84	0.21	0.38	0.67	0.12	0.56	0.82	0.23	0.89	0.27	0.23	0.67
	AT2D	0.17	0.60	0.85	0.28	0.91	0.24	0.28	0.67	0.05	0.52	0.83	0.15	0.90	0.14	0.15	0.63
	DFS	0.18	0.64	0.77	0.48	0.80	0.21	0.48	0.67	0.19	0.65	0.80	0.46	0.83	0.22	0.46	0.65
	ECFP0	0.07	0.56	0.74	0.33	0.78	0.14	0.33	0.58	0.15	0.62	0.74	0.48	0.77	0.18	0.48	0.65
	ECFP2	0.24	0.64	0.84	0.38	0.89	0.29	0.38	0.69	0.10	0.56	0.80	0.27	0.86	0.17	0.27	0.64
Multidrug Toxin Extrusion Transporter 1 Inhibition	ECFP4	0.17	0.60	0.82	0.33	0.88	0.23	0.33	0.67	0.05	0.52	0.88	0.08	0.96	NA	0.08	0.65
	ECFP6	0.18	0.61	0.82	0.35	0.87	0.22	0.35	0.69	0.03	0.52	0.76	0.23	0.81	0.11	0.23	0.66
	ESTATE	0.13	0.62	0.73	0.48	0.76	0.17	0.48	0.64	0.09	0.59	0.70	0.46	0.73	0.14	0.46	0.62
	FCFP0	0.09	0.60	0.68	0.49	0.70	0.15	0.49	0.65	0.06	0.56	0.69	0.40	0.72	0.13	0.40	0.58
	FCFP2	0.17	0.62	0.81	0.38	0.85	0.21	0.38	0.70	0.27	0.68	0.83	0.50	0.87	0.28	0.50	0.76
	FCFP4	0.12	0.57	0.83	0.25	0.89	0.20	0.25	0.65	0.14	0.57	0.85	0.23	0.92	0.31	0.23	0.68
	FCFP6	0.14	0.59	0.79	0.35	0.84	0.20	0.35	0.63	0.04	0.51	0.81	0.15	0.88	0.23	0.15	0.65
	KR	0.17	0.60	0.83	0.31	0.89	0.23	0.31	0.67	0.00	0.50	0.83	0.10	0.90	0.08	0.10	0.54
	LSTAR	0.14	0.59	0.82	0.30	0.88	0.21	0.30	0.67	0.09	0.56	0.84	0.21	0.91	NA	0.21	0.62
	MACCS	0.18	0.61	0.82	0.36	0.86	0.23	0.36	0.66	0.18	0.62	0.81	0.40	0.85	0.22	0.40	0.75
	PUBCHEM	0.20	0.62	0.82	0.38	0.87	0.24	0.38	0.68	0.11	0.57	0.80	0.29	0.85	0.17	0.29	0.64
	RAD2D	0.17	0.60	0.83	0.32	0.88	0.23	0.32	0.68	0.20	0.61	0.85	0.31	0.91	0.26	0.31	0.70
	2PPHAR	0.18	0.58	0.60	0.24	0.93	0.76	0.24	0.62	0.17	0.58	0.60	0.24	0.93	0.75	0.24	0.64
	3PPHAR	0.21	0.60	0.62	0.32	0.88	0.71	0.32	0.68	0.24	0.62	0.63	0.32	0.92	0.77	0.32	0.70
	AP2D	0.51	0.75	0.76	0.69	0.82	0.77	0.69	0.83	0.56	0.78	0.78	0.71	0.85	0.81	0.71	0.85
	ASP	0.60	0.80	0.80	0.85	0.75	0.75	0.85	0.87	0.60	0.80	0.80	0.81	0.79	0.78	0.81	0.88
	AT2D	0.59	0.80	0.79	0.85	0.74	0.75	0.85	0.87	0.57	0.79	0.79	0.82	0.76	0.75	0.82	0.86
	DFS	0.58	0.79	0.79	0.85	0.73	0.74	0.85	0.87	0.54	0.77	0.77	0.86	0.69	0.71	0.86	0.85
	ECFP0	0.53	0.76	0.77	0.71	0.81	0.77	0.71	0.84	0.51	0.75	0.76	0.70	0.81	0.77	0.70	0.85
	ECFP2	0.58	0.79	0.79	0.75	0.83	0.80	0.75	0.87	0.61	0.80	0.81	0.78	0.83	0.81	0.78	0.89
	ECFP4	0.58	0.79	0.79	0.77	0.81	0.78	0.77	0.87	0.57	0.78	0.78	0.76	0.80	0.78	0.76	0.87
P-glycoprotein Substrate	ECFP6	0.54	0.77	0.77	0.78	0.76	0.74	0.78	0.85	0.61	0.80	0.80	0.82	0.78	0.78	0.82	0.88
	ESTATE	0.51	0.75	0.75	0.71	0.80	0.76	0.71	0.82	0.54	0.77	0.77	0.73	0.80	0.77	0.73	0.85
	FCFP0	0.22	0.61	0.62	0.34	0.87	0.70	0.34	0.69	0.24	0.62	0.63	0.35	0.88	0.72	0.35	0.70

Continued on next page

Table S1 – *Continued from previous page*

Endpoint	FP	Calibration												Validation			
		κ	BACC	ACC	Se	Sp	AUC	κ	BACC	ACC	Se	Sp	AUC				
	FCFP2	0.59	0.79	0.80	0.75	0.84	0.80	0.75	0.88	0.57	0.78	0.78	0.72	0.84	0.80	0.72	0.86
	FCFP4	0.59	0.79	0.80	0.76	0.83	0.80	0.76	0.87	0.61	0.80	0.81	0.79	0.82	0.80	0.79	0.89
	FCFP6	0.59	0.79	0.79	0.82	0.77	0.76	0.82	0.86	0.62	0.81	0.81	0.85	0.77	0.77	0.85	0.88
	KR	0.57	0.78	0.79	0.72	0.85	0.81	0.72	0.86	0.59	0.79	0.80	0.73	0.85	0.82	0.73	0.89
	LSTAR	0.56	0.78	0.78	0.79	0.77	0.76	0.79	0.85	0.57	0.79	0.79	0.82	0.76	0.75	0.82	0.87
	MACCS	0.60	0.80	0.80	0.78	0.82	0.79	0.78	0.87	0.58	0.79	0.79	0.76	0.82	0.79	0.76	0.87
	PUBCHEM	0.57	0.78	0.79	0.74	0.82	0.79	0.74	0.86	0.58	0.79	0.79	0.77	0.82	0.79	0.77	0.88
	RAD2D	0.57	0.79	0.79	0.73	0.84	0.80	0.73	0.87	0.57	0.79	0.79	0.75	0.82	0.79	0.75	0.87
	2PPHAR	0.30	0.64	0.68	0.40	0.89	0.72	0.40	0.70	0.27	0.63	0.66	0.36	0.89	0.71	0.36	0.69
	3PPHAR	0.32	0.65	0.69	0.39	0.91	0.76	0.39	0.72	0.29	0.64	0.68	0.34	0.93	0.78	0.34	0.73
	AP2D	0.54	0.77	0.78	0.72	0.82	0.75	0.72	0.85	0.58	0.79	0.80	0.76	0.83	0.76	0.76	0.86
	ASP	0.64	0.82	0.82	0.83	0.82	0.77	0.83	0.90	0.65	0.83	0.83	0.85	0.81	0.77	0.85	0.91
	AT2D	0.64	0.83	0.82	0.85	0.81	0.77	0.85	0.90	0.68	0.84	0.84	0.86	0.83	0.79	0.86	0.91
	DFS	0.63	0.82	0.82	0.83	0.81	0.76	0.83	0.89	0.63	0.82	0.82	0.83	0.81	0.76	0.83	0.90
	ECFP0	0.51	0.76	0.76	0.76	0.76	0.70	0.76	0.83	0.53	0.77	0.77	0.74	0.80	0.73	0.74	0.84
	ECFP2	0.62	0.81	0.81	0.77	0.85	0.79	0.77	0.90	0.64	0.82	0.82	0.78	0.85	0.80	0.78	0.91
	ECFP4	0.61	0.80	0.81	0.77	0.84	0.78	0.77	0.89	0.62	0.81	0.82	0.75	0.86	0.80	0.75	0.90
P-glycoprotein Inhibitor	ECFP6	0.57	0.78	0.79	0.75	0.82	0.75	0.75	0.87	0.60	0.80	0.81	0.76	0.84	0.78	0.76	0.89
	ESTATE	0.44	0.72	0.73	0.66	0.78	0.69	0.66	0.80	0.44	0.72	0.73	0.61	0.82	0.72	0.61	0.81
	FCFP0	0.26	0.63	0.66	0.41	0.84	0.66	0.41	0.67	0.23	0.61	0.64	0.38	0.83	0.64	0.38	0.67
	FCFP2	0.59	0.80	0.80	0.77	0.82	0.76	0.77	0.88	0.59	0.80	0.80	0.77	0.82	0.76	0.77	0.89
	FCFP4	0.62	0.81	0.81	0.77	0.85	0.79	0.77	0.90	0.66	0.83	0.84	0.81	0.85	0.80	0.81	0.91
	FCFP6	0.58	0.79	0.79	0.76	0.82	0.76	0.76	0.88	0.61	0.80	0.81	0.75	0.85	0.79	0.75	0.89
	KR	0.62	0.81	0.81	0.77	0.85	0.79	0.77	0.89	0.65	0.83	0.83	0.81	0.84	0.79	0.81	0.90
	LSTAR	0.60	0.80	0.81	0.76	0.84	0.78	0.76	0.89	0.63	0.81	0.82	0.78	0.85	0.79	0.78	0.89
	MACCS	0.64	0.82	0.82	0.83	0.82	0.77	0.83	0.90	0.67	0.84	0.84	0.86	0.82	0.78	0.86	0.91
	PUBCHEM	0.67	0.84	0.84	0.83	0.85	0.80	0.83	0.91	0.69	0.85	0.85	0.83	0.86	0.82	0.83	0.92
	RAD2D	0.65	0.82	0.83	0.79	0.86	0.81	0.79	0.90	0.68	0.84	0.84	0.82	0.85	0.81	0.82	0.91
	2PPHAR	0.27	0.67	0.63	0.57	0.77	0.86	0.57	0.70	0.25	0.66	0.61	0.53	0.79	0.86	0.53	0.69
	3PPHAR	0.19	0.60	0.66	0.74	0.46	0.77	0.74	0.68	0.20	0.60	0.67	0.76	0.44	0.77	0.76	0.69
	AP2D	0.43	0.72	0.76	0.81	0.64	0.85	0.81	0.80	0.45	0.73	0.77	0.83	0.62	0.84	0.83	0.82
	ASP	0.52	0.76	0.80	0.86	0.65	0.86	0.86	0.84	0.54	0.77	0.81	0.87	0.66	0.86	0.87	0.85
	AT2D	0.47	0.73	0.78	0.85	0.62	0.85	0.85	0.81	0.53	0.76	0.81	0.88	0.64	0.86	0.88	0.84
	DFS	0.51	0.76	0.79	0.84	0.69	0.87	0.84	0.84	0.52	0.77	0.80	0.83	0.70	0.87	0.83	0.85
	ECFP0	0.44	0.73	0.76	0.82	0.63	0.85	0.82	0.80	0.49	0.75	0.79	0.83	0.67	0.86	0.83	0.84
	ECFP2	0.52	0.76	0.81	0.88	0.63	0.86	0.88	0.84	0.51	0.75	0.81	0.88	0.62	0.85	0.88	0.84
	ECFP4	0.50	0.74	0.80	0.88	0.61	0.85	0.88	0.84	0.58	0.79	0.83	0.89	0.69	0.88	0.89	0.87
Phospholipidosis	ECFP6	0.50	0.75	0.80	0.87	0.62	0.85	0.87	0.83	0.52	0.74	0.81	0.91	0.58	0.84	0.91	0.85
	ESTATE	0.43	0.72	0.76	0.81	0.64	0.85	0.81	0.80	0.47	0.74	0.77	0.82	0.66	0.86	0.82	0.81
	FCFP0	0.46	0.74	0.77	0.81	0.67	0.86	0.81	0.79	0.46	0.74	0.77	0.82	0.66	0.86	0.82	0.80
	FCFP2	0.56	0.78	0.81	0.86	0.71	0.88	0.86	0.86	0.54	0.77	0.81	0.87	0.68	0.87	0.87	0.88
	FCFP4	0.55	0.78	0.81	0.86	0.69	0.87	0.86	0.86	0.51	0.75	0.80	0.87	0.64	0.86	0.87	0.85
	FCFP6	0.56	0.78	0.82	0.87	0.69	0.87	0.87	0.86	0.55	0.77	0.82	0.89	0.65	0.86	0.89	0.86
	KR	0.52	0.76	0.80	0.87	0.64	0.86	0.87	0.84	0.52	0.75	0.81	0.90	0.60	0.84	0.90	0.85
	LSTAR	0.49	0.74	0.79	0.87	0.62	0.85	0.87	0.83	0.51	0.74	0.81	0.89	0.60	0.85	0.89	0.85
	MACCS	0.53	0.77	0.81	0.85	0.68	0.87	0.85	0.85	0.50	0.76	0.79	0.84	0.68	0.87	0.84	0.86
	PUBCHEM	0.52	0.75	0.80	0.87	0.64	0.86	0.87	0.84	0.50	0.74	0.80	0.89	0.60	0.84	0.89	0.84
	RAD2D	0.53	0.76	0.81	0.88	0.63	0.85	0.88	0.85	0.49	0.73	0.81	0.92	0.53	0.83	0.92	0.84
	2PPHAR	0.22	0.61	0.71	0.82	0.40	0.82	0.82	0.73	0.23	0.60	0.75	0.91	0.29	0.80	0.91	0.72
	3PPHAR	0.27	0.63	0.74	0.84	0.43	0.82	0.84	0.72	0.25	0.62	0.74	0.85	0.39	0.81	0.85	0.72
	AP2D	0.36	0.69	0.75	0.82	0.55	0.85	0.82	0.77	0.27	0.63	0.74	0.85	0.40	0.81	0.85	0.74
	ASP	0.44	0.73	0.78	0.83	0.63	0.87	0.83	0.80	0.48	0.73	0.82	0.91	0.54	0.86	0.91	0.81
	AT2D	0.45	0.74	0.78	0.82	0.66	0.88	0.82	0.80	0.44	0.74	0.77	0.81	0.66	0.88	0.81	0.80
	DFS	0.42	0.72	0.78	0.84	0.59	0.86	0.84	0.79	0.43	0.72	0.78	0.84	0.61	0.87	0.84	0.82
	ECFP0	0.34	0.69	0.73	0.78	0.60	0.86	0.78	0.76	0.31	0.67	0.72	0.76	0.59	0.85	0.76	0.76
	ECFP2	0.42	0.71	0.79	0.86	0.56	0.86	0.86	0.80	0.43	0.70	0.80	0.91	0.48	0.84	0.91	0.80
	ECFP4	0.44	0.72	0.80	0.88	0.55	0.86	0.88	0.79	0.44	0.71	0.80	0.90	0.52	0.85	0.90	0.80
OATP1B1 inhibition	ECFP6	0.46	0.72	0.80	0.89	0.55	0.86	0.89	0.80	0.50	0.73	0.83	0.93	0.53	0.86	0.93	0.82
	ESTATE	0.31	0.67	0.72	0.77	0.57	0.84	0.77	0.73	0.31	0.67	0.73	0.79	0.54	0.84	0.79	0.72
	FCFP0	0.13	0.58	0.63	0.67	0.48	0.80	0.67	0.63	0.13	0.57	0.65	0.73	0.40	0.79	0.73	0.66
	FCFP2	0.43	0.71	0.79	0.86	0.56	0.86	0.86	0.79	0.42	0.70	0.79	0.88	0.52	0.85	0.88	0.79
	FCFP4	0.43	0.71	0.79	0.88	0.54	0.85	0.88	0.79	0.46	0.71	0.82	0.93	0.48	0.84	0.93	0.81
	FCFP6	0.43	0.71	0.79	0.86	0.56	0.86	0.86	0.78	0.47	0.72	0.82	0.92	0.52	0.85	0.92	0.83
	KR	0.44	0.71	0.80	0.88	0.55	0.86	0.88	0.80	0.33	0.65	0.76	0.87	0.43	0.82	0.87	0.76
	LSTAR	0.43	0.71	0.79	0.87	0.55	0.86	0.87	0.79	0.49	0.73	0.82	0.91	0.55	0.86	0.91	0.80
	MACCS	0.46	0.74	0.79	0.84	0.64	0.88	0.84	0.80	0.39	0.71	0.76	0.82	0.60	0.86	0.82	0.77
	PUBCHEM	0.45	0.73	0.80	0.86	0.59	0.86	0.86	0.80	0.50	0.75	0.81	0.87	0.64	0.88	0.87	0.84
	RAD2D	0.44	0.71	0.81	0.90	0.51	0.85	0.90	0.81	0.45	0.70	0.82	0.93	0.47	0.84	0.93	0.83
OATP2B1 inhibition	2PPHAR	0.28	0.63	0.72	0.83	0.44	0.81	0.83	0.71	0.23	0.60	0.75	0.92	0.28	0.78	0.92	0.73

Continued on next page

Table S1 – *Continued from previous page*

Endpoint	FP	Calibration						Validation									
		κ	BACC	ACC	Se	Sp	AUC	κ	BACC	ACC	Se	Sp	AUC				
	3PPHAR	0.15	0.57	0.68	0.81	0.33	0.77	0.81	0.59	0.09	0.54	0.66	0.80	0.28	0.75	0.80	0.62
	AP2D	0.22	0.62	0.68	0.75	0.49	0.80	0.75	0.68	0.09	0.54	0.65	0.78	0.31	0.76	0.78	0.58
	ASP	0.31	0.66	0.72	0.79	0.53	0.82	0.79	0.71	0.20	0.60	0.67	0.76	0.44	0.79	0.76	0.68
	AT2D	0.32	0.67	0.72	0.79	0.54	0.83	0.79	0.69	0.20	0.59	0.71	0.85	0.33	0.78	0.85	0.62
	DFS	0.30	0.67	0.69	0.72	0.62	0.84	0.72	0.69	0.22	0.62	0.67	0.74	0.50	0.80	0.74	0.66
	ECFP0	0.28	0.65	0.71	0.77	0.53	0.82	0.77	0.70	0.23	0.61	0.71	0.83	0.39	0.79	0.83	0.63
	ECFP2	0.32	0.65	0.74	0.84	0.47	0.81	0.84	0.74	0.22	0.61	0.71	0.83	0.39	0.79	0.83	0.57
	ECFP4	0.32	0.65	0.75	0.86	0.45	0.81	0.86	0.70	0.48	0.72	0.81	0.93	0.50	0.84	0.93	0.76
	ECFP6	0.35	0.67	0.76	0.86	0.48	0.82	0.86	0.68	0.30	0.64	0.73	0.82	0.47	0.81	0.82	0.70
	ESTATE	0.25	0.64	0.68	0.73	0.55	0.82	0.73	0.68	0.32	0.68	0.72	0.77	0.58	0.84	0.77	0.74
	FCFP0	0.20	0.60	0.69	0.79	0.41	0.79	0.79	0.64	0.09	0.55	0.64	0.76	0.33	0.76	0.76	0.59
	FCFP2	0.27	0.63	0.73	0.84	0.43	0.80	0.84	0.70	0.21	0.59	0.71	0.85	0.33	0.78	0.85	0.70
	FCFP4	0.26	0.62	0.73	0.85	0.39	0.79	0.85	0.68	0.34	0.67	0.75	0.84	0.50	0.83	0.84	0.77
	FCFP6	0.32	0.66	0.74	0.83	0.48	0.81	0.83	0.73	0.10	0.55	0.69	0.85	0.25	0.76	0.85	0.64
	KR	0.29	0.64	0.75	0.88	0.39	0.80	0.88	0.71	0.24	0.61	0.73	0.86	0.36	0.79	0.86	0.65
	LSTAR	0.29	0.63	0.74	0.88	0.39	0.79	0.88	0.72	0.34	0.65	0.77	0.91	0.39	0.80	0.91	0.81
	MACCS	0.28	0.64	0.70	0.78	0.51	0.81	0.78	0.68	0.25	0.63	0.70	0.79	0.47	0.81	0.79	0.69
	PUBCHEM	0.29	0.64	0.72	0.81	0.47	0.81	0.81	0.70	0.27	0.63	0.72	0.82	0.44	0.80	0.82	0.66
	RAD2D	0.23	0.60	0.73	0.87	0.33	0.78	0.87	0.62	0.17	0.58	0.74	0.93	0.22	0.77	0.93	0.61
OATP1B3 inhibition	2PPHAR	0.23	0.71	0.62	0.58	0.84	0.95	0.58	0.74	0.22	0.69	0.63	0.60	0.79	0.94	0.60	0.75
	3PPHAR	0.30	0.70	0.75	0.78	0.62	0.92	0.78	0.78	0.25	0.65	0.76	0.81	0.50	0.90	0.81	0.71
	AP2D	0.29	0.68	0.77	0.81	0.55	0.90	0.81	0.75	0.32	0.69	0.79	0.84	0.54	0.91	0.84	0.80
	ASP	0.40	0.72	0.82	0.87	0.57	0.91	0.87	0.80	0.41	0.72	0.83	0.88	0.56	0.92	0.88	0.81
	AT2D	0.42	0.74	0.82	0.86	0.63	0.92	0.86	0.81	0.25	0.64	0.79	0.85	0.42	0.89	0.85	0.74
	DFS	0.38	0.70	0.83	0.88	0.51	0.91	0.88	0.79	0.32	0.68	0.80	0.85	0.51	0.90	0.85	0.79
	ECFP0	0.26	0.67	0.74	0.77	0.57	0.91	0.77	0.73	0.31	0.70	0.76	0.79	0.62	0.92	0.79	0.75
	ECFP2	0.40	0.70	0.83	0.89	0.52	0.91	0.89	0.80	0.33	0.67	0.82	0.89	0.44	0.90	0.89	0.82
	ECFP4	0.40	0.70	0.83	0.89	0.52	0.91	0.89	0.80	0.33	0.67	0.81	0.88	0.47	0.90	0.88	0.79
	ECFP6	0.36	0.70	0.81	0.86	0.53	0.91	0.86	0.79	0.42	0.69	0.86	0.93	0.46	0.90	0.93	0.86
	ESTATE	0.25	0.66	0.74	0.78	0.55	0.90	0.78	0.71	0.26	0.66	0.75	0.78	0.55	0.90	0.78	0.71
	FCFP0	0.10	0.57	0.67	0.72	0.42	0.87	0.72	0.63	0.07	0.53	0.74	0.83	0.24	0.85	0.83	0.63
	FCFP2	0.36	0.70	0.81	0.86	0.54	0.91	0.86	0.78	0.42	0.74	0.83	0.87	0.60	0.92	0.87	0.83
	FCFP4	0.40	0.70	0.84	0.90	0.51	0.91	0.90	0.80	0.37	0.69	0.83	0.89	0.48	0.90	0.89	0.80
	FCFP6	0.40	0.72	0.83	0.88	0.56	0.91	0.88	0.80	0.31	0.66	0.81	0.87	0.45	0.90	0.87	0.77
	KR	0.39	0.71	0.83	0.89	0.53	0.91	0.89	0.80	0.36	0.69	0.82	0.88	0.50	0.91	0.88	0.79
	LSTAR	0.36	0.69	0.82	0.87	0.51	0.91	0.87	0.78	0.37	0.68	0.84	0.91	0.45	0.90	0.91	0.81
	MACCS	0.40	0.72	0.82	0.86	0.59	0.92	0.86	0.81	0.40	0.74	0.82	0.86	0.62	0.92	0.86	0.81
	PUBCHEM	0.45	0.74	0.84	0.89	0.59	0.92	0.89	0.83	0.48	0.77	0.85	0.88	0.66	0.93	0.88	0.86
	RAD2D	0.42	0.71	0.84	0.91	0.51	0.91	0.91	0.80	0.39	0.68	0.85	0.92	0.44	0.90	0.92	0.80
Human Bile Salt Export Pump Inhibition	2PPHAR	0.38	0.70	0.70	0.69	0.70	0.61	0.69	0.76	0.41	0.71	0.71	0.68	0.74	0.64	0.68	0.78
	3PPHAR	0.41	0.70	0.72	0.62	0.78	0.67	0.62	0.80	0.46	0.73	0.74	0.65	0.81	0.70	0.65	0.82
	AP2D	0.65	0.82	0.83	0.78	0.86	0.79	0.78	0.89	0.65	0.83	0.83	0.82	0.83	0.77	0.82	0.91
	ASP	0.68	0.84	0.85	0.78	0.89	0.83	0.78	0.92	0.71	0.86	0.86	0.85	0.87	0.81	0.85	0.94
	AT2D	0.71	0.85	0.86	0.81	0.90	0.84	0.81	0.93	0.69	0.84	0.85	0.79	0.89	0.83	0.79	0.93
	DFS	0.68	0.84	0.85	0.79	0.88	0.82	0.79	0.92	0.74	0.86	0.88	0.80	0.93	0.88	0.80	0.94
	ECFP0	0.63	0.81	0.82	0.75	0.87	0.80	0.75	0.88	0.62	0.81	0.82	0.75	0.86	0.79	0.75	0.87
	ECFP2	0.69	0.84	0.85	0.77	0.91	0.85	0.77	0.92	0.77	0.88	0.89	0.82	0.93	0.90	0.82	0.95
	ECFP4	0.72	0.85	0.86	0.80	0.91	0.86	0.80	0.93	0.76	0.88	0.89	0.82	0.94	0.90	0.82	0.95
	ECFP6	0.70	0.85	0.85	0.80	0.89	0.83	0.80	0.93	0.72	0.85	0.87	0.79	0.91	0.86	0.79	0.95
	ESTATE	0.59	0.79	0.80	0.74	0.85	0.77	0.74	0.86	0.67	0.83	0.84	0.79	0.87	0.81	0.79	0.90
	FCFP0	0.26	0.62	0.66	0.44	0.80	0.60	0.44	0.69	0.22	0.60	0.65	0.38	0.82	0.61	0.38	0.67
	FCFP2	0.64	0.82	0.83	0.74	0.89	0.82	0.74	0.90	0.69	0.84	0.85	0.78	0.90	0.85	0.78	0.92
	FCFP4	0.68	0.84	0.85	0.76	0.91	0.85	0.76	0.92	0.72	0.85	0.87	0.76	0.94	0.90	0.76	0.93
	FCFP6	0.68	0.84	0.85	0.78	0.90	0.84	0.78	0.93	0.76	0.88	0.89	0.83	0.92	0.88	0.83	0.95
	KR	0.65	0.82	0.84	0.75	0.89	0.83	0.75	0.91	0.76	0.87	0.88	0.82	0.93	0.89	0.82	0.95
	LSTAR	0.68	0.84	0.85	0.80	0.88	0.81	0.80	0.92	0.72	0.86	0.87	0.84	0.88	0.83	0.84	0.94
	MACCS	0.69	0.84	0.85	0.78	0.90	0.84	0.78	0.91	0.73	0.86	0.87	0.82	0.91	0.86	0.82	0.94
	PUBCHEM	0.70	0.85	0.85	0.80	0.89	0.83	0.80	0.92	0.69	0.84	0.85	0.76	0.91	0.86	0.76	0.93
	RAD2D	0.66	0.82	0.84	0.73	0.92	0.86	0.73	0.92	0.69	0.84	0.86	0.75	0.93	0.87	0.75	0.93
Organic Cation Transporter 2 Inhibition	2PPHAR	0.20	0.63	0.63	0.62	0.63	0.38	0.62	0.67	0.27	0.68	0.63	0.78	0.58	0.40	0.78	0.68
	3PPHAR	0.17	0.59	0.66	0.43	0.75	0.39	0.43	0.66	0.13	0.56	0.68	0.33	0.80	0.37	0.33	0.66
	AP2D	0.37	0.69	0.74	0.59	0.80	0.53	0.59	0.76	0.39	0.71	0.75	0.61	0.80	0.53	0.61	0.78
	ASP	0.37	0.69	0.74	0.59	0.80	0.52	0.59	0.76	0.36	0.67	0.76	0.48	0.86	0.56	0.48	0.79
	AT2D	0.41	0.71	0.76	0.60	0.82	0.56	0.60	0.78	0.44	0.72	0.78	0.60	0.85	0.58	0.60	0.81
	DFS	0.35	0.69	0.73	0.59	0.79	0.50	0.59	0.74	0.38	0.69	0.76	0.56	0.83	0.55	0.56	0.76
	ECFP0	0.37	0.70	0.73	0.64	0.76	0.50	0.64	0.77	0.40	0.71	0.75	0.65	0.78	0.52	0.65	0.80
	ECFP2	0.42	0.71	0.77	0.58	0.84	0.58	0.58	0.78	0.47	0.73	0.79	0.60	0.86	0.61	0.60	0.81
	ECFP4	0.40	0.70	0.76	0.55	0.84	0.57	0.55	0.77	0.36	0.66	0.77	0.44	0.88	0.58	0.44	0.77
	ECFP6	0.40	0.70	0.77	0.56	0.84	0.57	0.56	0.78	0.33	0.66	0.75	0.47	0.85	0.53	0.47	0.75

Continued on next page

Table S1 – *Continued from previous page*

**Endpoint**	**FP**	**Calibration**											**Validation**				
		κ	BACC	ACC	Se	Sp	AUC	κ	BACC	ACC	Se	Sp	AUC				
	ESTATE	0.38	0.71	0.74	0.63	0.78	0.51	0.63	0.77	0.41	0.73	0.74	0.69	0.76	0.51	0.69	0.81
	FCFP0	0.33	0.67	0.72	0.56	0.78	0.49	0.56	0.70	0.36	0.69	0.74	0.58	0.80	0.52	0.58	0.73
	FCFP2	0.44	0.72	0.77	0.60	0.84	0.58	0.60	0.80	0.37	0.68	0.76	0.53	0.84	0.55	0.53	0.79
	FCFP4	0.43	0.71	0.78	0.56	0.86	0.60	0.56	0.79	0.52	0.75	0.82	0.60	0.89	0.69	0.60	0.82
	FCFP6	0.42	0.71	0.77	0.56	0.85	0.59	0.56	0.78	0.42	0.69	0.79	0.49	0.90	0.64	0.49	0.80
	KR	0.44	0.72	0.78	0.58	0.86	0.61	0.58	0.81	0.53	0.76	0.82	0.63	0.89	0.67	0.63	0.84
	LSTAR	0.33	0.67	0.72	0.57	0.77	0.49	0.57	0.74	0.32	0.66	0.74	0.50	0.82	0.53	0.50	0.75
	MACCS	0.47	0.75	0.78	0.66	0.83	0.59	0.66	0.82	0.39	0.70	0.76	0.56	0.83	0.55	0.56	0.77
	PUBCHEM	0.46	0.73	0.79	0.61	0.85	0.62	0.61	0.81	0.44	0.73	0.77	0.63	0.83	0.57	0.63	0.79
	RAD2D	0.45	0.72	0.78	0.59	0.85	0.60	0.59	0.80	0.39	0.69	0.77	0.53	0.86	0.57	0.53	0.80
Skin Sensitization (DPRA)	2PPHAR	0.12	0.56	0.61	0.32	0.79	0.48	0.32	0.51	-0.07	0.47	0.53	0.24	0.69	0.29	0.24	0.47
	3PPHAR	0.13	0.56	0.63	0.26	0.86	0.54	0.26	0.58	0.08	0.54	0.61	0.29	0.79	0.43	0.29	0.57
	AP2D	0.11	0.55	0.58	0.44	0.67	0.44	0.44	0.54	0.13	0.56	0.61	0.40	0.72	0.48	0.40	0.61
	ASP	0.23	0.61	0.65	0.46	0.77	0.56	0.46	0.62	0.28	0.62	0.70	0.33	0.92	0.74	0.33	0.65
	AT2D	0.24	0.62	0.64	0.52	0.72	0.54	0.52	0.62	0.28	0.65	0.63	0.71	0.58	0.51	0.71	0.68
	DFS	0.17	0.58	0.62	0.43	0.73	0.51	0.43	0.61	0.25	0.62	0.68	0.40	0.83	0.59	0.40	0.69
	ECFP0	0.23	0.62	0.63	0.59	0.65	0.51	0.59	0.62	0.37	0.70	0.68	0.76	0.64	0.55	0.76	0.73
	ECFP2	0.27	0.64	0.65	0.56	0.71	0.55	0.56	0.65	0.31	0.66	0.68	0.55	0.76	0.56	0.55	0.66
	ECFP4	0.26	0.62	0.66	0.48	0.77	0.56	0.48	0.67	0.16	0.58	0.61	0.45	0.71	0.47	0.45	0.61
	ECFP6	0.24	0.62	0.65	0.49	0.75	0.54	0.49	0.64	0.23	0.61	0.66	0.43	0.79	0.55	0.43	0.69
	ESTATE	0.25	0.63	0.63	0.60	0.65	0.52	0.60	0.61	0.03	0.52	0.54	0.45	0.58	0.38	0.45	0.54
	FCFP0	0.22	0.61	0.66	0.38	0.83	0.59	0.38	0.67	0.04	0.52	0.60	0.21	0.82	0.42	0.21	0.61
	FCFP2	0.27	0.64	0.66	0.54	0.74	0.56	0.54	0.65	0.20	0.60	0.63	0.48	0.72	0.50	0.48	0.67
	FCFP4	0.37	0.68	0.71	0.57	0.79	0.64	0.57	0.72	0.35	0.68	0.69	0.62	0.74	0.57	0.62	0.72
	FCFP6	0.32	0.66	0.69	0.56	0.76	0.59	0.56	0.73	0.37	0.68	0.71	0.57	0.79	0.62	0.57	0.70
	KR	0.30	0.65	0.68	0.51	0.79	0.61	0.51	0.66	0.04	0.52	0.55	0.40	0.64	0.40	0.40	0.55
	LSTAR	0.25	0.62	0.68	0.36	0.87	0.65	0.36	0.62	0.16	0.58	0.66	0.26	0.89	0.52	0.26	0.60
	MACCS	0.24	0.62	0.64	0.56	0.69	0.53	0.56	0.62	0.20	0.60	0.63	0.50	0.71	0.49	0.50	0.65
	PUBCHEM	0.21	0.60	0.62	0.55	0.66	0.51	0.55	0.62	0.15	0.58	0.59	0.55	0.61	0.45	0.55	0.61
	RAD2D	0.15	0.57	0.62	0.38	0.76	0.52	0.38	0.59	0.22	0.60	0.66	0.40	0.81	0.56	0.40	0.61
Skin Sensitization (LLNA)	2PPHAR	0.08	0.54	0.55	0.76	0.32	0.55	0.76	0.58	0.11	0.56	0.56	0.79	0.33	0.56	0.79	0.58
	3PPHAR	0.07	0.54	0.55	0.88	0.20	0.54	0.88	0.60	0.05	0.52	0.54	0.90	0.15	0.53	0.90	0.60
	AP2D	0.20	0.60	0.60	0.68	0.52	0.60	0.68	0.64	0.23	0.61	0.61	0.68	0.55	0.62	0.68	0.67
	ASP	0.32	0.66	0.66	0.80	0.52	0.64	0.80	0.73	0.29	0.64	0.65	0.78	0.51	0.63	0.78	0.72
	AT2D	0.28	0.64	0.64	0.58	0.70	0.68	0.58	0.69	0.26	0.63	0.63	0.53	0.73	0.68	0.53	0.66
	DFS	0.29	0.65	0.65	0.71	0.58	0.65	0.71	0.72	0.29	0.64	0.65	0.73	0.55	0.64	0.73	0.73
	ECFP0	0.30	0.65	0.65	0.68	0.63	0.66	0.68	0.70	0.34	0.67	0.67	0.74	0.60	0.66	0.74	0.74
	ECFP2	0.35	0.67	0.68	0.74	0.61	0.67	0.74	0.74	0.40	0.70	0.70	0.76	0.64	0.69	0.76	0.76
	ECFP4	0.27	0.63	0.64	0.74	0.53	0.63	0.74	0.71	0.32	0.66	0.66	0.80	0.52	0.64	0.80	0.73
	ECFP6	0.28	0.64	0.64	0.76	0.52	0.63	0.76	0.71	0.32	0.66	0.66	0.75	0.57	0.65	0.75	0.70
	ESTATE	0.32	0.66	0.66	0.68	0.64	0.68	0.68	0.72	0.32	0.66	0.66	0.70	0.62	0.66	0.70	0.72
	FCFP0	0.13	0.56	0.57	0.83	0.30	0.56	0.83	0.63	0.15	0.57	0.59	0.95	0.20	0.56	0.95	0.67
	FCFP2	0.37	0.69	0.69	0.72	0.65	0.69	0.72	0.74	0.29	0.64	0.65	0.71	0.58	0.65	0.71	0.70
	FCFP4	0.32	0.66	0.66	0.76	0.56	0.65	0.76	0.73	0.33	0.66	0.67	0.78	0.55	0.65	0.78	0.73
	FCFP6	0.36	0.68	0.68	0.75	0.61	0.68	0.75	0.74	0.34	0.67	0.67	0.73	0.61	0.67	0.73	0.72
	KR	0.32	0.66	0.67	0.76	0.56	0.65	0.76	0.74	0.29	0.64	0.64	0.73	0.56	0.64	0.73	0.73
	LSTAR	0.21	0.60	0.61	0.71	0.49	0.60	0.71	0.66	0.25	0.63	0.63	0.71	0.54	0.62	0.71	0.70
	MACCS	0.35	0.67	0.67	0.69	0.65	0.69	0.69	0.73	0.37	0.69	0.69	0.69	0.68	0.70	0.69	0.74
	PUBCHEM	0.39	0.69	0.70	0.74	0.65	0.69	0.74	0.76	0.33	0.67	0.67	0.71	0.62	0.67	0.71	0.74
	RAD2D	0.23	0.61	0.62	0.78	0.45	0.60	0.78	0.68	0.32	0.66	0.66	0.79	0.53	0.64	0.79	0.70
Skin Sensitization (KeratinSens)	2PPHAR	0.17	0.57	0.68	0.23	0.92	0.67	0.23	0.61	0.14	0.56	0.69	0.17	0.95	0.78	0.17	0.52
	3PPHAR	0.17	0.57	0.69	0.20	0.94	0.67	0.20	0.59	0.16	0.56	0.69	0.19	0.93	0.67	0.19	0.61
	AP2D	0.25	0.62	0.67	0.49	0.75	0.51	0.49	0.64	0.05	0.53	0.59	0.33	0.72	0.36	0.33	0.57
	ASP	0.19	0.58	0.69	0.26	0.91	0.64	0.26	0.64	0.23	0.60	0.68	0.36	0.84	0.66	0.36	0.64
	AT2D	0.18	0.58	0.64	0.42	0.75	0.48	0.42	0.59	0.14	0.57	0.66	0.31	0.83	0.63	0.31	0.60
	DFS	0.22	0.60	0.67	0.40	0.81	0.51	0.40	0.61	0.10	0.55	0.63	0.31	0.79	0.41	0.31	0.67
	ECFP0	0.17	0.59	0.60	0.53	0.64	0.44	0.53	0.61	-0.06	0.47	0.54	0.28	0.67	0.27	0.28	0.54
	ECFP2	0.20	0.60	0.64	0.46	0.74	0.48	0.46	0.62	0.15	0.56	0.68	0.25	0.88	0.54	0.25	0.59
	ECFP4	0.22	0.61	0.67	0.42	0.79	0.53	0.42	0.64	0.28	0.63	0.71	0.39	0.87	0.58	0.39	0.68
	ECFP6	0.23	0.61	0.67	0.41	0.80	0.56	0.41	0.65	-0.02	0.49	0.57	0.25	0.72	0.36	0.25	0.57
	ESTATE	0.22	0.62	0.62	0.62	0.62	0.46	0.62	0.66	-0.05	0.47	0.50	0.39	0.56	0.29	0.39	0.54
	FCFP0	0.19	0.59	0.65	0.43	0.76	0.50	0.43	0.59	0.18	0.58	0.70	0.22	0.93	0.72	0.22	0.57
	FCFP2	0.25	0.62	0.68	0.44	0.80	0.55	0.44	0.64	0.36	0.66	0.75	0.42	0.91	0.70	0.42	0.72
	FCFP4	0.24	0.62	0.67	0.46	0.78	0.53	0.46	0.63	0.35	0.68	0.71	0.58	0.77	0.55	0.58	0.75
	FCFP6	0.28	0.63	0.71	0.38	0.88	0.63	0.38	0.66	0.12	0.55	0.66	0.25	0.85	0.45	0.25	0.66
	KR	0.25	0.63	0.67	0.49	0.77	0.52	0.49	0.68	0.24	0.62	0.67	0.50	0.75	0.49	0.50	0.64
	LSTAR	0.30	0.64	0.72	0.39	0.89	0.68	0.39	0.65	0.15	0.57	0.67	0.28	0.85	0.49	0.28	0.60
	MACCS	0.18	0.59	0.62	0.51	0.67	0.46	0.51	0.63	0.09	0.55	0.60	0.39	0.71	0.39	0.39	0.61
	PUBCHEM	0.27	0.63	0.67	0.51	0.75	0.53	0.51	0.68	0.30	0.65	0.69	0.53	0.77	0.53	0.53	0.72

Continued on next page

Table S1 – *Continued from previous page*

**Endpoint**	**FP**	**Calibration**										**Validation**					
		κ	BACC	ACC	Se	Sp	AUC	κ	BACC	ACC	Se	Sp	AUC				
	RAD2D	0.27	0.62	0.70	0.40	0.85	0.61	0.40	0.65	0.11	0.55	0.62	0.33	0.76	0.44	0.33	0.61
Skin Sensitization (HRIPT)	2PPHAR	0.12	0.56	0.62	0.32	0.79	0.50	0.32	0.56	0.12	0.56	0.59	0.43	0.69	0.45	0.43	0.51
	3PPHAR	0.18	0.58	0.66	0.31	0.86	0.58	0.31	0.62	0.23	0.60	0.67	0.37	0.84	0.60	0.37	0.53
	AP2D	0.06	0.53	0.57	0.40	0.66	0.41	0.40	0.51	0.13	0.57	0.62	0.37	0.76	0.44	0.37	0.61
	ASP	0.23	0.61	0.67	0.39	0.83	0.58	0.39	0.61	0.16	0.58	0.62	0.43	0.73	0.49	0.43	0.68
	AT2D	0.29	0.64	0.67	0.56	0.73	0.55	0.56	0.67	0.13	0.57	0.59	0.47	0.67	0.47	0.47	0.59
	DFS	0.20	0.59	0.65	0.39	0.80	0.53	0.39	0.62	0.07	0.53	0.59	0.30	0.76	0.46	0.30	0.52
	ECFP0	0.38	0.70	0.70	0.67	0.72	0.60	0.67	0.74	0.33	0.67	0.67	0.70	0.65	0.55	0.70	0.72
	ECFP2	0.38	0.70	0.70	0.68	0.72	0.58	0.68	0.73	0.18	0.59	0.59	0.60	0.59	0.46	0.60	0.65
	ECFP4	0.26	0.63	0.67	0.49	0.76	0.56	0.49	0.67	0.03	0.51	0.56	0.33	0.69	0.40	0.33	0.66
	ECFP6	0.19	0.59	0.65	0.40	0.78	0.52	0.40	0.60	0.28	0.64	0.68	0.47	0.80	0.60	0.47	0.63
	ESTATE	0.33	0.67	0.67	0.66	0.68	0.56	0.66	0.72	0.26	0.64	0.62	0.73	0.55	0.49	0.73	0.60
	FCFP0	0.13	0.56	0.65	0.26	0.86	0.57	0.26	0.54	0.12	0.55	0.64	0.20	0.90	0.56	0.20	0.59
	FCFP2	0.30	0.65	0.68	0.53	0.76	0.56	0.53	0.71	0.22	0.62	0.62	0.63	0.61	0.48	0.63	0.63
	FCFP4	0.26	0.63	0.66	0.52	0.73	0.56	0.52	0.64	0.34	0.66	0.70	0.50	0.82	0.65	0.50	0.71
	FCFP6	0.17	0.58	0.64	0.38	0.78	0.51	0.38	0.59	0.43	0.72	0.73	0.70	0.75	0.62	0.70	0.75
	KR	0.23	0.61	0.66	0.46	0.77	0.52	0.46	0.63	0.31	0.65	0.69	0.50	0.80	0.60	0.50	0.74
	LSTAR	0.16	0.57	0.66	0.27	0.88	0.55	0.27	0.65	0.25	0.61	0.68	0.37	0.86	0.61	0.37	0.67
	MACCS	0.27	0.64	0.65	0.59	0.69	0.52	0.59	0.66	0.33	0.66	0.69	0.53	0.78	0.69	0.53	0.75
	PUBCHEM	0.27	0.63	0.65	0.56	0.71	0.54	0.56	0.64	0.13	0.56	0.61	0.40	0.73	0.47	0.40	0.59
	RAD2D	0.26	0.62	0.68	0.43	0.82	0.58	0.43	0.68	0.14	0.56	0.63	0.30	0.82	0.59	0.30	0.61
Skin Sensitization (h-CLAT)	2PPHAR	0.05	0.53	0.63	0.22	0.83	NaN	0.22	0.59	-0.05	0.48	0.61	0.10	0.86	NA	0.10	0.47
	3PPHAR	0.03	0.51	0.66	0.07	0.96	NaN	0.07	0.51	0.00	0.50	0.68	0.00	1.00	NA	0.00	0.38
	AP2D	0.14	0.57	0.61	0.46	0.68	0.41	0.46	0.57	0.15	0.58	0.60	0.50	0.65	0.43	0.50	0.57
	ASP	0.19	0.59	0.68	0.31	0.86	0.57	0.31	0.63	0.17	0.58	0.68	0.30	0.86	0.52	0.30	0.68
	AT2D	0.26	0.62	0.68	0.46	0.79	0.55	0.46	0.63	-0.02	0.49	0.57	0.27	0.71	0.31	0.27	0.49
	DFS	0.20	0.60	0.67	0.38	0.81	0.51	0.38	0.63	0.19	0.60	0.66	0.43	0.76	0.45	0.43	0.67
	ECFP0	0.21	0.62	0.62	0.60	0.63	0.45	0.60	0.63	0.08	0.54	0.66	0.20	0.87	NA	0.20	0.57
	ECFP2	0.15	0.57	0.63	0.39	0.76	0.47	0.39	0.60	0.24	0.61	0.68	0.43	0.79	0.53	0.43	0.59
	ECFP4	0.20	0.60	0.66	0.40	0.80	0.53	0.40	0.60	0.21	0.60	0.68	0.37	0.83	0.51	0.37	0.62
	ECFP6	0.25	0.62	0.69	0.39	0.85	0.58	0.39	0.67	0.03	0.51	0.61	0.23	0.79	0.33	0.23	0.51
	ESTATE	0.22	0.62	0.62	0.61	0.63	0.46	0.61	0.59	0.09	0.55	0.57	0.50	0.60	0.37	0.50	0.51
	FCFP0	0.03	0.51	0.61	0.23	0.79	0.41	0.23	0.54	-0.23	0.39	0.51	0.07	0.71	0.06	0.07	0.42
	FCFP2	0.26	0.63	0.68	0.47	0.78	0.52	0.47	0.69	0.08	0.54	0.62	0.30	0.78	0.38	0.30	0.62
	FCFP4	0.27	0.63	0.70	0.41	0.85	0.59	0.41	0.67	0.22	0.61	0.67	0.43	0.78	0.50	0.43	0.64
	FCFP6	0.25	0.62	0.69	0.41	0.82	0.54	0.41	0.66	0.40	0.69	0.75	0.50	0.87	0.68	0.50	0.68
	KR	0.27	0.63	0.69	0.44	0.82	0.57	0.44	0.68	0.35	0.66	0.74	0.43	0.89	0.67	0.43	0.70
	LSTAR	0.17	0.58	0.68	0.26	0.89	0.50	0.26	0.59	0.19	0.59	0.68	0.33	0.84	0.50	0.33	0.60
	MACCS	0.28	0.65	0.66	0.61	0.69	0.50	0.61	0.70	0.21	0.61	0.64	0.50	0.71	0.45	0.50	0.68
	PUBCHEM	0.30	0.65	0.68	0.57	0.74	0.54	0.57	0.71	0.14	0.58	0.60	0.50	0.65	0.41	0.50	0.65
	RAD2D	0.14	0.56	0.64	0.32	0.81	0.50	0.32	0.60	0.47	0.72	0.78	0.53	0.90	0.76	0.53	0.75
AMES Mutagenecity	2PPHAR	0.11	0.56	0.54	0.75	0.37	0.51	0.75	0.59	0.09	0.55	0.52	0.92	0.17	0.49	0.92	0.60
	3PPHAR	0.19	0.60	0.59	0.63	0.56	0.56	0.63	0.64	0.19	0.59	0.59	0.64	0.54	0.55	0.64	0.65
	AP2D	0.46	0.73	0.73	0.71	0.75	0.71	0.71	0.80	0.48	0.74	0.74	0.71	0.76	0.72	0.71	0.80
	ASP	0.57	0.78	0.79	0.75	0.82	0.78	0.75	0.85	0.58	0.79	0.79	0.75	0.82	0.79	0.75	0.86
	AT2D	0.56	0.78	0.78	0.74	0.81	0.78	0.74	0.84	0.55	0.77	0.78	0.75	0.80	0.76	0.75	0.84
	DFS	0.56	0.78	0.78	0.75	0.81	0.78	0.75	0.85	0.59	0.79	0.80	0.76	0.82	0.79	0.76	0.86
	ECFP0	0.47	0.74	0.74	0.72	0.75	0.72	0.72	0.81	0.48	0.74	0.74	0.72	0.76	0.72	0.72	0.81
	ECFP2	0.56	0.78	0.78	0.76	0.80	0.77	0.76	0.85	0.59	0.80	0.80	0.77	0.82	0.79	0.77	0.87
	ECFP4	0.57	0.79	0.79	0.75	0.82	0.79	0.75	0.86	0.58	0.79	0.79	0.76	0.82	0.78	0.76	0.86
	ECFP6	0.55	0.78	0.78	0.74	0.81	0.77	0.74	0.84	0.58	0.79	0.79	0.76	0.82	0.79	0.76	0.86
	ESTATE	0.47	0.73	0.73	0.72	0.75	0.71	0.72	0.81	0.47	0.73	0.74	0.71	0.76	0.72	0.71	0.81
	FCFP0	0.24	0.62	0.62	0.68	0.56	0.57	0.68	0.66	0.24	0.62	0.62	0.66	0.58	0.58	0.66	0.66
	FCFP2	0.52	0.76	0.76	0.74	0.79	0.75	0.74	0.83	0.52	0.76	0.76	0.73	0.79	0.75	0.73	0.83
	FCFP4	0.55	0.77	0.77	0.74	0.80	0.77	0.74	0.85	0.54	0.77	0.77	0.74	0.80	0.76	0.74	0.85
	FCFP6	0.55	0.77	0.78	0.74	0.81	0.77	0.74	0.84	0.55	0.78	0.78	0.75	0.80	0.76	0.75	0.85
	KR	0.56	0.78	0.78	0.77	0.79	0.76	0.77	0.85	0.60	0.80	0.80	0.79	0.82	0.79	0.79	0.87
	LSTAR	0.54	0.77	0.77	0.76	0.77	0.75	0.76	0.84	0.56	0.78	0.78	0.77	0.80	0.77	0.77	0.85
	MACCS	0.58	0.79	0.79	0.76	0.82	0.78	0.76	0.85	0.58	0.79	0.79	0.75	0.83	0.79	0.75	0.86
	PUBCHEM	0.59	0.79	0.79	0.77	0.82	0.79	0.77	0.86	0.58	0.79	0.79	0.76	0.83	0.79	0.76	0.87
	RAD2D	0.57	0.78	0.79	0.76	0.81	0.78	0.76	0.86	0.58	0.79	0.79	0.76	0.82	0.79	0.76	0.86
Hemolytic Toxicity (saponins)	2PPHAR	0.10	0.54	0.70	0.19	0.89	0.47	0.19	0.59	0.00	0.50	0.72	0.03	0.97	0.17	0.03	0.56
	3PPHAR	0.21	0.59	0.75	0.24	0.93	0.56	0.24	0.68	0.29	0.62	0.77	0.29	0.94	0.67	0.29	0.71
	AP2D	0.29	0.62	0.77	0.31	0.93	0.64	0.31	0.67	0.15	0.56	0.73	0.19	0.92	0.52	0.19	0.62
	ASP	0.62	0.81	0.85	0.71	0.90	0.74	0.71	0.88	0.69	0.84	0.88	0.75	0.93	0.80	0.75	0.90
	AT2D	0.65	0.82	0.86	0.74	0.91	0.76	0.74	0.89	0.60	0.80	0.84	0.71	0.89	0.71	0.71	0.85
	DFS	0.66	0.83	0.87	0.74	0.92	0.77	0.74	0.88	0.64	0.81	0.87	0.68	0.93	0.80	0.68	0.88
	ECFP0	0.26	0.62	0.73	0.40	0.85	0.50	0.40	0.71	0.34	0.66	0.75	0.47	0.85	0.54	0.47	0.75
	ECFP2	0.65	0.82	0.86	0.74	0.91	0.76	0.74	0.87	0.73	0.86	0.90	0.79	0.93	0.82	0.79	0.92

Continued on next page

Table S1 – *Continued from previous page*

Endpoint	FP				Calibration								Validation			
		κ	BACC	ACC	Se	Sp	AUC	κ	BACC	ACC	Se	Sp	AUC			
	ECFP4	0.63	0.81	0.86	0.71	0.92	0.77	0.71	0.86	0.62	0.80	0.86	0.68	0.92	0.76	0.68	0.91
	ECFP6	0.66	0.83	0.87	0.75	0.91	0.76	0.75	0.88	0.60	0.79	0.85	0.68	0.91	0.73	0.68	0.87
	ESTATE	0.32	0.65	0.75	0.46	0.85	0.53	0.46	0.69	0.19	0.59	0.71	0.35	0.84	0.43	0.35	0.65
	FCFP0	0.15	0.58	0.64	0.47	0.70	0.36	0.47	0.64	0.10	0.56	0.63	0.39	0.72	0.39	0.39	0.62
	FCFP2	0.36	0.67	0.75	0.50	0.85	0.55	0.50	0.76	0.42	0.71	0.78	0.54	0.87	0.60	0.54	0.81
	FCFP4	0.62	0.81	0.85	0.71	0.91	0.75	0.71	0.87	0.68	0.85	0.87	0.79	0.90	0.75	0.79	0.90
	FCFP6	0.68	0.84	0.88	0.75	0.92	0.79	0.75	0.88	0.71	0.85	0.89	0.76	0.93	0.82	0.76	0.90
	KR	0.54	0.77	0.82	0.67	0.88	0.68	0.67	0.85	0.54	0.76	0.82	0.64	0.89	0.68	0.64	0.86
	LSTAR	0.64	0.82	0.86	0.72	0.91	0.76	0.72	0.87	0.59	0.79	0.84	0.68	0.90	0.72	0.68	0.88
	MACCS	0.41	0.70	0.78	0.55	0.86	0.58	0.55	0.77	0.36	0.67	0.77	0.46	0.88	0.58	0.46	0.77
	PUBCHEM	0.45	0.72	0.79	0.56	0.87	0.63	0.56	0.80	0.34	0.66	0.76	0.43	0.88	0.57	0.43	0.75
	RAD2D	0.55	0.77	0.83	0.64	0.90	0.72	0.64	0.86	0.78	0.88	0.91	0.81	0.95	0.87	0.81	0.95
	2PPHAR	0.17	0.58	0.61	0.30	0.86	0.64	0.30	0.59	0.19	0.59	0.62	0.32	0.87	0.66	0.32	0.59
	3PPHAR	0.23	0.61	0.62	0.53	0.70	0.59	0.53	0.67	0.23	0.62	0.62	0.53	0.70	0.59	0.53	0.68
	AP2D	0.50	0.75	0.75	0.74	0.76	0.71	0.74	0.82	0.52	0.76	0.76	0.76	0.76	0.72	0.76	0.83
	ASP	0.55	0.78	0.78	0.76	0.79	0.75	0.76	0.84	0.55	0.77	0.77	0.77	0.78	0.74	0.77	0.85
	AT2D	0.56	0.78	0.78	0.76	0.79	0.75	0.76	0.85	0.58	0.79	0.79	0.78	0.80	0.76	0.78	0.87
	DFS	0.54	0.77	0.77	0.76	0.78	0.74	0.76	0.84	0.57	0.78	0.78	0.78	0.79	0.75	0.78	0.85
	ECFP0	0.47	0.74	0.74	0.74	0.73	0.69	0.74	0.81	0.50	0.75	0.75	0.76	0.74	0.71	0.76	0.83
	ECFP2	0.56	0.78	0.78	0.78	0.78	0.75	0.78	0.86	0.58	0.79	0.79	0.79	0.79	0.76	0.79	0.87
	ECFP4	0.56	0.78	0.78	0.78	0.78	0.75	0.78	0.86	0.59	0.80	0.80	0.79	0.81	0.77	0.79	0.88
hERG cardiotoxicity	ECFP6	0.56	0.78	0.78	0.77	0.79	0.75	0.77	0.85	0.56	0.78	0.78	0.77	0.79	0.75	0.77	0.86
	ESTATE	0.44	0.72	0.72	0.73	0.72	0.68	0.73	0.80	0.50	0.75	0.75	0.75	0.75	0.71	0.75	0.82
	FCFP0	0.27	0.63	0.64	0.57	0.70	0.61	0.57	0.69	0.26	0.63	0.63	0.63	0.63	0.58	0.63	0.69
	FCFP2	0.55	0.78	0.78	0.78	0.78	0.74	0.78	0.85	0.56	0.78	0.78	0.79	0.78	0.74	0.79	0.86
	FCFP4	0.58	0.79	0.79	0.79	0.80	0.76	0.79	0.87	0.60	0.80	0.80	0.80	0.80	0.77	0.80	0.88
	FCFP6	0.58	0.79	0.79	0.78	0.80	0.76	0.78	0.86	0.60	0.80	0.80	0.79	0.81	0.77	0.79	0.88
	KR	0.57	0.79	0.79	0.79	0.78	0.74	0.79	0.86	0.57	0.79	0.79	0.79	0.78	0.74	0.79	0.87
	LSTAR	0.56	0.78	0.78	0.76	0.80	0.75	0.76	0.85	0.56	0.78	0.78	0.75	0.82	0.77	0.75	0.86
	MACCS	0.56	0.78	0.78	0.78	0.78	0.75	0.78	0.85	0.58	0.79	0.79	0.78	0.80	0.76	0.78	0.86
	PUBCHEM	0.57	0.78	0.78	0.78	0.79	0.75	0.78	0.86	0.58	0.79	0.79	0.78	0.80	0.76	0.78	0.87
	RAD2D	0.57	0.79	0.79	0.78	0.80	0.76	0.78	0.86	0.59	0.79	0.80	0.78	0.81	0.77	0.78	0.87
	2PPHAR	0.16	0.58	0.77	0.88	0.28	0.85	0.88	0.61	0.15	0.57	0.79	0.91	0.22	0.85	0.91	0.61
	3PPHAR	0.26	0.61	0.83	0.94	0.28	0.86	0.94	0.72	0.23	0.60	0.82	0.94	0.25	0.86	0.94	0.71
	AP2D	0.48	0.74	0.85	0.91	0.57	0.91	0.91	0.83	0.47	0.73	0.85	0.92	0.55	0.91	0.92	0.84
	ASP	0.52	0.75	0.87	0.93	0.57	0.91	0.93	0.85	0.54	0.75	0.88	0.94	0.57	0.91	0.94	0.87
	AT2D	0.53	0.77	0.86	0.92	0.62	0.92	0.92	0.86	0.55	0.77	0.87	0.93	0.62	0.92	0.93	0.88
	DFS	0.50	0.75	0.86	0.92	0.58	0.91	0.92	0.84	0.55	0.75	0.88	0.95	0.56	0.91	0.95	0.88
	ECFP0	0.35	0.72	0.78	0.80	0.63	0.91	0.80	0.80	0.38	0.71	0.80	0.85	0.57	0.91	0.85	0.81
	ECFP2	0.50	0.75	0.86	0.92	0.58	0.91	0.92	0.86	0.53	0.76	0.87	0.93	0.59	0.92	0.93	0.87
	ECFP4	0.52	0.75	0.87	0.93	0.56	0.91	0.93	0.85	0.54	0.75	0.88	0.95	0.56	0.91	0.95	0.88
hERG Liability	ECFP6	0.50	0.73	0.87	0.94	0.53	0.91	0.94	0.84	0.50	0.72	0.87	0.95	0.49	0.90	0.95	0.86
	ESTATE	0.34	0.71	0.77	0.81	0.60	0.91	0.81	0.78	0.33	0.69	0.78	0.83	0.56	0.90	0.83	0.80
	FCFP0	0.19	0.62	0.71	0.76	0.47	0.88	0.76	0.67	0.20	0.63	0.71	0.75	0.51	0.88	0.75	0.68
	FCFP2	0.51	0.76	0.86	0.91	0.62	0.92	0.91	0.86	0.53	0.76	0.87	0.92	0.61	0.92	0.92	0.87
	FCFP4	0.53	0.76	0.87	0.92	0.60	0.92	0.92	0.86	0.54	0.76	0.87	0.93	0.60	0.92	0.93	0.87
	FCFP6	0.50	0.75	0.86	0.92	0.57	0.91	0.92	0.84	0.55	0.75	0.88	0.95	0.55	0.91	0.95	0.88
	KR	0.50	0.75	0.86	0.92	0.58	0.91	0.92	0.86	0.51	0.75	0.86	0.92	0.58	0.91	0.92	0.87
	LSTAR	0.49	0.73	0.86	0.94	0.51	0.90	0.94	0.83	0.50	0.72	0.88	0.96	0.47	0.90	0.96	0.85
	MACCS	0.51	0.76	0.85	0.90	0.62	0.92	0.90	0.86	0.52	0.77	0.86	0.90	0.64	0.92	0.90	0.87
	PUBCHEM	0.52	0.76	0.86	0.92	0.60	0.92	0.92	0.87	0.54	0.76	0.87	0.93	0.59	0.92	0.93	0.88
	RAD2D	0.52	0.76	0.86	0.92	0.59	0.92	0.92	0.85	0.53	0.75	0.87	0.93	0.57	0.91	0.93	0.87
	2PPHAR	0.17	0.66	0.61	0.72	0.59	0.23	0.72	0.68	0.19	0.68	0.63	0.75	0.61	0.24	0.75	0.68
	3PPHAR	0.23	0.65	0.76	0.49	0.81	0.30	0.49	0.72	0.23	0.64	0.77	0.45	0.83	0.30	0.45	0.72
	AP2D	0.33	0.68	0.82	0.48	0.87	0.39	0.48	0.79	0.36	0.69	0.84	0.48	0.89	0.43	0.48	0.80
	ASP	0.49	0.77	0.86	0.65	0.89	0.51	0.65	0.87	0.54	0.78	0.88	0.63	0.93	0.59	0.63	0.89
	AT2D	0.46	0.76	0.85	0.62	0.89	0.49	0.62	0.85	0.46	0.74	0.86	0.58	0.91	0.51	0.58	0.86
	DFS	0.44	0.76	0.84	0.66	0.86	0.45	0.66	0.86	0.46	0.75	0.86	0.60	0.90	0.50	0.60	0.86
	ECFP0	0.34	0.72	0.80	0.61	0.83	0.37	0.61	0.80	0.35	0.72	0.80	0.61	0.83	0.37	0.61	0.81
	ECFP2	0.46	0.75	0.85	0.60	0.90	0.49	0.60	0.85	0.48	0.75	0.87	0.59	0.91	0.54	0.59	0.87
	ECFP4	0.45	0.74	0.85	0.59	0.90	0.49	0.59	0.85	0.46	0.74	0.86	0.58	0.91	0.52	0.58	0.87
Mitochondrial Toxicity	ECFP6	0.42	0.72	0.85	0.53	0.91	0.49	0.53	0.83	0.44	0.70	0.87	0.47	0.94	0.57	0.47	0.84
	ESTATE	0.31	0.70	0.78	0.57	0.82	0.35	0.57	0.78	0.28	0.68	0.78	0.54	0.82	0.33	0.54	0.78
	FCFP0	0.27	0.69	0.74	0.62	0.76	0.31	0.62	0.75	0.30	0.70	0.77	0.61	0.79	0.33	0.61	0.75
	FCFP2	0.44	0.74	0.85	0.60	0.89	0.48	0.60	0.85	0.48	0.76	0.87	0.60	0.91	0.53	0.60	0.86
	FCFP4	0.50	0.76	0.87	0.59	0.92	0.56	0.59	0.86	0.52	0.77	0.88	0.62	0.92	0.56	0.62	0.87
	FCFP6	0.48	0.75	0.87	0.59	0.91	0.54	0.59	0.86	0.47	0.73	0.87	0.53	0.93	0.58	0.53	0.87
	KR	0.48	0.74	0.87	0.57	0.92	0.55	0.57	0.86	0.45	0.72	0.86	0.53	0.92	0.53	0.53	0.86
	LSTAR	0.41	0.73	0.83	0.60	0.87	0.44	0.60	0.83	0.46	0.72	0.88	0.50	0.94	0.57	0.50	0.86

Continued on next page

Table S1 – *Continued from previous page*

Endpoint	FP	Calibration											Validation				
		κ	BACC	ACC	Se	Sp	AUC	κ	BACC	ACC	Se	Sp	AUC				
	MACCS	0.49	0.77	0.86	0.63	0.90	0.52	0.63	0.87	0.54	0.80	0.88	0.69	0.91	0.55	0.69	0.90
	PUBCHEM	0.54	0.79	0.88	0.66	0.91	0.56	0.66	0.90	0.51	0.77	0.87	0.63	0.91	0.55	0.63	0.90
	RAD2D	0.46	0.74	0.86	0.58	0.91	0.52	0.58	0.86	0.43	0.73	0.85	0.56	0.90	0.48	0.56	0.86
	2PPHAR	0.26	0.63	0.65	0.51	0.75	0.58	0.51	0.70	0.30	0.65	0.67	0.57	0.73	0.59	0.57	0.72
	3PPHAR	0.26	0.63	0.64	0.58	0.68	0.55	0.58	0.68	0.30	0.66	0.66	0.66	0.65	0.57	0.66	0.67
	AP2D	0.62	0.80	0.82	0.74	0.87	0.79	0.74	0.87	0.59	0.79	0.80	0.72	0.86	0.78	0.72	0.87
	ASP	0.65	0.83	0.83	0.79	0.86	0.80	0.79	0.89	0.62	0.81	0.82	0.76	0.85	0.78	0.76	0.87
	AT2D	0.62	0.81	0.81	0.80	0.82	0.76	0.80	0.87	0.64	0.83	0.82	0.84	0.81	0.76	0.84	0.88
	DFS	0.64	0.82	0.82	0.77	0.86	0.79	0.77	0.88	0.65	0.82	0.83	0.79	0.86	0.79	0.79	0.89
	ECFP0	0.60	0.80	0.80	0.77	0.83	0.75	0.77	0.87	0.52	0.75	0.77	0.68	0.83	0.73	0.68	0.83
	ECFP2	0.65	0.82	0.83	0.78	0.86	0.80	0.78	0.89	0.65	0.82	0.84	0.77	0.88	0.81	0.77	0.90
	ECFP4	0.64	0.82	0.83	0.76	0.88	0.81	0.76	0.89	0.64	0.82	0.83	0.76	0.88	0.81	0.76	0.88
Respiratory Toxicity	ECFP6	0.63	0.81	0.82	0.76	0.87	0.80	0.76	0.88	0.66	0.83	0.84	0.76	0.90	0.83	0.76	0.90
	ESTATE	0.56	0.78	0.79	0.76	0.81	0.73	0.76	0.84	0.60	0.80	0.80	0.80	0.81	0.74	0.80	0.86
	FCFP0	0.50	0.75	0.76	0.73	0.78	0.69	0.73	0.82	0.52	0.76	0.76	0.75	0.77	0.69	0.75	0.83
	FCFP2	0.61	0.80	0.81	0.76	0.86	0.78	0.76	0.87	0.60	0.80	0.81	0.77	0.83	0.76	0.77	0.88
	FCFP4	0.61	0.81	0.81	0.76	0.85	0.78	0.76	0.89	0.65	0.82	0.83	0.79	0.86	0.79	0.79	0.89
	FCFP6	0.63	0.81	0.82	0.76	0.86	0.79	0.76	0.89	0.62	0.81	0.82	0.77	0.85	0.78	0.77	0.89
	KR	0.61	0.80	0.81	0.75	0.85	0.78	0.75	0.86	0.58	0.78	0.80	0.69	0.87	0.79	0.69	0.87
	LSTAR	0.61	0.80	0.81	0.73	0.87	0.79	0.73	0.86	0.57	0.78	0.80	0.70	0.86	0.78	0.70	0.85
	MACCS	0.65	0.82	0.83	0.78	0.87	0.81	0.78	0.88	0.64	0.82	0.83	0.77	0.86	0.80	0.77	0.88
	PUBCHEM	0.65	0.82	0.83	0.78	0.87	0.80	0.78	0.89	0.69	0.84	0.85	0.80	0.88	0.82	0.80	0.91
	RAD2D	0.67	0.83	0.84	0.79	0.88	0.81	0.79	0.89	0.62	0.80	0.82	0.71	0.90	0.83	0.71	0.89
	2PPHAR	0.06	0.52	0.65	0.15	0.89	0.41	0.15	0.51	0.11	0.55	0.67	0.19	0.91	0.53	0.19	0.51
	3PPHAR	0.07	0.53	0.64	0.21	0.86	0.43	0.21	0.56	-0.05	0.48	0.61	0.09	0.87	0.18	0.09	0.54
	AP2D	0.24	0.61	0.67	0.43	0.80	0.54	0.43	0.66	0.18	0.58	0.65	0.38	0.79	0.49	0.38	0.67
	ASP	0.33	0.66	0.71	0.51	0.81	0.60	0.51	0.71	0.20	0.60	0.66	0.42	0.78	0.49	0.42	0.73
	AT2D	0.30	0.65	0.68	0.59	0.72	0.52	0.59	0.70	0.32	0.66	0.70	0.52	0.79	0.57	0.52	0.70
	DFS	0.35	0.68	0.70	0.64	0.73	0.55	0.64	0.74	0.25	0.63	0.67	0.51	0.75	0.51	0.51	0.74
	ECFP0	0.24	0.61	0.70	0.34	0.88	0.61	0.34	0.62	0.18	0.58	0.69	0.26	0.90	0.56	0.26	0.62
	ECFP2	0.24	0.62	0.67	0.44	0.79	0.52	0.44	0.65	0.25	0.62	0.67	0.46	0.78	0.53	0.46	0.70
	ECFP4	0.28	0.64	0.69	0.47	0.80	0.55	0.47	0.71	0.27	0.62	0.71	0.36	0.88	0.63	0.36	0.70
Toxic Myopathy	ECFP6	0.29	0.65	0.68	0.54	0.76	0.53	0.54	0.70	0.20	0.60	0.64	0.46	0.73	0.46	0.46	0.66
	ESTATE	0.23	0.62	0.65	0.52	0.71	0.48	0.52	0.63	0.23	0.62	0.66	0.49	0.74	0.49	0.49	0.70
	FCFP0	0.22	0.60	0.67	0.42	0.79	0.51	0.42	0.64	0.12	0.56	0.65	0.28	0.84	0.34	0.28	0.58
	FCFP2	0.33	0.66	0.71	0.51	0.81	0.58	0.51	0.69	0.21	0.60	0.65	0.45	0.75	0.48	0.45	0.68
	FCFP4	0.33	0.66	0.71	0.51	0.81	0.59	0.51	0.72	0.31	0.66	0.70	0.54	0.78	0.54	0.54	0.69
	FCFP6	0.27	0.63	0.69	0.46	0.80	0.56	0.46	0.70	0.33	0.66	0.71	0.51	0.81	0.57	0.51	0.77
	KR	0.27	0.63	0.70	0.42	0.84	0.57	0.42	0.67	0.10	0.55	0.65	0.25	0.85	0.44	0.25	0.61
	LSTAR	0.27	0.63	0.69	0.44	0.81	0.56	0.44	0.70	0.25	0.62	0.68	0.44	0.80	0.52	0.44	0.70
	MACCS	0.23	0.61	0.66	0.46	0.76	0.50	0.46	0.66	0.18	0.59	0.61	0.52	0.66	0.45	0.52	0.67
	PUBCHEM	0.19	0.59	0.65	0.43	0.76	0.47	0.43	0.67	0.04	0.51	0.61	0.22	0.81	0.45	0.22	0.63
	RAD2D	0.25	0.62	0.69	0.37	0.86	0.57	0.37	0.67	0.28	0.62	0.72	0.35	0.90	0.65	0.35	0.69
	2PPHAR	0.17	0.58	0.70	0.83	0.33	0.77	0.83	0.61	0.08	0.54	0.66	0.81	0.26	0.75	0.81	0.57
	3PPHAR	0.14	0.56	0.72	0.89	0.23	0.76	0.89	0.66	0.11	0.55	0.69	0.86	0.24	0.76	0.86	0.67
	AP2D	0.34	0.68	0.73	0.80	0.56	0.83	0.80	0.75	0.37	0.68	0.76	0.84	0.52	0.83	0.84	0.76
	ASP	0.40	0.70	0.77	0.84	0.56	0.84	0.84	0.75	0.25	0.61	0.76	0.93	0.28	0.78	0.93	0.72
	AT2D	0.43	0.72	0.77	0.82	0.62	0.86	0.82	0.78	0.52	0.77	0.81	0.85	0.69	0.88	0.85	0.82
	DFS	0.35	0.68	0.74	0.82	0.53	0.83	0.82	0.75	0.34	0.65	0.77	0.91	0.39	0.81	0.91	0.75
	ECFP0	0.34	0.68	0.73	0.79	0.57	0.84	0.79	0.74	0.47	0.74	0.78	0.83	0.65	0.87	0.83	0.78
	ECFP2	0.38	0.68	0.77	0.86	0.51	0.83	0.86	0.77	0.39	0.68	0.79	0.92	0.43	0.82	0.92	0.80
	ECFP4	0.40	0.68	0.79	0.90	0.47	0.82	0.90	0.78	0.51	0.73	0.82	0.92	0.54	0.85	0.92	0.82
	ECFP6	0.40	0.69	0.78	0.88	0.50	0.83	0.88	0.78	0.39	0.68	0.78	0.90	0.45	0.82	0.90	0.78
Myelotoxicity	ESTATE	0.29	0.66	0.70	0.74	0.58	0.83	0.74	0.72	0.29	0.66	0.69	0.71	0.61	0.84	0.71	0.74
	FCFP0	0.17	0.58	0.69	0.82	0.35	0.78	0.82	0.61	0.19	0.58	0.73	0.90	0.26	0.77	0.90	0.60
	FCFP2	0.41	0.71	0.77	0.84	0.58	0.85	0.84	0.77	0.50	0.75	0.80	0.86	0.64	0.87	0.86	0.82
	FCFP4	0.44	0.72	0.78	0.85	0.58	0.85	0.85	0.79	0.44	0.71	0.79	0.88	0.54	0.84	0.88	0.80
	FCFP6	0.41	0.71	0.77	0.83	0.60	0.85	0.83	0.77	0.49	0.73	0.82	0.91	0.54	0.85	0.91	0.83
	KR	0.42	0.71	0.78	0.86	0.56	0.85	0.86	0.79	0.37	0.68	0.76	0.86	0.50	0.83	0.86	0.76
	LSTAR	0.36	0.67	0.77	0.88	0.45	0.82	0.88	0.72	0.34	0.65	0.78	0.93	0.37	0.80	0.93	0.71
	MACCS	0.43	0.73	0.77	0.82	0.63	0.86	0.82	0.80	0.50	0.76	0.80	0.84	0.67	0.88	0.84	0.82
	PUBCHEM	0.42	0.70	0.78	0.86	0.55	0.84	0.86	0.78	0.45	0.72	0.79	0.87	0.56	0.85	0.87	0.80
	RAD2D	0.39	0.68	0.77	0.87	0.49	0.83	0.87	0.76	0.43	0.70	0.80	0.92	0.47	0.83	0.92	0.80
	2PPHAR	0.09	0.54	0.60	0.86	0.22	0.61	0.86	0.58	0.07	0.53	0.58	0.84	0.23	0.61	0.84	0.57
	3PPHAR	0.22	0.60	0.65	0.90	0.30	0.65	0.90	0.67	0.20	0.59	0.64	0.89	0.29	0.64	0.89	0.64
	AP2D	0.29	0.65	0.66	0.70	0.59	0.71	0.70	0.70	0.31	0.65	0.67	0.74	0.57	0.71	0.74	0.72
	ASP	0.36	0.68	0.69	0.74	0.62	0.73	0.74	0.74	0.36	0.68	0.69	0.74	0.62	0.73	0.74	0.73
	AT2D	0.32	0.66	0.66	0.64	0.69	0.75	0.64	0.73	0.33	0.67	0.67	0.66	0.68	0.75	0.66	0.74
	DFS	0.35	0.67	0.68	0.73	0.62	0.74	0.73	0.74	0.33	0.66	0.68	0.79	0.53	0.72	0.79	0.75

Continued on next page

Phototoxicity *in vivo*

Table S1 – *Continued from previous page*

Endpoint	FP	Calibration						Validation									
		κ	BACC	ACC	Se	Sp	AUC	κ	BACC	ACC	Se	Sp	AUC				
	ECFP0	0.27	0.64	0.64	0.64	0.63	0.71	0.64	0.68	0.29	0.65	0.65	0.69	0.61	0.72	0.69	0.68
	ECFP2	0.34	0.67	0.68	0.75	0.59	0.72	0.75	0.72	0.29	0.64	0.66	0.74	0.54	0.70	0.74	0.72
	ECFP4	0.33	0.66	0.68	0.77	0.56	0.71	0.77	0.72	0.41	0.70	0.72	0.82	0.58	0.73	0.82	0.76
	ECFP6	0.31	0.65	0.67	0.76	0.55	0.70	0.76	0.70	0.31	0.65	0.67	0.79	0.51	0.70	0.79	0.72
	ESTATE	0.27	0.64	0.64	0.64	0.63	0.71	0.64	0.68	0.29	0.65	0.65	0.64	0.65	0.72	0.64	0.69
	FCFP0	0.19	0.59	0.61	0.72	0.47	0.66	0.72	0.62	0.17	0.59	0.60	0.68	0.49	0.66	0.68	0.62
	FCFP2	0.36	0.68	0.69	0.75	0.61	0.73	0.75	0.74	0.35	0.68	0.68	0.72	0.63	0.73	0.72	0.72
	FCFP4	0.37	0.68	0.70	0.81	0.54	0.72	0.81	0.74	0.36	0.67	0.70	0.83	0.52	0.71	0.83	0.74
	FCFP6	0.36	0.68	0.69	0.73	0.62	0.73	0.73	0.74	0.42	0.71	0.72	0.79	0.63	0.75	0.79	0.78
	KR	0.36	0.68	0.70	0.82	0.53	0.71	0.82	0.74	0.36	0.68	0.70	0.80	0.55	0.72	0.80	0.75
	LSTAR	0.31	0.65	0.67	0.75	0.56	0.71	0.75	0.72	0.31	0.65	0.67	0.75	0.56	0.71	0.75	0.71
	MACCS	0.35	0.68	0.69	0.75	0.61	0.73	0.75	0.73	0.34	0.67	0.69	0.76	0.58	0.72	0.76	0.76
	PUBCHEM	0.38	0.69	0.70	0.76	0.62	0.74	0.76	0.75	0.34	0.67	0.68	0.76	0.58	0.72	0.76	0.75
	RAD2D	0.33	0.66	0.68	0.80	0.52	0.70	0.80	0.71	0.42	0.70	0.73	0.88	0.52	0.72	0.88	0.76
	2PPHAR	0.11	0.55	0.60	0.89	0.21	0.60	0.89	0.60	0.05	0.52	0.58	0.90	0.15	0.59	0.90	0.64
	3PPHAR	0.17	0.58	0.60	0.72	0.44	0.64	0.72	0.64	0.11	0.55	0.58	0.72	0.39	0.61	0.72	0.62
	AP2D	0.27	0.64	0.64	0.67	0.60	0.70	0.67	0.68	0.19	0.60	0.60	0.65	0.54	0.66	0.65	0.67
	ASP	0.35	0.68	0.68	0.68	0.68	0.74	0.68	0.72	0.38	0.69	0.69	0.69	0.70	0.75	0.69	0.78
	AT2D	0.31	0.66	0.65	0.60	0.71	0.74	0.60	0.74	0.21	0.61	0.60	0.58	0.63	0.68	0.58	0.69
	DFS	0.30	0.65	0.65	0.66	0.65	0.72	0.66	0.72	0.42	0.71	0.71	0.70	0.73	0.78	0.70	0.76
	ECFP0	0.35	0.68	0.68	0.69	0.67	0.74	0.69	0.73	0.13	0.57	0.57	0.61	0.53	0.63	0.61	0.65
	ECFP2	0.33	0.66	0.68	0.75	0.58	0.71	0.75	0.74	0.40	0.70	0.71	0.78	0.61	0.73	0.78	0.79
	ECFP4	0.38	0.69	0.69	0.73	0.65	0.74	0.73	0.75	0.35	0.68	0.68	0.67	0.69	0.75	0.67	0.77
Phototoxicity *in vitro*	ECFP6	0.37	0.69	0.69	0.69	0.69	0.75	0.69	0.75	0.31	0.65	0.66	0.69	0.62	0.71	0.69	0.71
	ESTATE	0.38	0.69	0.70	0.76	0.62	0.73	0.76	0.74	0.31	0.65	0.67	0.74	0.56	0.70	0.74	0.72
	FCFP0	0.19	0.59	0.61	0.70	0.49	0.65	0.70	0.62	0.09	0.54	0.57	0.71	0.38	0.61	0.71	0.57
	FCFP2	0.37	0.69	0.69	0.72	0.66	0.74	0.72	0.74	0.40	0.70	0.70	0.73	0.67	0.74	0.73	0.79
	FCFP4	0.35	0.67	0.69	0.77	0.58	0.71	0.77	0.74	0.36	0.68	0.69	0.75	0.61	0.72	0.75	0.74
	FCFP6	0.28	0.63	0.65	0.76	0.51	0.68	0.76	0.71	0.28	0.64	0.66	0.75	0.53	0.68	0.75	0.70
	KR	0.42	0.70	0.72	0.82	0.59	0.73	0.82	0.76	0.39	0.69	0.71	0.80	0.59	0.72	0.80	0.80
	LSTAR	0.29	0.65	0.66	0.72	0.57	0.70	0.72	0.72	0.26	0.63	0.64	0.69	0.57	0.68	0.69	0.67
	MACCS	0.41	0.71	0.71	0.71	0.71	0.77	0.71	0.78	0.27	0.63	0.64	0.67	0.60	0.69	0.67	0.72
	PUBCHEM	0.38	0.69	0.70	0.74	0.65	0.74	0.74	0.78	0.44	0.72	0.73	0.78	0.65	0.75	0.78	0.82
	RAD2D	0.30	0.64	0.67	0.81	0.48	0.68	0.81	0.70	0.29	0.64	0.68	0.92	0.36	0.66	0.92	0.76
	2PPHAR	0.01	0.51	0.64	0.90	0.12	0.68	0.90	0.54	0.00	0.50	0.64	0.90	0.09	0.68	0.90	0.53
	3PPHAR	0.18	0.57	0.71	0.96	0.19	0.71	0.96	0.66	0.18	0.57	0.71	0.96	0.18	0.71	0.96	0.67
	AP2D	0.39	0.68	0.75	0.87	0.50	0.78	0.87	0.77	0.39	0.68	0.75	0.88	0.48	0.78	0.88	0.78
	ASP	0.57	0.78	0.82	0.89	0.67	0.85	0.89	0.86	0.58	0.78	0.82	0.90	0.66	0.85	0.90	0.87
	AT2D	0.56	0.78	0.81	0.86	0.69	0.85	0.86	0.85	0.56	0.78	0.81	0.86	0.71	0.86	0.86	0.85
	DFS	0.55	0.77	0.81	0.86	0.68	0.85	0.86	0.85	0.56	0.77	0.81	0.88	0.67	0.85	0.88	0.87
	ECFP0	0.39	0.69	0.74	0.82	0.57	0.80	0.82	0.77	0.41	0.69	0.75	0.85	0.54	0.79	0.85	0.78
	ECFP2	0.57	0.78	0.81	0.89	0.67	0.85	0.89	0.86	0.57	0.78	0.82	0.89	0.66	0.85	0.89	0.87
	ECFP4	0.55	0.76	0.81	0.91	0.62	0.83	0.91	0.86	0.61	0.79	0.84	0.92	0.66	0.85	0.92	0.89
Cytotoxicity (HepG2)	ECFP6	0.55	0.76	0.81	0.90	0.63	0.84	0.90	0.86	0.58	0.78	0.83	0.92	0.63	0.84	0.92	0.87
	ESTATE	0.39	0.69	0.74	0.82	0.56	0.80	0.82	0.76	0.40	0.69	0.74	0.84	0.55	0.80	0.84	0.78
	FCFP0	0.01	0.50	0.67	0.97	0.04	0.68	0.97	0.52	0.01	0.51	0.67	0.98	0.03	0.68	0.98	0.50
	FCFP2	0.53	0.76	0.80	0.87	0.65	0.84	0.87	0.84	0.55	0.77	0.81	0.87	0.68	0.85	0.87	0.86
	FCFP4	0.56	0.77	0.81	0.88	0.67	0.85	0.88	0.86	0.59	0.78	0.83	0.91	0.65	0.85	0.91	0.88
	FCFP6	0.57	0.78	0.82	0.90	0.66	0.85	0.90	0.86	0.55	0.77	0.81	0.89	0.65	0.84	0.89	0.87
	KR	0.54	0.76	0.81	0.89	0.64	0.84	0.89	0.84	0.55	0.77	0.81	0.89	0.64	0.84	0.89	0.87
	LSTAR	0.53	0.74	0.81	0.93	0.55	0.81	0.93	0.85	0.55	0.75	0.82	0.94	0.56	0.82	0.94	0.86
	MACCS	0.52	0.76	0.80	0.87	0.64	0.84	0.87	0.84	0.55	0.77	0.81	0.89	0.65	0.84	0.89	0.85
	PUBCHEM	0.54	0.76	0.80	0.87	0.65	0.84	0.87	0.85	0.58	0.78	0.82	0.89	0.67	0.85	0.89	0.87
	RAD2D	0.56	0.77	0.81	0.89	0.66	0.84	0.89	0.86	0.57	0.77	0.82	0.92	0.62	0.83	0.92	0.87
	2PPHAR	0.12	0.65	0.57	0.75	0.55	0.17	0.75	0.67	0.11	0.64	0.57	0.72	0.56	0.16	0.72	0.65
	3PPHAR	0.15	0.58	0.83	0.25	0.90	0.24	0.25	0.68	0.14	0.57	0.84	0.22	0.92	0.24	0.22	0.66
	AP2D	0.31	0.70	0.83	0.54	0.86	0.32	0.54	0.78	0.36	0.71	0.86	0.52	0.90	0.38	0.52	0.80
	ASP	0.54	0.78	0.91	0.62	0.94	0.57	0.62	0.88	0.58	0.78	0.92	0.60	0.96	0.64	0.60	0.89
	AT2D	0.49	0.79	0.88	0.67	0.91	0.47	0.67	0.87	0.48	0.78	0.88	0.65	0.91	0.47	0.65	0.86
	DFS	0.50	0.78	0.90	0.63	0.93	0.51	0.63	0.88	0.51	0.76	0.90	0.58	0.94	0.57	0.58	0.87
	ECFP0	0.30	0.71	0.81	0.59	0.83	0.30	0.59	0.79	0.32	0.71	0.83	0.56	0.86	0.32	0.56	0.79
	ECFP2	0.51	0.78	0.90	0.63	0.93	0.51	0.63	0.87	0.55	0.79	0.91	0.63	0.95	0.58	0.63	0.89
Cytotoxicity (CRL-7250 cell line)	ECFP4	0.51	0.77	0.90	0.59	0.94	0.55	0.59	0.87	0.55	0.74	0.92	0.52	0.97	0.68	0.52	0.87
	ECFP6	0.47	0.74	0.90	0.53	0.94	0.53	0.53	0.86	0.47	0.72	0.91	0.48	0.96	0.57	0.48	0.85
	ESTATE	0.26	0.68	0.80	0.52	0.84	0.28	0.52	0.76	0.23	0.65	0.81	0.44	0.86	0.27	0.44	0.74
	FCFP0	0.21	0.64	0.80	0.42	0.85	0.26	0.42	0.69	0.21	0.63	0.81	0.40	0.86	0.26	0.40	0.69
	FCFP2	0.44	0.75	0.88	0.60	0.91	0.44	0.60	0.84	0.48	0.77	0.89	0.61	0.92	0.49	0.61	0.86
	FCFP4	0.48	0.76	0.89	0.60	0.93	0.50	0.60	0.86	0.55	0.76	0.92	0.55	0.96	0.64	0.55	0.89
	FCFP6	0.49	0.74	0.90	0.55	0.94	0.54	0.55	0.86	0.48	0.71	0.91	0.46	0.97	0.62	0.46	0.87

Continued on next page

Table S1 – *Continued from previous page*

Endpoint	FP	Calibration						Validation									
		κ	BACC	ACC	Se	Sp	AUC	κ	BACC	ACC	Se	Sp	AUC				
	KR	0.49	0.76	0.89	0.59	0.93	0.51	0.59	0.86	0.50	0.77	0.90	0.60	0.94	0.53	0.60	0.86
	LSTAR	0.49	0.73	0.91	0.51	0.96	0.59	0.51	0.85	0.50	0.70	0.92	0.42	0.98	0.74	0.42	0.86
	MACCS	0.44	0.77	0.87	0.64	0.90	0.43	0.64	0.87	0.46	0.77	0.88	0.65	0.90	0.45	0.65	0.88
	PUBCHEM	0.47	0.78	0.88	0.66	0.91	0.46	0.66	0.88	0.50	0.81	0.88	0.73	0.90	0.47	0.73	0.90
	RAD2D	0.49	0.76	0.90	0.59	0.93	0.52	0.59	0.86	0.55	0.77	0.92	0.58	0.96	0.62	0.58	0.88
Cytotoxicity (HACAT cell line)	2PPHAR	0.11	0.63	0.60	0.67	0.60	0.16	0.67	0.65	0.12	0.64	0.58	0.72	0.56	0.17	0.72	0.65
	3PPHAR	0.16	0.59	0.82	0.29	0.88	0.24	0.29	0.65	0.16	0.58	0.83	0.25	0.91	0.24	0.25	0.65
	AP2D	0.32	0.71	0.83	0.55	0.86	0.33	0.55	0.79	0.34	0.70	0.85	0.52	0.89	0.36	0.52	0.78
	ASP	0.53	0.77	0.91	0.59	0.95	0.58	0.59	0.87	0.55	0.75	0.92	0.54	0.96	0.66	0.54	0.86
	AT2D	0.45	0.77	0.87	0.65	0.90	0.44	0.65	0.85	0.44	0.77	0.87	0.64	0.90	0.43	0.64	0.85
	DFS	0.50	0.76	0.90	0.58	0.94	0.53	0.58	0.85	0.49	0.77	0.89	0.61	0.93	0.51	0.61	0.86
	ECFP0	0.31	0.71	0.82	0.56	0.85	0.31	0.56	0.78	0.34	0.73	0.83	0.59	0.86	0.34	0.59	0.79
	ECFP2	0.47	0.77	0.88	0.63	0.91	0.47	0.63	0.84	0.50	0.75	0.90	0.56	0.95	0.56	0.56	0.86
	ECFP4	0.49	0.76	0.90	0.58	0.93	0.53	0.58	0.85	0.49	0.72	0.91	0.47	0.97	0.63	0.47	0.84
	ECFP6	0.48	0.72	0.91	0.49	0.96	0.59	0.49	0.84	0.43	0.70	0.90	0.44	0.95	0.54	0.44	0.83
	ESTATE	0.26	0.68	0.80	0.52	0.84	0.28	0.52	0.75	0.26	0.68	0.81	0.50	0.85	0.29	0.50	0.75
	FCFP0	0.26	0.65	0.84	0.41	0.89	0.32	0.41	0.69	0.27	0.65	0.84	0.41	0.90	0.32	0.41	0.71
	FCFP2	0.43	0.75	0.87	0.61	0.90	0.43	0.61	0.84	0.42	0.74	0.87	0.58	0.91	0.44	0.58	0.83
	FCFP4	0.51	0.76	0.90	0.57	0.94	0.56	0.57	0.85	0.52	0.73	0.92	0.49	0.97	0.66	0.49	0.85
	FCFP6	0.46	0.74	0.89	0.55	0.93	0.50	0.55	0.83	0.50	0.72	0.92	0.46	0.97	0.68	0.46	0.84
	KR	0.46	0.75	0.89	0.58	0.92	0.48	0.58	0.83	0.50	0.75	0.90	0.56	0.94	0.55	0.56	0.85
	LSTAR	0.46	0.72	0.90	0.48	0.95	0.56	0.48	0.83	0.50	0.70	0.92	0.43	0.98	0.73	0.43	0.84
	MACCS	0.43	0.76	0.87	0.62	0.90	0.42	0.62	0.84	0.45	0.77	0.87	0.65	0.90	0.44	0.65	0.88
	PUBCHEM	0.47	0.77	0.88	0.63	0.91	0.46	0.63	0.86	0.47	0.76	0.89	0.59	0.92	0.49	0.59	0.85
	RAD2D	0.50	0.76	0.90	0.58	0.94	0.54	0.58	0.85	0.53	0.76	0.91	0.57	0.95	0.59	0.57	0.87
Cytotoxicity (HEK cell line)	2PPHAR	0.15	0.65	0.59	0.74	0.56	0.22	0.74	0.66	0.14	0.64	0.59	0.71	0.57	0.21	0.71	0.65
	3PPHAR	0.16	0.57	0.81	0.24	0.91	0.29	0.24	0.66	0.15	0.57	0.83	0.21	0.93	0.31	0.21	0.67
	AP2D	0.35	0.70	0.83	0.51	0.88	0.40	0.51	0.78	0.37	0.70	0.83	0.52	0.88	0.42	0.52	0.79
	ASP	0.51	0.75	0.88	0.58	0.93	0.58	0.58	0.85	0.57	0.78	0.90	0.60	0.95	0.65	0.60	0.88
	AT2D	0.46	0.75	0.85	0.62	0.89	0.48	0.62	0.84	0.51	0.78	0.87	0.66	0.91	0.53	0.66	0.86
	DFS	0.47	0.75	0.87	0.59	0.91	0.52	0.59	0.84	0.50	0.75	0.88	0.57	0.93	0.59	0.57	0.86
	ECFP0	0.30	0.69	0.79	0.55	0.83	0.34	0.55	0.77	0.32	0.69	0.80	0.54	0.84	0.36	0.54	0.77
	ECFP2	0.48	0.75	0.87	0.59	0.91	0.52	0.59	0.84	0.52	0.76	0.89	0.58	0.94	0.60	0.58	0.86
	ECFP4	0.50	0.74	0.88	0.54	0.94	0.59	0.54	0.85	0.49	0.71	0.90	0.45	0.97	0.71	0.45	0.85
	ECFP6	0.46	0.72	0.87	0.51	0.93	0.56	0.51	0.83	0.45	0.70	0.88	0.45	0.95	0.60	0.45	0.83
	ESTATE	0.28	0.68	0.78	0.53	0.82	0.33	0.53	0.75	0.24	0.65	0.78	0.48	0.83	0.31	0.48	0.73
	FCFP0	0.24	0.63	0.80	0.38	0.87	0.33	0.38	0.68	0.23	0.62	0.80	0.36	0.88	0.32	0.36	0.66
	FCFP2	0.44	0.74	0.86	0.57	0.90	0.49	0.57	0.83	0.43	0.72	0.86	0.52	0.92	0.51	0.52	0.82
	FCFP4	0.48	0.74	0.88	0.55	0.93	0.56	0.55	0.85	0.49	0.73	0.88	0.52	0.94	0.60	0.52	0.85
	FCFP6	0.43	0.71	0.87	0.48	0.93	0.54	0.48	0.82	0.49	0.70	0.90	0.43	0.97	0.72	0.43	0.86
	KR	0.45	0.73	0.87	0.53	0.92	0.53	0.53	0.83	0.48	0.73	0.88	0.52	0.94	0.59	0.52	0.85
	LSTAR	0.44	0.70	0.88	0.46	0.94	0.58	0.46	0.82	0.51	0.72	0.90	0.46	0.97	0.73	0.46	0.87
	MACCS	0.46	0.76	0.85	0.62	0.89	0.49	0.62	0.85	0.50	0.77	0.87	0.63	0.91	0.53	0.63	0.87
	PUBCHEM	0.50	0.77	0.87	0.62	0.91	0.54	0.62	0.86	0.49	0.76	0.87	0.62	0.91	0.52	0.62	0.86
	RAD2D	0.47	0.73	0.87	0.55	0.92	0.54	0.55	0.84	0.49	0.72	0.89	0.48	0.96	0.65	0.48	0.86
Cytotoxicity (NIK cell line)	2PPHAR	0.10	0.63	0.58	0.69	0.57	0.16	0.69	0.64	0.11	0.64	0.57	0.74	0.55	0.16	0.74	0.66
	3PPHAR	0.16	0.59	0.82	0.31	0.88	0.23	0.31	0.67	0.15	0.58	0.83	0.26	0.90	0.25	0.26	0.67
	AP2D	0.37	0.74	0.85	0.59	0.88	0.37	0.59	0.82	0.40	0.74	0.86	0.60	0.89	0.40	0.60	0.82
	ASP	0.49	0.77	0.90	0.61	0.93	0.51	0.61	0.87	0.57	0.76	0.92	0.55	0.97	0.68	0.55	0.89
	AT2D	0.45	0.78	0.87	0.66	0.90	0.43	0.66	0.86	0.49	0.81	0.88	0.72	0.90	0.45	0.72	0.90
	DFS	0.49	0.77	0.89	0.61	0.93	0.50	0.61	0.87	0.48	0.74	0.90	0.55	0.94	0.53	0.55	0.86
	ECFP0	0.30	0.72	0.80	0.62	0.82	0.30	0.62	0.79	0.35	0.73	0.84	0.60	0.87	0.35	0.60	0.82
	ECFP2	0.50	0.78	0.89	0.64	0.92	0.50	0.64	0.88	0.53	0.78	0.91	0.61	0.94	0.55	0.61	0.87
	ECFP4	0.52	0.76	0.91	0.57	0.95	0.57	0.57	0.87	0.54	0.75	0.92	0.53	0.97	0.65	0.53	0.90
	ECFP6	0.44	0.73	0.88	0.53	0.93	0.48	0.53	0.84	0.50	0.73	0.91	0.50	0.96	0.62	0.50	0.87
	ESTATE	0.29	0.71	0.81	0.57	0.84	0.30	0.57	0.77	0.31	0.71	0.82	0.57	0.85	0.31	0.57	0.80
	FCFP0	0.23	0.66	0.79	0.49	0.83	0.26	0.49	0.72	0.29	0.67	0.84	0.47	0.88	0.31	0.47	0.74
	FCFP2	0.43	0.76	0.87	0.61	0.90	0.42	0.61	0.85	0.49	0.78	0.89	0.65	0.92	0.49	0.65	0.87
	FCFP4	0.45	0.76	0.88	0.61	0.91	0.45	0.61	0.85	0.55	0.76	0.92	0.56	0.96	0.64	0.56	0.88
	FCFP6	0.46	0.73	0.90	0.53	0.94	0.52	0.53	0.85	0.50	0.71	0.92	0.43	0.98	0.71	0.43	0.86
	KR	0.48	0.76	0.89	0.59	0.93	0.49	0.59	0.86	0.48	0.75	0.90	0.56	0.94	0.52	0.56	0.86
	LSTAR	0.44	0.72	0.89	0.50	0.94	0.51	0.50	0.85	0.45	0.67	0.92	0.36	0.99	0.81	0.36	0.85
	MACCS	0.44	0.77	0.87	0.64	0.90	0.43	0.64	0.86	0.48	0.77	0.89	0.62	0.92	0.48	0.62	0.87
	PUBCHEM	0.47	0.78	0.88	0.66	0.91	0.46	0.66	0.87	0.49	0.78	0.89	0.64	0.92	0.49	0.64	0.87
	RAD2D	0.48	0.76	0.89	0.60	0.93	0.49	0.60	0.86	0.51	0.76	0.91	0.57	0.95	0.56	0.57	0.87
Carcinogenicity	2PPHAR	0.05	0.52	0.53	0.19	0.86	0.59	0.19	0.58	-0.04	0.48	0.49	0.07	0.89	0.37	0.07	0.48
	3PPHAR	0.02	0.51	0.52	0.11	0.91	0.54	0.11	0.56	0.02	0.51	0.52	0.12	0.90	0.53	0.12	0.56
	AP2D	0.26	0.63	0.63	0.54	0.72	0.66	0.54	0.66	0.23	0.62	0.62	0.49	0.74	0.65	0.49	0.66
	ASP	0.28	0.64	0.64	0.50	0.78	0.69	0.50	0.69	0.28	0.64	0.64	0.50	0.77	0.68	0.50	0.68

Continued on next page

Table S1 – *Continued from previous page*

Endpoint	FP	Calibration						Validation									
		κ	BACC	ACC	Se	Sp	AUC	κ	BACC	ACC	Se	Sp	AUC				
	AT2D	0.26	0.63	0.63	0.70	0.56	0.61	0.70	0.69	0.18	0.59	0.59	0.64	0.55	0.58	0.64	0.66
	DFS	0.27	0.64	0.64	0.59	0.68	0.64	0.59	0.68	0.20	0.60	0.60	0.41	0.78	0.65	0.41	0.67
	ECFP0	0.28	0.64	0.64	0.59	0.69	0.65	0.59	0.69	0.28	0.64	0.64	0.59	0.69	0.64	0.59	0.67
	ECFP2	0.30	0.65	0.65	0.57	0.73	0.68	0.57	0.69	0.25	0.62	0.63	0.40	0.84	0.71	0.40	0.69
	ECFP4	0.28	0.64	0.64	0.53	0.75	0.68	0.53	0.68	0.32	0.66	0.66	0.51	0.81	0.73	0.51	0.71
	ECFP6	0.27	0.64	0.64	0.53	0.74	0.67	0.53	0.68	0.26	0.63	0.63	0.53	0.73	0.66	0.53	0.67
	ESTATE	0.26	0.63	0.63	0.63	0.63	0.63	0.63	0.67	0.27	0.64	0.64	0.62	0.65	0.64	0.62	0.68
	FCFP0	0.16	0.58	0.58	0.62	0.54	0.56	0.62	0.59	0.14	0.57	0.57	0.59	0.55	0.57	0.59	0.60
	FCFP2	0.29	0.64	0.64	0.49	0.79	0.70	0.49	0.69	0.31	0.66	0.66	0.50	0.81	0.72	0.50	0.71
	FCFP4	0.31	0.66	0.66	0.54	0.77	0.70	0.54	0.71	0.31	0.65	0.65	0.51	0.79	0.71	0.51	0.71
	FCFP6	0.31	0.65	0.66	0.52	0.79	0.71	0.52	0.69	0.29	0.65	0.65	0.50	0.79	0.70	0.50	0.68
	KR	0.32	0.66	0.66	0.54	0.78	0.70	0.54	0.70	0.29	0.64	0.64	0.53	0.75	0.68	0.53	0.70
	LSTAR	0.27	0.63	0.64	0.53	0.74	0.67	0.53	0.66	0.28	0.64	0.64	0.58	0.69	0.65	0.58	0.68
	MACCS	0.33	0.67	0.67	0.66	0.68	0.66	0.66	0.71	0.32	0.66	0.66	0.49	0.83	0.74	0.49	0.70
	PUBCHEM	0.34	0.67	0.67	0.58	0.76	0.70	0.58	0.71	0.36	0.68	0.68	0.56	0.80	0.73	0.56	0.75
	RAD2D	0.29	0.64	0.65	0.52	0.77	0.70	0.52	0.68	0.29	0.65	0.65	0.45	0.84	0.76	0.45	0.72
Ototoxicity	2PPHAR	0.10	0.54	0.66	0.91	0.17	0.68	0.91	0.57	0.06	0.52	0.64	0.90	0.15	0.67	0.90	0.55
	3PPHAR	0.13	0.55	0.67	0.91	0.20	0.68	0.91	0.60	0.06	0.52	0.66	0.95	0.10	0.67	0.95	0.56
	AP2D	0.29	0.64	0.69	0.81	0.47	0.75	0.81	0.69	0.31	0.64	0.72	0.90	0.38	0.74	0.90	0.71
	ASP	0.38	0.68	0.73	0.85	0.51	0.77	0.85	0.71	0.39	0.69	0.74	0.85	0.52	0.77	0.85	0.73
	AT2D	0.39	0.69	0.73	0.83	0.55	0.78	0.83	0.73	0.33	0.66	0.71	0.81	0.51	0.76	0.81	0.71
	DFS	0.38	0.68	0.73	0.85	0.51	0.77	0.85	0.72	0.35	0.67	0.73	0.86	0.48	0.76	0.86	0.73
	ECFP0	0.31	0.65	0.71	0.83	0.46	0.75	0.83	0.69	0.37	0.68	0.73	0.85	0.51	0.77	0.85	0.72
	ECFP2	0.37	0.67	0.74	0.90	0.44	0.75	0.90	0.74	0.40	0.68	0.75	0.91	0.45	0.76	0.91	0.76
	ECFP4	0.36	0.67	0.73	0.85	0.48	0.76	0.85	0.73	0.37	0.66	0.75	0.96	0.36	0.74	0.96	0.75
	ECFP6	0.38	0.68	0.73	0.85	0.51	0.77	0.85	0.73	0.40	0.68	0.76	0.92	0.44	0.76	0.92	0.73
	ESTATE	0.27	0.63	0.68	0.77	0.49	0.74	0.77	0.68	0.30	0.64	0.70	0.84	0.44	0.74	0.84	0.69
	FCFP0	0.16	0.57	0.66	0.85	0.29	0.70	0.85	0.61	0.14	0.56	0.66	0.87	0.25	0.69	0.87	0.61
	FCFP2	0.33	0.65	0.71	0.84	0.46	0.75	0.84	0.71	0.36	0.66	0.74	0.89	0.44	0.75	0.89	0.75
	FCFP4	0.36	0.66	0.73	0.89	0.44	0.75	0.89	0.73	0.36	0.66	0.74	0.91	0.41	0.75	0.91	0.72
	FCFP6	0.37	0.68	0.73	0.86	0.50	0.77	0.86	0.73	0.36	0.66	0.74	0.93	0.39	0.74	0.93	0.73
	KR	0.33	0.65	0.71	0.84	0.47	0.75	0.84	0.72	0.33	0.65	0.74	0.93	0.36	0.74	0.93	0.72
	LSTAR	0.35	0.66	0.73	0.87	0.46	0.75	0.87	0.72	0.33	0.65	0.73	0.91	0.38	0.74	0.91	0.71
	MACCS	0.35	0.67	0.72	0.82	0.52	0.77	0.82	0.71	0.27	0.61	0.72	0.94	0.29	0.72	0.94	0.69
	PUBCHEM	0.40	0.69	0.74	0.86	0.52	0.77	0.86	0.74	0.36	0.67	0.73	0.86	0.48	0.76	0.86	0.72
	RAD2D	0.33	0.65	0.72	0.86	0.45	0.75	0.86	0.71	0.34	0.65	0.74	0.94	0.36	0.74	0.94	0.71
Urinary Tract toxicity	2PPHAR	0.09	0.54	0.59	0.33	0.76	0.46	0.33	0.57	0.13	0.56	0.64	0.17	0.95	NA	0.17	0.54
	3PPHAR	0.04	0.52	0.58	0.26	0.78	0.43	0.26	0.51	-0.02	0.49	0.54	0.25	0.73	0.38	0.25	0.51
	AP2D	0.26	0.63	0.65	0.52	0.74	0.56	0.52	0.68	0.12	0.56	0.59	0.44	0.68	0.47	0.44	0.66
	ASP	0.38	0.69	0.71	0.62	0.77	0.62	0.62	0.75	0.19	0.59	0.61	0.52	0.67	0.50	0.52	0.71
	AT2D	0.34	0.68	0.67	0.70	0.65	0.57	0.70	0.74	0.21	0.61	0.63	0.52	0.69	0.52	0.52	0.69
	DFS	0.36	0.68	0.69	0.66	0.71	0.60	0.66	0.74	0.41	0.70	0.72	0.65	0.76	0.65	0.65	0.76
	ECFP0	0.16	0.58	0.59	0.54	0.62	0.48	0.54	0.61	0.26	0.63	0.65	0.52	0.73	0.56	0.52	0.72
	ECFP2	0.32	0.66	0.68	0.57	0.75	0.60	0.57	0.71	0.40	0.69	0.72	0.54	0.84	0.69	0.54	0.75
	ECFP4	0.28	0.64	0.66	0.55	0.73	0.57	0.55	0.70	0.28	0.64	0.66	0.54	0.73	0.57	0.54	0.74
	ECFP6	0.26	0.63	0.65	0.53	0.73	0.55	0.53	0.69	0.36	0.68	0.69	0.65	0.72	0.59	0.65	0.76
	ESTATE	0.24	0.62	0.63	0.60	0.65	0.53	0.60	0.65	0.23	0.61	0.63	0.52	0.71	0.54	0.52	0.63
	FCFP0	0.24	0.62	0.62	0.62	0.62	0.51	0.62	0.65	0.29	0.64	0.66	0.58	0.71	0.57	0.58	0.69
	FCFP2	0.34	0.67	0.69	0.58	0.76	0.61	0.58	0.71	0.38	0.69	0.71	0.62	0.76	0.62	0.62	0.81
	FCFP4	0.41	0.71	0.72	0.66	0.75	0.64	0.66	0.77	0.38	0.70	0.69	0.75	0.65	0.59	0.75	0.73
	FCFP6	0.39	0.70	0.71	0.62	0.77	0.64	0.62	0.78	0.30	0.65	0.67	0.52	0.77	0.60	0.52	0.74
	KR	0.38	0.68	0.71	0.54	0.82	0.67	0.54	0.77	0.41	0.70	0.72	0.58	0.81	0.70	0.58	0.78
	LSTAR	0.34	0.66	0.70	0.51	0.82	0.64	0.51	0.75	0.45	0.73	0.74	0.71	0.76	0.63	0.71	0.81
	MACCS	0.42	0.71	0.72	0.64	0.77	0.65	0.64	0.76	0.24	0.62	0.63	0.54	0.69	0.54	0.54	0.71
	PUBCHEM	0.38	0.69	0.70	0.61	0.76	0.63	0.61	0.74	0.32	0.65	0.70	0.44	0.87	0.68	0.44	0.73
	RAD2D	0.33	0.66	0.69	0.55	0.78	0.63	0.55	0.74	0.32	0.66	0.69	0.50	0.81	0.63	0.50	0.78
Rat acute oral toxicity (LD50)	2PPHAR	0.08	0.56	0.38	0.35	0.77	0.33	0.35	0.59	0.09	0.56	0.39	0.34	0.77	0.36	0.34	0.61
	3PPHAR	0.10	0.56	0.41	0.35	0.78	0.34	0.35	0.60	0.13	0.58	0.42	0.38	0.78	0.38	0.38	0.64
	AP2D	0.27	0.66	0.52	0.50	0.81	0.47	0.50	0.73	0.26	0.65	0.52	0.49	0.81	0.47	0.49	0.76
	ASP	0.29	0.66	0.55	0.51	0.82	0.52	0.51	0.75	0.31	0.67	0.56	0.52	0.82	0.53	0.52	0.79
	AT2D	0.29	0.66	0.56	0.50	0.82	0.53	0.50	0.75	0.31	0.67	0.57	0.52	0.82	0.53	0.52	0.78
	DFS	0.29	0.66	0.55	0.50	0.82	0.52	0.50	0.75	0.32	0.67	0.57	0.52	0.82	0.55	0.52	0.79
	ECFP0	0.25	0.65	0.49	0.49	0.81	0.45	0.49	0.72	0.25	0.65	0.49	0.49	0.81	0.44	0.49	0.75
	ECFP2	0.31	0.67	0.57	0.52	0.82	0.53	0.52	0.77	0.32	0.68	0.57	0.53	0.83	0.53	0.53	0.79
	ECFP4	0.29	0.66	0.55	0.50	0.82	0.52	0.50	0.75	0.31	0.67	0.56	0.52	0.82	0.54	0.52	0.80
	ECFP6	0.28	0.66	0.55	0.49	0.82	0.54	0.49	0.74	0.31	0.67	0.57	0.52	0.82	0.55	0.52	0.79
	ESTATE	0.24	0.65	0.49	0.48	0.81	0.44	0.48	0.73	0.20	0.62	0.45	0.45	0.80	0.41	0.45	0.74
	FCFP0	0.08	0.56	0.29	0.34	0.77	0.41	0.34	0.62	0.10	0.57	0.32	0.36	0.78	0.43	0.36	0.65
	FCFP2	0.29	0.66	0.54	0.50	0.82	0.50	0.50	0.76	0.28	0.66	0.54	0.51	0.81	0.51	0.51	0.78

Continued on next page

Table S1 – *Continued from previous page*

Endpoint	FP	Calibration						Validation									
		κ	BACC	ACC	Se	Sp	AUC	κ	BACC	ACC	Se	Sp	AUC				
	FCFP4	0.29	0.66	0.55	0.51	0.82	0.51	0.51	0.76	0.31	0.67	0.56	0.52	0.82	0.52	0.52	0.78
	FCFP6	0.29	0.66	0.56	0.50	0.82	0.55	0.50	0.76	0.27	0.66	0.54	0.50	0.81	0.52	0.50	0.80
	KR	0.32	0.67	0.56	0.52	0.83	0.52	0.52	0.76	0.28	0.66	0.54	0.50	0.81	0.50	0.50	0.78
	LSTAR	0.27	0.65	0.55	0.48	0.81	0.53	0.48	0.74	0.29	0.66	0.55	0.51	0.82	0.53	0.51	0.78
	MACCS	0.32	0.68	0.56	0.53	0.83	0.52	0.53	0.77	0.34	0.69	0.57	0.55	0.83	0.53	0.55	0.81
	PUBCHEM	0.34	0.69	0.58	0.54	0.83	0.54	0.54	0.78	0.32	0.68	0.56	0.54	0.83	0.52	0.54	0.81
	2PPHAR	0.24	0.60	0.67	0.46	0.73	0.58	0.46	0.68	0.24	0.59	0.67	0.45	0.73	0.58	0.45	0.67
	3PPHAR	0.32	0.64	0.67	0.51	0.76	0.59	0.51	0.74	0.32	0.64	0.68	0.52	0.77	0.59	0.52	0.73
	AP2D	0.63	0.80	0.81	0.72	0.87	0.77	0.72	0.89	0.65	0.81	0.82	0.73	0.88	0.77	0.73	0.89
	ASP	0.68	0.82	0.84	0.75	0.89	0.81	0.75	0.91	0.71	0.84	0.85	0.78	0.90	0.83	0.78	0.91
	AT2D	0.68	0.82	0.84	0.75	0.88	0.82	0.75	0.92	0.73	0.84	0.86	0.78	0.90	0.85	0.78	0.93
	DFS	0.68	0.81	0.84	0.75	0.88	0.81	0.75	0.91	0.72	0.84	0.85	0.78	0.90	0.84	0.78	0.92
	ECFP0	0.54	0.75	0.76	0.66	0.84	0.70	0.66	0.85	0.57	0.77	0.77	0.69	0.86	0.72	0.69	0.85
	ECFP2	0.63	0.79	0.81	0.72	0.87	0.79	0.72	0.90	0.66	0.81	0.82	0.73	0.88	0.79	0.73	0.90
	ECFP4	0.62	0.78	0.81	0.71	0.86	0.79	0.71	0.89	0.64	0.80	0.82	0.72	0.87	0.80	0.72	0.89
Human Plasma Protein Binding	ECFP6	0.59	0.77	0.80	0.69	0.85	0.77	0.69	0.88	0.63	0.79	0.82	0.71	0.87	0.79	0.71	0.88
	ESTATE	0.51	0.74	0.74	0.65	0.84	0.68	0.65	0.84	0.53	0.75	0.76	0.66	0.84	0.69	0.66	0.84
	FCFP0	0.24	0.60	0.66	0.45	0.74	0.54	0.45	0.73	0.25	0.60	0.66	0.46	0.74	0.54	0.46	0.71
	FCFP2	0.62	0.79	0.80	0.71	0.87	0.76	0.71	0.88	0.62	0.80	0.81	0.72	0.87	0.76	0.72	0.88
	FCFP4	0.62	0.78	0.81	0.71	0.86	0.77	0.71	0.89	0.64	0.80	0.82	0.73	0.87	0.79	0.73	0.89
	FCFP6	0.60	0.77	0.80	0.69	0.86	0.76	0.69	0.88	0.66	0.80	0.83	0.73	0.88	0.80	0.73	0.89
	KR	0.64	0.80	0.81	0.72	0.87	0.78	0.72	0.90	0.67	0.82	0.83	0.75	0.88	0.80	0.75	0.90
	LSTAR	0.66	0.81	0.83	0.73	0.88	0.82	0.73	0.90	0.71	0.83	0.85	0.77	0.89	0.85	0.77	0.92
	MACCS	0.65	0.81	0.82	0.73	0.88	0.78	0.73	0.90	0.68	0.82	0.84	0.76	0.89	0.80	0.76	0.91
	PUBCHEM	0.68	0.82	0.83	0.76	0.89	0.79	0.76	0.92	0.71	0.84	0.85	0.78	0.90	0.81	0.78	0.92
	RAD2D	0.68	0.82	0.84	0.75	0.88	0.82	0.75	0.91	0.71	0.83	0.85	0.77	0.89	0.84	0.77	0.92
	2PPHAR	0.07	0.53	0.48	0.37	0.69	0.56	0.37	0.56	0.08	0.53	0.48	0.37	0.69	0.53	0.37	0.57
	3PPHAR	0.31	0.63	0.60	0.50	0.77	0.54	0.50	0.73	0.28	0.62	0.59	0.48	0.76	0.55	0.48	0.72
	AP2D	0.44	0.71	0.67	0.61	0.81	0.64	0.61	0.83	0.46	0.72	0.68	0.62	0.82	0.64	0.62	0.84
	ASP	0.51	0.75	0.71	0.66	0.84	0.69	0.66	0.86	0.54	0.76	0.73	0.67	0.84	0.72	0.67	0.88
	AT2D	0.52	0.75	0.72	0.65	0.84	0.70	0.65	0.86	0.50	0.74	0.71	0.65	0.83	0.69	0.65	0.87
	DFS	0.51	0.74	0.71	0.66	0.83	0.69	0.66	0.85	0.51	0.75	0.71	0.66	0.84	0.68	0.66	0.85
	ECFP0	0.42	0.70	0.66	0.59	0.81	0.63	0.59	0.81	0.46	0.72	0.68	0.62	0.82	0.66	0.62	0.83
	ECFP2	0.52	0.75	0.72	0.65	0.84	0.70	0.65	0.87	0.53	0.75	0.72	0.66	0.84	0.69	0.66	0.87
	ECFP4	0.51	0.74	0.71	0.65	0.83	0.69	0.65	0.86	0.53	0.75	0.72	0.66	0.84	0.71	0.66	0.87
Elimination half-life Human	ECFP6	0.51	0.74	0.71	0.65	0.84	0.71	0.65	0.86	0.49	0.73	0.70	0.64	0.83	0.67	0.64	0.84
	ESTATE	0.41	0.70	0.65	0.59	0.80	0.64	0.59	0.82	0.41	0.70	0.65	0.59	0.80	0.65	0.59	0.83
	FCFP0	0.18	0.58	0.53	0.44	0.73	0.49	0.44	0.68	0.18	0.58	0.53	0.44	0.73	0.52	0.44	0.68
	FCFP2	0.49	0.74	0.70	0.64	0.83	0.67	0.64	0.85	0.52	0.75	0.71	0.66	0.84	0.68	0.66	0.85
	FCFP4	0.50	0.74	0.71	0.64	0.83	0.68	0.64	0.86	0.53	0.76	0.72	0.68	0.84	0.71	0.68	0.88
	FCFP6	0.49	0.73	0.70	0.64	0.83	0.68	0.64	0.85	0.51	0.75	0.71	0.66	0.83	0.70	0.66	0.87
	KR	0.52	0.75	0.72	0.66	0.84	0.70	0.66	0.86	0.50	0.74	0.70	0.64	0.83	0.68	0.64	0.86
	LSTAR	0.47	0.72	0.69	0.62	0.82	0.68	0.62	0.85	0.50	0.73	0.71	0.63	0.83	0.68	0.63	0.85
	MACCS	0.49	0.73	0.70	0.64	0.83	0.68	0.64	0.86	0.53	0.76	0.72	0.68	0.84	0.71	0.68	0.87
	PUBCHEM	0.51	0.75	0.71	0.66	0.83	0.69	0.66	0.86	0.50	0.74	0.70	0.65	0.83	0.67	0.65	0.86
	RAD2D	0.52	0.75	0.72	0.65	0.84	0.70	0.65	0.86	0.50	0.74	0.70	0.65	0.83	0.69	0.65	0.86
	2PPHAR	0.10	0.55	0.61	0.40	0.70	0.56	0.40	0.60	0.17	0.57	0.64	0.42	0.72	0.65	0.42	0.61
	3PPHAR	0.34	0.65	0.67	0.52	0.78	0.59	0.52	0.75	0.33	0.64	0.66	0.51	0.78	0.54	0.51	0.73
	AP2D	0.45	0.70	0.72	0.60	0.81	0.69	0.60	0.84	0.45	0.72	0.73	0.62	0.81	0.67	0.62	0.85
	ASP	0.49	0.72	0.74	0.62	0.82	0.71	0.62	0.86	0.46	0.70	0.73	0.59	0.81	0.67	0.59	0.83
	AT2D	0.46	0.71	0.73	0.61	0.81	0.69	0.61	0.86	0.47	0.71	0.73	0.60	0.82	0.66	0.60	0.82
	DFS	0.49	0.72	0.74	0.62	0.83	0.69	0.62	0.85	0.48	0.73	0.73	0.63	0.83	0.68	0.63	0.86
	ECFP0	0.44	0.70	0.71	0.60	0.81	0.64	0.60	0.83	0.43	0.71	0.71	0.61	0.81	0.64	0.61	0.82
	ECFP2	0.53	0.74	0.76	0.64	0.84	0.73	0.64	0.86	0.49	0.72	0.74	0.62	0.82	0.69	0.62	0.84
	ECFP4	0.52	0.73	0.76	0.62	0.84	0.69	0.62	0.86	0.48	0.74	0.74	0.66	0.82	0.74	0.66	0.85
Elimination half-life Mouse	ECFP6	0.50	0.72	0.75	0.60	0.83	0.68	0.60	0.85	0.52	0.74	0.76	0.64	0.84	0.74	0.64	0.86
	ESTATE	0.45	0.71	0.72	0.60	0.81	0.66	0.60	0.83	0.48	0.71	0.74	0.60	0.82	0.71	0.60	0.84
	FCFP0	0.18	0.57	0.63	0.42	0.72	0.50	0.42	0.64	0.15	0.55	0.63	0.40	0.71	0.52	0.40	0.65
	FCFP2	0.47	0.71	0.73	0.60	0.82	0.67	0.60	0.86	0.42	0.68	0.71	0.57	0.80	0.62	0.57	0.84
	FCFP4	0.48	0.71	0.74	0.59	0.82	0.67	0.59	0.85	0.52	0.73	0.76	0.63	0.84	0.68	0.63	0.88
	FCFP6	0.50	0.72	0.75	0.62	0.83	0.72	0.62	0.86	0.48	0.71	0.74	0.59	0.82	0.67	0.59	0.86
	KR	0.51	0.73	0.75	0.63	0.83	0.71	0.63	0.85	0.48	0.72	0.73	0.62	0.82	0.66	0.62	0.85
	LSTAR	0.45	0.70	0.72	0.59	0.81	0.68	0.59	0.82	0.47	0.72	0.73	0.62	0.82	0.68	0.62	0.81
	MACCS	0.46	0.71	0.73	0.61	0.82	0.66	0.61	0.85	0.47	0.70	0.73	0.58	0.83	0.65	0.58	0.83
	PUBCHEM	0.50	0.72	0.74	0.61	0.83	0.68	0.61	0.86	0.46	0.71	0.72	0.60	0.82	0.65	0.60	0.85
	RAD2D	0.48	0.71	0.74	0.60	0.82	0.70	0.60	0.86	0.47	0.72	0.73	0.63	0.82	0.67	0.63	0.87
Elimination half-life Rat	2PPHAR	0.13	0.55	0.64	0.40	0.70	0.56	0.40	0.59	0.15	0.56	0.65	0.40	0.71	0.56	0.40	0.59
	3PPHAR	0.31	0.62	0.69	0.49	0.76	0.62	0.49	0.75	0.32	0.64	0.70	0.51	0.77	0.68	0.51	0.74
	AP2D	0.42	0.68	0.72	0.56	0.81	0.61	0.56	0.82	0.48	0.71	0.74	0.59	0.83	0.68	0.59	0.83

Continued on next page

Table S1 – *Continued from previous page*

Endpoint	FP	Calibration										Validation					
		κ	BACC	ACC	Se	Sp	AUC	κ	BACC	ACC	Se	Sp	AUC				
	ASP	0.50	0.72	0.75	0.60	0.84	0.68	0.60	0.86	0.54	0.74	0.77	0.63	0.85	0.67	0.63	0.86
	AT2D	0.49	0.72	0.75	0.60	0.83	0.67	0.60	0.87	0.43	0.69	0.72	0.57	0.81	0.62	0.57	0.81
	DFS	0.49	0.71	0.75	0.59	0.83	0.66	0.59	0.86	0.52	0.73	0.77	0.61	0.84	0.67	0.61	0.84
	ECFP0	0.44	0.70	0.72	0.58	0.82	0.63	0.58	0.82	0.48	0.72	0.74	0.62	0.83	0.66	0.62	0.83
	ECFP2	0.50	0.73	0.76	0.62	0.83	0.68	0.62	0.86	0.54	0.73	0.77	0.60	0.85	0.71	0.60	0.84
	ECFP4	0.49	0.72	0.75	0.60	0.83	0.69	0.60	0.86	0.53	0.74	0.77	0.63	0.85	0.67	0.63	0.85
	ECFP6	0.49	0.71	0.75	0.59	0.83	0.68	0.59	0.87	0.53	0.73	0.77	0.61	0.84	0.68	0.61	0.84
	ESTATE	0.45	0.70	0.73	0.59	0.82	0.64	0.59	0.84	0.50	0.72	0.75	0.61	0.83	0.65	0.61	0.82
	FCFP0	0.14	0.55	0.64	0.40	0.71	0.52	0.40	0.65	0.10	0.54	0.62	0.38	0.70	0.47	0.38	0.62
	FCFP2	0.52	0.73	0.76	0.62	0.84	0.67	0.62	0.87	0.57	0.75	0.78	0.64	0.86	0.69	0.64	0.86
	FCFP4	0.52	0.73	0.76	0.61	0.84	0.67	0.61	0.87	0.52	0.73	0.77	0.61	0.84	0.69	0.61	0.83
	FCFP6	0.50	0.72	0.75	0.60	0.84	0.68	0.60	0.85	0.52	0.73	0.77	0.62	0.84	0.67	0.62	0.83
	KR	0.53	0.74	0.77	0.62	0.85	0.69	0.62	0.86	0.53	0.74	0.77	0.63	0.85	0.67	0.63	0.83
	LSTAR	0.48	0.70	0.74	0.57	0.83	0.63	0.57	0.84	0.49	0.71	0.75	0.59	0.83	0.65	0.59	0.83
	MACCS	0.50	0.73	0.75	0.61	0.84	0.67	0.61	0.85	0.52	0.73	0.76	0.61	0.85	0.67	0.61	0.84
	PUBCHEM	0.50	0.72	0.75	0.61	0.83	0.68	0.61	0.87	0.54	0.74	0.77	0.63	0.85	0.68	0.63	0.85
	RAD2D	0.50	0.71	0.76	0.60	0.83	0.69	0.60	0.86	0.46	0.69	0.74	0.56	0.82	0.66	0.56	0.83

Table S2: Performance of classification models in training and validation sets.

**Endpoint**	**FP**	**Calibration**			**Validation**		
		R^2	RMSE	MAE	R^2	RMSE	MAE
	2PPHAR	0.07	0.86	0.66	0.06	0.94	0.70
	3PPHAR	0.13	0.84	0.64	0.12	0.86	0.65
	AP2D	0.33	0.74	0.52	0.31	0.76	0.51
	ASP	0.40	0.70	0.48	0.35	0.74	0.48
	AT2D	0.38	0.71	0.50	0.36	0.75	0.50
	DFS	0.40	0.70	0.48	0.36	0.72	0.48
	ECFP0	0.33	0.74	0.52	0.34	0.75	0.52
	ECFP2	0.41	0.70	0.47	0.45	0.66	0.45
	ECFP4	0.39	0.71	0.48	0.47	0.68	0.46
CACO2 Permeability	ECFP6	0.39	0.71	0.49	0.44	0.70	0.48
	ESTATE	0.30	0.75	0.53	0.39	0.72	0.51
	FCFP0	0.08	0.87	0.66	0.08	0.88	0.66
	FCFP2	0.40	0.70	0.48	0.47	0.67	0.46
	FCFP4	0.44	0.68	0.46	0.42	0.69	0.46
	FCFP6	0.39	0.71	0.48	0.46	0.64	0.46
	KR	0.41	0.69	0.47	0.37	0.74	0.49
	LSTAR	0.36	0.73	0.50	0.37	0.72	0.49
	MACCS	0.40	0.70	0.48	0.42	0.70	0.48
	PUBCHEM	0.41	0.70	0.47	0.45	0.67	0.45
	RAD2D	0.40	0.69	0.47	0.40	0.73	0.48
	2PPHAR	0.27	2.03	1.56	0.27	2.02	1.56
	3PPHAR	0.27	2.03	1.56	0.25	2.05	1.57
	AP2D	0.64	1.41	1.03	0.66	1.39	1.02
	ASP	0.67	1.37	0.98	0.68	1.34	0.93
	AT2D	0.66	1.40	1.01	0.67	1.35	0.98
	DFS	0.69	1.34	0.96	0.71	1.29	0.92
	ECFP0	0.52	1.65	1.24	0.53	1.62	1.22
	ECFP2	0.66	1.37	0.99	0.67	1.36	0.97
	ECFP4	0.67	1.38	1.00	0.68	1.33	0.96
Aqueous solubility (logS)	ECFP6	0.66	1.40	1.02	0.68	1.35	0.99
	ESTATE	0.49	1.70	1.27	0.50	1.67	1.26
	FCFP0	0.34	1.93	1.50	0.33	1.94	1.52
	FCFP2	0.62	1.47	1.08	0.64	1.43	1.05
	FCFP4	0.68	1.35	0.97	0.69	1.31	0.93
	FCFP6	0.68	1.35	0.97	0.69	1.31	0.93
	KR	0.67	1.36	0.99	0.69	1.33	0.96
	LSTAR	0.66	1.39	1.00	0.69	1.35	0.97
	MACCS	0.72	1.25	0.90	0.74	1.20	0.85
	PUBCHEM	0.77	1.15	0.81	0.78	1.12	0.78
	RAD2D	0.66	1.40	1.00	0.66	1.37	0.97
	2PPHAR	0.25	0.99	0.79	0.15	1.11	0.90
	3PPHAR	0.29	0.96	0.76	0.12	1.12	0.92
	AP2D	0.25	0.99	0.79	0.26	1.01	0.80
	ASP	0.40	0.90	0.71	0.35	0.97	0.74
	AT2D	0.42	0.88	0.70	0.33	1.00	0.78
	DFS	0.39	0.90	0.70	0.37	0.98	0.76
	ECFP0	0.30	0.95	0.78	0.28	1.03	0.82
	ECFP2	0.43	0.87	0.68	0.41	0.92	0.72
	ECFP4	0.45	0.86	0.68	0.35	0.98	0.76
Intrinsic Clearance (CL_{int})	ECFP6	0.40	0.90	0.71	0.42	0.97	0.75
	ESTATE	0.28	0.97	0.79	0.22	1.05	0.81
	FCFP0	0.14	1.06	0.86	0.16	1.11	0.89
	FCFP2	0.31	0.95	0.76	0.30	1.00	0.78
	FCFP4	0.36	0.91	0.72	0.31	0.99	0.77
	FCFP6	0.35	0.93	0.73	0.30	1.00	0.77
	KR	0.39	0.90	0.71	0.32	0.98	0.76
	LSTAR	0.39	0.91	0.72	0.32	1.00	0.78
	MACCS	0.38	0.90	0.72	0.27	1.03	0.78
	PUBCHEM	0.41	0.87	0.70	0.28	1.01	0.80
	RAD2D	0.49	0.83	0.65	0.34	0.98	0.77
	2PPHAR	0.12	1.08	0.86	0.17	1.02	0.82
	3PPHAR	0.22	0.99	0.78	0.34	1.01	0.79
	AP2D	0.59	0.73	0.58	0.63	0.70	0.54
	ASP	0.58	0.73	0.59	0.60	0.77	0.61
	AT2D	0.53	0.79	0.64	0.66	0.65	0.51
	DFS	0.58	0.74	0.61	0.61	0.68	0.55
	ECFP0	0.53	0.79	0.64	0.62	0.71	0.55

Continued on next page

Skin Penetration

Table S2 – *Continued from previous page*

**Endpoint**	**FP**	**Calibration**			**Validation**		
		R^2	RMSE	MAE	R^2	RMSE	MAE
	ECFP2	0.59	0.73	0.60	0.64	0.71	0.57
	ECFP4	0.62	0.71	0.58	0.62	0.70	0.55
	ECFP6	0.55	0.76	0.62	0.68	0.71	0.57
	ESTATE	0.51	0.79	0.64	0.50	0.81	0.64
	FCFP0	0.45	0.84	0.67	0.47	0.85	0.67
	FCFP2	0.62	0.71	0.56	0.70	0.65	0.50
	FCFP4	0.67	0.68	0.54	0.73	0.61	0.48
	FCFP6	0.66	0.67	0.54	0.68	0.69	0.55
	KR	0.64	0.68	0.55	0.67	0.65	0.52
	LSTAR	0.55	0.78	0.64	0.57	0.76	0.62
	MACCS	0.73	0.59	0.46	0.75	0.59	0.45
	PUBCHEM	0.71	0.62	0.49	0.74	0.57	0.42
	RAD2D	0.63	0.71	0.57	0.64	0.67	0.52
Human Serum Albumin	2PPHAR	0.37	0.50	0.38	0.35	0.49	0.37
	3PPHAR	0.49	0.45	0.33	0.51	0.43	0.31
	AP2D	0.71	0.33	0.23	0.70	0.39	0.25
	ASP	0.71	0.35	0.23	0.79	0.30	0.20
	AT2D	0.73	0.35	0.23	0.85	0.22	0.16
	DFS	0.69	0.37	0.24	0.74	0.29	0.21
	ECFP0	0.61	0.40	0.28	0.60	0.41	0.26
	ECFP2	0.67	0.37	0.26	0.62	0.38	0.24
	ECFP4	0.66	0.37	0.27	0.64	0.43	0.27
	ECFP6	0.60	0.41	0.29	0.71	0.36	0.26
	ESTATE	0.62	0.39	0.27	0.71	0.34	0.23
	FCFP0	0.31	0.52	0.37	0.39	0.52	0.37
	FCFP2	0.62	0.39	0.27	0.70	0.32	0.23
	FCFP4	0.67	0.37	0.26	0.72	0.36	0.24
	FCFP6	0.65	0.39	0.28	0.74	0.30	0.22
	KR	0.71	0.34	0.22	0.78	0.31	0.21
	LSTAR	0.67	0.38	0.25	0.72	0.35	0.23
	MACCS	0.74	0.33	0.22	0.65	0.36	0.24
	PUBCHEM	0.73	0.34	0.23	0.79	0.27	0.19
	RAD2D	0.73	0.34	0.22	0.84	0.25	0.17
Human Placenta Barrier (clearance index)	2PPHAR	0.14	0.30	0.25	0.10	0.28	0.25
	3PPHAR	0.15	0.30	0.24	0.11	0.27	0.23
	AP2D	0.29	0.27	0.22	0.30	0.25	0.22
	ASP	0.30	0.28	0.23	0.36	0.24	0.20
	AT2D	0.21	0.29	0.24	0.40	0.23	0.19
	DFS	0.23	0.28	0.24	0.43	0.24	0.20
	ECFP0	0.28	0.27	0.22	0.32	0.27	0.21
	ECFP2	0.46	0.25	0.20	0.33	0.27	0.20
	ECFP4	0.38	0.25	0.21	0.38	0.28	0.22
	ECFP6	0.33	0.27	0.22	0.26	0.27	0.23
	ESTATE	0.29	0.27	0.22	0.31	0.24	0.21
	FCFP0	0.30	0.26	0.21	0.18	0.33	0.25
	FCFP2	0.29	0.26	0.20	0.45	0.24	0.20
	FCFP4	0.42	0.24	0.19	0.16	0.32	0.23
	FCFP6	0.38	0.24	0.19	0.22	0.32	0.23
	KR	0.41	0.24	0.20	0.24	0.32	0.22
	LSTAR	0.31	0.27	0.22	0.20	0.27	0.23
	MACCS	0.40	0.25	0.20	0.41	0.23	0.19
	PUBCHEM	0.32	0.26	0.20	0.36	0.26	0.22
	RAD2D	0.21	0.28	0.23	0.51	0.23	0.19
Cancer potency in mouse (TD_{50})	2PPHAR	0.03	1.14	0.90	0.02	1.21	0.93
	3PPHAR	0.10	1.13	0.88	0.08	1.11	0.88
	AP2D	0.26	1.02	0.77	0.23	1.00	0.79
	ASP	0.27	1.03	0.77	0.25	0.98	0.75
	AT2D	0.33	0.98	0.75	0.27	0.96	0.72
	DFS	0.31	0.98	0.74	0.29	0.98	0.73
	ECFP0	0.16	1.07	0.80	0.22	1.04	0.82
	ECFP2	0.22	1.05	0.80	0.25	0.98	0.77
	ECFP4	0.22	1.06	0.81	0.27	0.96	0.71
	ECFP6	0.23	1.00	0.77	0.17	1.21	0.84
	ESTATE	0.15	1.09	0.84	0.15	1.07	0.79
	FCFP0	0.04	1.15	0.91	0.02	1.18	0.92
	FCFP2	0.24	1.03	0.78	0.25	0.98	0.74
	FCFP4	0.27	1.03	0.78	0.25	0.95	0.74
	FCFP6	0.31	0.99	0.76	0.23	1.04	0.77
	KR	0.27	1.01	0.77	0.18	1.08	0.84

Continued on next page

Table S2 – *Continued from previous page*

**Endpoint**	**FP**	**Calibration**			**Validation**		
		R^2	RMSE	MAE	R^2	RMSE	MAE
	LSTAR	0.22	1.07	0.80	0.14	1.02	0.81
	MACCS	0.28	0.99	0.74	0.24	1.01	0.80
	PUBCHEM	0.31	0.97	0.74	0.24	1.06	0.77
	RAD2D	0.27	1.00	0.76	0.23	1.07	0.79
Cancer potency in rat (TD_{50})	2PPHAR	0.05	1.36	1.11	0.01	1.39	1.13
	3PPHAR	0.10	1.33	1.07	0.05	1.39	1.14
	AP2D	0.32	1.15	0.89	0.35	1.12	0.87
	ASP	0.37	1.12	0.85	0.44	1.00	0.78
	AT2D	0.41	1.08	0.83	0.35	1.14	0.87
	DFS	0.36	1.12	0.86	0.41	1.05	0.81
	ECFP0	0.29	1.19	0.92	0.32	1.13	0.85
	ECFP2	0.38	1.11	0.86	0.34	1.13	0.88
	ECFP4	0.34	1.14	0.88	0.44	1.05	0.82
	ECFP6	0.34	1.16	0.90	0.45	1.00	0.78
	ESTATE	0.33	1.16	0.90	0.27	1.14	0.89
	FCFP0	0.08	1.35	1.09	0.09	1.34	1.11
	FCFP2	0.35	1.14	0.88	0.32	1.09	0.86
	FCFP4	0.38	1.11	0.86	0.38	1.09	0.81
	FCFP6	0.36	1.13	0.86	0.45	1.01	0.81
	KR	0.34	1.14	0.88	0.37	1.06	0.82
	LSTAR	0.32	1.16	0.89	0.34	1.12	0.86
	MACCS	0.39	1.08	0.84	0.33	1.17	0.88
	PUBCHEM	0.40	1.09	0.85	0.35	1.09	0.84
	RAD2D	0.40	1.08	0.82	0.40	1.15	0.86
Steady state volume distribution (VD_{ss})	2PPHAR	0.16	0.61	0.47	0.11	0.65	0.50
	3PPHAR	0.17	0.61	0.48	0.17	0.63	0.49
	AP2D	0.48	0.48	0.33	0.40	0.54	0.36
	ASP	0.57	0.44	0.28	0.45	0.51	0.32
	AT2D	0.57	0.44	0.28	0.46	0.51	0.32
	AVALON	0.50	0.47	0.33	0.43	0.52	0.34
	DFS	0.55	0.45	0.29	0.45	0.51	0.32
	ECFP0	0.48	0.48	0.35	0.44	0.52	0.37
	ECFP2	0.56	0.45	0.32	0.48	0.50	0.33
	ECFP4	0.47	0.49	0.36	0.42	0.52	0.36
	ECFP6	0.46	0.50	0.37	0.37	0.54	0.38
	ESTATE	0.43	0.51	0.37	0.42	0.53	0.37
	FCFP0	0.34	0.54	0.41	0.31	0.57	0.42
	FCFP2	0.54	0.45	0.32	0.47	0.50	0.33
	FCFP4	0.56	0.44	0.32	0.47	0.50	0.34
	FCFP6	0.47	0.49	0.36	0.44	0.52	0.36
	KR	0.56	0.44	0.30	0.47	0.50	0.33
	LSTAR	0.57	0.44	0.29	0.43	0.52	0.33
	MACCS	0.54	0.45	0.30	0.47	0.50	0.32
	MAP4	0.48	0.49	0.35	0.39	0.54	0.38
	PUBCHEM	0.54	0.46	0.30	0.46	0.51	0.33
	RAD2D	0.57	0.44	0.28	0.46	0.51	0.31
Distribution coefficient (log D)	2PPHAR	0.20	1.32	1.04	0.22	1.30	1.03
	3PPHAR	0.30	1.24	0.96	0.30	1.22	0.96
	AP2D	0.66	0.87	0.62	0.68	0.84	0.59
	ASP	0.74	0.77	0.55	0.76	0.74	0.52
	AT2D	0.73	0.79	0.56	0.77	0.75	0.51
	DFS	0.73	0.79	0.56	0.75	0.75	0.52
	ECFP0	0.56	0.98	0.75	0.60	0.94	0.72
	ECFP2	0.72	0.80	0.59	0.74	0.77	0.57
	ECFP4	0.70	0.83	0.62	0.73	0.79	0.58
	ECFP6	0.67	0.87	0.65	0.70	0.84	0.63
	ESTATE	0.53	1.01	0.77	0.57	0.98	0.75
	FCFP0	0.37	1.16	0.91	0.39	1.16	0.92
	FCFP2	0.70	0.82	0.61	0.72	0.79	0.58
	FCFP4	0.70	0.81	0.59	0.72	0.79	0.57
	FCFP6	0.69	0.83	0.61	0.70	0.82	0.59
	KR	0.71	0.80	0.59	0.74	0.76	0.55
	LSTAR	0.71	0.81	0.58	0.75	0.77	0.54
	MACCS	0.73	0.78	0.58	0.74	0.75	0.55
	PUBCHEM	0.76	0.73	0.53	0.77	0.71	0.50
	RAD2D	0.75	0.76	0.53	0.78	0.70	0.48
Fraction Unbound in Human Plasma	2PPHAR	0.20	0.64	0.52	0.15	0.65	0.53
	3PPHAR	0.22	0.63	0.51	0.18	0.65	0.51
	AP2D	0.44	0.54	0.42	0.44	0.53	0.41

Continued on next page

Table S2 – *Continued from previous page*

**Endpoint**	**FP**	**Calibration**			**Validation**		
		R^2	RMSE	MAE	R^2	RMSE	MAE
	ASP	0.57	0.47	0.37	0.60	0.45	0.34
	AT2D	0.57	0.48	0.37	0.56	0.47	0.36
	DFS	0.56	0.48	0.37	0.58	0.46	0.35
	ECFP0	0.41	0.55	0.44	0.40	0.55	0.43
	ECFP2	0.57	0.47	0.37	0.60	0.45	0.34
	ECFP4	0.51	0.51	0.40	0.52	0.49	0.38
	ECFP6	0.51	0.51	0.40	0.53	0.49	0.38
	ESTATE	0.40	0.56	0.44	0.43	0.54	0.42
	FCFP0	0.22	0.64	0.51	0.21	0.63	0.51
	FCFP2	0.54	0.49	0.38	0.54	0.48	0.37
	FCFP4	0.59	0.46	0.36	0.58	0.46	0.35
	FCFP6	0.53	0.50	0.40	0.53	0.49	0.38
	KR	0.58	0.47	0.36	0.60	0.45	0.35
	LSTAR	0.55	0.49	0.39	0.54	0.48	0.38
	MACCS	0.56	0.48	0.37	0.58	0.46	0.35
	MAP4	0.53	0.50	0.40	0.52	0.50	0.39
	PUBCHEM	0.60	0.46	0.35	0.63	0.43	0.33
	RAD2D	0.58	0.47	0.36	0.61	0.44	0.34
Fraction Unbound in the Brain	2PPHAR	0.26	0.69	0.55	0.27	0.71	0.56
	3PPHAR	0.24	0.69	0.56	0.31	0.68	0.52
	AP2D	0.31	0.67	0.52	0.27	0.66	0.51
	ASP	0.38	0.63	0.50	0.38	0.65	0.52
	AT2D	0.32	0.67	0.53	0.38	0.63	0.52
	DFS	0.35	0.65	0.52	0.41	0.63	0.49
	ECFP0	0.24	0.69	0.55	0.26	0.73	0.57
	ECFP2	0.36	0.63	0.49	0.43	0.64	0.48
	ECFP4	0.39	0.64	0.50	0.41	0.63	0.49
	ECFP6	0.36	0.64	0.50	0.39	0.66	0.50
	ESTATE	0.22	0.71	0.56	0.24	0.70	0.55
	FCFP0	0.15	0.74	0.60	0.19	0.73	0.60
	FCFP2	0.31	0.67	0.53	0.28	0.65	0.51
	FCFP4	0.39	0.63	0.50	0.35	0.64	0.50
	FCFP6	0.40	0.63	0.49	0.30	0.65	0.51
	KR	0.34	0.65	0.52	0.44	0.60	0.47
	LSTAR	0.34	0.67	0.53	0.28	0.68	0.54
	MACCS	0.38	0.63	0.50	0.44	0.61	0.47
	PUBCHEM	0.48	0.58	0.46	0.54	0.56	0.44
	RAD2D	0.39	0.63	0.50	0.42	0.62	0.48
Human Liver Microsomal Clearance	2PPHAR	0.07	1.49	1.19	0.06	1.52	1.21
	3PPHAR	0.16	1.42	1.11	0.20	1.41	1.11
	AP2D	0.33	1.26	0.96	0.40	1.22	0.94
	ASP	0.51	1.08	0.81	0.57	1.04	0.79
	AT2D	0.48	1.13	0.86	0.55	1.08	0.84
	DFS	0.49	1.11	0.84	0.56	1.06	0.81
	ECFP0	0.37	1.23	0.93	0.39	1.23	0.93
	ECFP2	0.50	1.09	0.82	0.58	1.02	0.78
	ECFP4	0.50	1.09	0.83	0.56	1.05	0.80
	ECFP6	0.49	1.12	0.85	0.56	1.07	0.82
	ESTATE	0.34	1.25	0.95	0.36	1.26	0.96
	FCFP0	0.09	1.48	1.17	0.09	1.50	1.19
	FCFP2	0.50	1.10	0.82	0.55	1.06	0.81
	FCFP4	0.51	1.08	0.81	0.56	1.05	0.80
	FCFP6	0.50	1.10	0.82	0.56	1.06	0.81
	KR	0.51	1.08	0.80	0.56	1.05	0.79
	LSTAR	0.46	1.15	0.88	0.53	1.11	0.85
	MACCS	0.47	1.12	0.84	0.53	1.08	0.82
	PUBCHEM	0.49	1.10	0.83	0.56	1.05	0.80
	RAD2D	0.50	1.10	0.83	0.56	1.06	0.81
Rat Liver Microsomal Clearance	2PPHAR	0.06	1.73	1.36	0.03	1.80	1.42
	3PPHAR	0.23	1.56	1.22	0.24	1.56	1.22
	AP2D	0.53	1.23	0.93	0.57	1.19	0.89
	ASP	0.63	1.10	0.84	0.62	1.09	0.84
	AT2D	0.61	1.14	0.88	0.64	1.09	0.84
	DFS	0.62	1.12	0.86	0.62	1.08	0.83
	ECFP0	0.44	1.34	1.01	0.49	1.24	0.94
	ECFP2	0.64	1.08	0.82	0.70	0.98	0.74
	ECFP4	0.63	1.10	0.84	0.66	1.08	0.80
	ECFP6	0.61	1.15	0.88	0.63	1.09	0.84
	ESTATE	0.43	1.35	1.03	0.45	1.32	0.99

Continued on next page

Table S2 – *Continued from previous page*

**Endpoint**	**FP**	**Calibration**			**Validation**		
		R^2	RMSE	MAE	R^2	RMSE	MAE
	FCFP0	0.12	1.68	1.32	0.10	1.69	1.34
	FCFP2	0.62	1.11	0.85	0.62	1.12	0.84
	FCFP4	0.64	1.07	0.81	0.66	1.05	0.79
	FCFP6	0.64	1.10	0.84	0.62	1.08	0.82
	KR	0.64	1.08	0.83	0.67	1.01	0.76
	LSTAR	0.57	1.19	0.91	0.61	1.16	0.89
	MACCS	0.60	1.14	0.87	0.61	1.13	0.86
	PUBCHEM	0.63	1.09	0.83	0.65	1.06	0.80
	RAD2D	0.62	1.11	0.85	0.66	1.05	0.81
Mouse Liver Microsomal Clearance	2PPHAR	0.02	1.73	1.39	0.03	1.73	1.39
	3PPHAR	0.20	1.56	1.22	0.20	1.57	1.23
	AP2D	0.49	1.25	0.95	0.41	1.34	1.01
	ASP	0.52	1.21	0.92	0.52	1.20	0.90
	AT2D	0.52	1.21	0.92	0.53	1.16	0.88
	DFS	0.50	1.24	0.95	0.53	1.16	0.89
	ECFP0	0.44	1.31	1.00	0.47	1.26	0.96
	ECFP2	0.51	1.21	0.91	0.48	1.27	0.93
	ECFP4	0.49	1.24	0.94	0.56	1.15	0.86
	ECFP6	0.48	1.24	0.94	0.53	1.24	0.95
	ESTATE	0.44	1.31	1.00	0.43	1.30	0.99
	FCFP0	0.04	1.73	1.38	0.03	1.70	1.36
	FCFP2	0.48	1.25	0.94	0.54	1.20	0.91
	FCFP4	0.51	1.22	0.93	0.50	1.26	0.95
	FCFP6	0.50	1.25	0.96	0.56	1.13	0.85
	KR	0.50	1.23	0.93	0.48	1.28	0.94
	LSTAR	0.49	1.26	0.98	0.48	1.23	0.94
	MACCS	0.47	1.25	0.96	0.52	1.26	0.92
	PUBCHEM	0.50	1.22	0.91	0.61	1.12	0.85
	RAD2D	0.52	1.21	0.91	0.53	1.19	0.89
Skin Permeability	2PPHAR	0.10	1.09	0.89	0.09	1.09	0.89
	3PPHAR	0.16	1.05	0.85	0.30	0.95	0.75
	AP2D	0.49	0.81	0.66	0.50	0.78	0.60
	ASP	0.53	0.78	0.63	0.56	0.77	0.61
	AT2D	0.49	0.82	0.67	0.50	0.79	0.63
	DFS	0.52	0.79	0.63	0.58	0.73	0.59
	ECFP0	0.47	0.83	0.66	0.56	0.76	0.62
	ECFP2	0.51	0.79	0.64	0.53	0.79	0.63
	ECFP4	0.50	0.80	0.65	0.61	0.73	0.59
	ECFP6	0.49	0.82	0.66	0.52	0.80	0.65
	ESTATE	0.47	0.82	0.66	0.45	0.84	0.66
	FCFP0	0.36	0.92	0.74	0.35	0.89	0.71
	FCFP2	0.52	0.78	0.62	0.57	0.77	0.62
	FCFP4	0.52	0.79	0.63	0.53	0.77	0.60
	FCFP6	0.55	0.77	0.62	0.50	0.81	0.64
	KR	0.55	0.76	0.61	0.51	0.80	0.63
	LSTAR	0.48	0.82	0.66	0.47	0.84	0.68
	MACCS	0.58	0.73	0.58	0.62	0.71	0.57
	PUBCHEM	0.60	0.73	0.57	0.50	0.77	0.63
	RAD2D	0.53	0.78	0.63	0.58	0.75	0.60
pK_a	2PPHAR	0.13	3.18	2.46	0.14	3.14	2.43
	3PPHAR	0.15	3.14	2.45	0.15	3.13	2.43
	AP2D	0.30	2.87	2.13	0.28	2.89	2.13
	ASP	0.40	2.64	1.88	0.42	2.60	1.85
	AT2D	0.30	2.85	2.06	0.28	2.92	2.10
	DFS	0.38	2.68	1.87	0.40	2.68	1.86
	ECFP0	0.59	2.19	1.52	0.60	2.17	1.49
	ECFP2	0.71	1.85	1.15	0.74	1.78	1.11
	ECFP4	0.68	1.97	1.25	0.72	1.81	1.16
	ECFP6	0.67	2.01	1.32	0.66	2.04	1.30
	ESTATE	0.48	2.46	1.74	0.50	2.40	1.69
	FCFP0	0.39	2.66	1.94	0.38	2.72	1.96
	FCFP2	0.63	2.08	1.36	0.65	2.02	1.32
	FCFP4	0.61	2.13	1.38	0.63	2.07	1.32
	FCFP6	0.59	2.20	1.43	0.61	2.11	1.37
	KR	0.58	2.20	1.42	0.63	2.10	1.34
	LSTAR	0.33	2.80	1.99	0.35	2.78	1.96
	MACCS	0.69	1.92	1.19	0.72	1.79	1.11
	PUBCHEM	0.58	2.20	1.41	0.60	2.15	1.37

Continued on next page

Table S2 – *Continued from previous page*

**Endpoint**	**FP**	**Calibration**			**Validation**		
		R^2	RMSE	MAE	R^2	RMSE	MAE
	RAD2D	0.34	2.76	2.00	0.35	2.74	1.97
MDCK cell line permeability	2PPHAR	0.29	0.84	0.69	0.29	0.82	0.68
	3PPHAR	0.36	0.79	0.62	0.46	0.73	0.57
	AP2D	0.59	0.63	0.46	0.63	0.61	0.42
	ASP	0.59	0.63	0.45	0.66	0.59	0.42
	AT2D	0.57	0.65	0.48	0.66	0.57	0.41
	DFS	0.59	0.62	0.45	0.67	0.60	0.42
	ECFP0	0.61	0.61	0.45	0.61	0.62	0.44
	ECFP2	0.63	0.61	0.43	0.59	0.62	0.45
	ECFP4	0.62	0.61	0.44	0.68	0.56	0.39
	ECFP6	0.60	0.62	0.45	0.68	0.56	0.39
	ESTATE	0.57	0.65	0.47	0.58	0.63	0.46
	FCFP0	0.35	0.80	0.63	0.41	0.74	0.61
	FCFP2	0.59	0.63	0.45	0.66	0.60	0.43
	FCFP4	0.59	0.63	0.46	0.66	0.56	0.39
	FCFP6	0.62	0.61	0.44	0.65	0.59	0.42
	KR	0.58	0.63	0.47	0.70	0.56	0.40
	LSTAR	0.60	0.62	0.45	0.61	0.61	0.43
	MACCS	0.61	0.61	0.43	0.62	0.62	0.43
	PUBCHEM	0.61	0.62	0.43	0.61	0.63	0.43
	RAD2D	0.61	0.62	0.44	0.58	0.63	0.44
Human Renal Clearance (CL_r)	2PPHAR	0.13	0.59	0.48	0.13	0.57	0.46
	3PPHAR	0.14	0.58	0.47	0.14	0.56	0.46
	AP2D	0.19	0.57	0.45	0.22	0.55	0.44
	ASP	0.20	0.56	0.46	0.19	0.57	0.47
	AT2D	0.23	0.55	0.45	0.17	0.56	0.45
	DFS	0.20	0.57	0.46	0.20	0.57	0.46
	ECFP0	0.14	0.57	0.45	0.19	0.60	0.47
	ECFP2	0.25	0.54	0.42	0.23	0.55	0.42
	ECFP4	0.22	0.55	0.43	0.21	0.57	0.43
	ECFP6	0.19	0.57	0.45	0.23	0.55	0.44
	ESTATE	0.15	0.57	0.45	0.14	0.58	0.46
	FCFP0	0.11	0.60	0.49	0.12	0.59	0.48
	FCFP2	0.23	0.54	0.42	0.24	0.55	0.43
	FCFP4	0.24	0.55	0.43	0.22	0.54	0.42
	FCFP6	0.21	0.56	0.44	0.19	0.56	0.44
	KR	0.22	0.56	0.43	0.20	0.53	0.42
	LSTAR	0.15	0.58	0.47	0.14	0.60	0.47
	MACCS	0.25	0.54	0.43	0.27	0.53	0.42
	PUBCHEM	0.23	0.55	0.43	0.25	0.55	0.43
	RAD2D	0.23	0.54	0.42	0.18	0.60	0.45
Hemolytic toxicity (log HD_{50})	2PPHAR	0.17	0.75	0.57	0.17	0.75	0.56
	3PPHAR	0.45	0.61	0.47	0.49	0.58	0.43
	AP2D	0.51	0.58	0.45	0.47	0.58	0.45
	ASP	0.68	0.47	0.35	0.68	0.44	0.34
	AT2D	0.67	0.48	0.36	0.68	0.46	0.36
	DFS	0.68	0.47	0.35	0.67	0.47	0.35
	ECFP0	0.54	0.56	0.42	0.57	0.57	0.42
	ECFP2	0.64	0.49	0.37	0.65	0.52	0.38
	ECFP4	0.65	0.48	0.36	0.68	0.48	0.35
	ECFP6	0.65	0.49	0.36	0.65	0.50	0.37
	ESTATE	0.48	0.60	0.45	0.56	0.54	0.42
	FCFP0	0.26	0.71	0.54	0.28	0.70	0.53
	FCFP2	0.61	0.51	0.39	0.66	0.48	0.37
	FCFP4	0.64	0.50	0.37	0.69	0.46	0.34
	FCFP6	0.67	0.47	0.36	0.63	0.50	0.37
	KR	0.65	0.48	0.37	0.65	0.50	0.37
	LSTAR	0.65	0.49	0.36	0.69	0.47	0.34
	MACCS	0.64	0.49	0.37	0.63	0.51	0.39
	PUBCHEM	0.66	0.48	0.37	0.57	0.53	0.38
	RAD2D	0.63	0.50	0.37	0.74	0.44	0.34

References

H. Baba, Y. Ueno, M. Hashida, and F. Yamashita. Quantitative prediction of ionization effect on human skin permeability. *Int. J. Pharm.*, 522(1-2):222–233, 2017. doi: 10.1016/j.ijpharm.2017.03.009.

J. P. Bercu, S. M. Morton, J. T. Deahl, V. K. Gombar, C. M. Callis, and R. B. van Lier. In silico approaches to predicting cancer potency for risk assessment of genotoxic impurities in drug substances. *Regul. Toxicol. Pharmacol.*, 57(2):300–306, 2010. doi: https://doi.org/10.1016/j.yrtph.2010.03.010.

J. V. B. Borba, R. C. Braga, V. M. Alves, E. N. Muratov, N. Kleinstreuer, A. Tropsha, and C. H. Andrade. Pred-skin: A web portal for accurate prediction of human skin sensitizers. *Chemical Research in Toxicology*, 34(2):258–267, 2020. doi: 10.1021/acs.chemrestox.0c00186.

F. Broccatelli, E. Carosati, A. Neri, M. Frosini, L. Goracci, T. I. Oprea, and G. Cruciani. A novel approach for predicting p-glycoprotein (ABCB1) inhibition using molecular interaction fields. *Journal of Medicinal Chemistry*, 54(6):1740–1751, Feb. 2011. doi: 10.1021/jm101421d. URL `https://doi.org/10.1021/jm101421d`.

C. Cai, P. Guo, Y. Zhou, J. Zhou, Q. Wang, F. Zhang, J. Fang, and F. Cheng. Deep learning-based prediction of drug-induced cardiotoxicity. *J Chem. Inf. Model.*, 59(3):1073–1084, 2019. doi: 10.1021/acs.jcim.8b00769.

J. Chen, H. Yang, L. Zhu, Z. Wu, W. Li, Y. Tang, and G. Liu. In silico prediction of human renal clearance of compounds using quantitative structure-pharmacokinetic relationship models. *Chemical Research in Toxicology*, 33(2):640–650, 2020. doi: 10.1021/acs.chemrestox.9b00447.

L. Chen, Y. Li, H. Yu, L. Zhang, and T. Hou. Computational models for predicting substrates or inhibitors of p-glycoprotein. *Drug Discovery Today*, 17(7-8):343–351, 2012. doi: 10.1016/j.drudis.2011.11.003.

K. Ciura, S. Ulenberg, H. Kapica, P. Kawczak, M. Belka, and T. Bączek. Drug affinity to human serum albumin prediction by retention of cetyltrimethylammonium bromide pseudostationary phase in micellar electrokinetic chromatography and chemically advanced template search descriptors. *J. Pharm. Biomed.*, 188:113423, 2020. doi: 10.1016/j.jpba.2020.113423.

X. Cui, J. Liu, J. Zhang, Q. Wu, and X. Li. In silico prediction of drug-induced rhabdomyolysis with machine-learning models and structural alerts. *J. Appl. Toxicol.*, 39(8):1224–1232, 2019. doi: 10.1002/jat.3808.

S. Dik, J. Ezendam, A. R. Cunningham, C. A. Carrasquer, H. van Loveren, and E. Rorije. Evaluation ofIn SilicoModels for the identification of respiratory sensitizers. *Toxicological Sciences*, 142(2):385–394, 2014. doi: 10.1093/toxsci/kfu188.

S. Dik, J. L. Pennings, H. van Loveren, and J. Ezendam. Development of an in vitro test to identify respiratory sensitizers in bronchial epithelial cells using gene expression profiling. *Toxicology in Vitro*, 30(1):274–280, 2015. doi: 10.1016/j.tiv.2015.10.010.

T. Esaki, R. Watanabe, H. Kawashima, R. Ohashi, Y. Natsume-Kitatani, C. Nagao, and K. Mizuguchi. Data curation can improve the prediction accuracy of metabolic intrinsic clearance. *Mol. Inf.*, 38(1-2):1800086, 2018. doi: 10.1002/minf.201800086.

T. Esaki, R. Ohashi, R. Watanabe, Y. Natsume-Kitatani, H. Kawashima, C. Nagao, and K. Mizuguchi. Computational model to predict the fraction of unbound drug in the brain. *J Chem. Inf. Model.*, 59(7):3251–3261, 2019. doi: 10.1021/acs.jcim.9b00180.

C. Esposito, S. Wang, U. E. W. Lange, F. Oellien, and S. Riniker. Combining machine learning and molecular dynamics to predict p-glycoprotein substrates. *Journal of Chemical Information and Modeling*, 60(10):4730–4749, Aug. 2020. doi: 10.1021/acs.jcim.0c00525. URL `https://doi.org/10.1021/acs.jcim.0c00525`.

G. Falcón-Cano, C. Molina, and M. Á. Cabrera-Pérez. ADME prediction with KNIME: Development and validation of a publicly available workflow for the prediction of human oral bioavailability. *Journal of Chemical Information and Modeling*, 60(6):2660–2667, 2020. doi: 10.1021/acs.jcim.0c00019. URL `https://doi.org/10.1021/acs.jcim.0c00019`.

L. Fu, L. Liu, Z.-J. Yang, P. Li, J.-J. Ding, Y.-H. Yun, A.-P. Lu, T.-J. Hou, and D.-S. Cao. Systematic modeling of log $d7.4$ based on ensemble mach. learn., group contribution, and matched molecular pair analysis. *J Chem. Inf. Model.*, 60(1):63–76, 2019. doi: 10.1021/acs.jcim.9b00718.

L. Fusani, M. Brown, H. Chen, E. Ahlberg, and T. Noeske. Predicting the risk of phospholipidosis with in silico models and an image-based in vitro screen. *Mol. Pharm.*, 14(12):4346–4352, 2017. doi: 10.1021/acs.molpharmaceut.7b00388.

D. Gadaleta, K. Vuković, C. Toma, G. J. Lavado, A. L. Karmaus, K. Mansouri, N. C. Kleinstreuer, E. Benfenati, and A. Roncaglioni. SAR and QSAR modeling of a large collection of LD50 rat acute oral toxicity data. *J Cheminf.*, 11(1), 2019. doi: 10.1186/s13321-019-0383-2.

C. Giaginis, A. Zira, S. Theocharis, and A. Tsantili-Kakoulidou. Application of quantitative structure activity relationships for modeling drug and chemical transport across the human placenta barrier: a multivariate data analysis approach. *J. Appl. Toxicol.*, 29(8):724–733, 2009. doi: 10.1002/jat.1466.

J. Hemmerich, F. Troger, B. Füzi, and G. F.Ecker. Using mach. learn. methods and structural alerts for prediction of mitochondrial toxicity. *Mol. Inf.*, 39(5):2000005, 2020. doi: 10.1002/minf.202000005.

Y.-W. Hsiao, U. Fagerholm, and U. Norinder. In SilicoCategorization ofin VivoIntrinsic clearance using mach. learn. *Mol. Pharm.*, 10(4):1318–1321, 2013. doi: 10.1021/mp300484r.

X. Hu and A. Yan. In silico models to discriminate compounds inducing and noninducing toxic myopathy. *Mol. Inf.*, 31(1):27–39, 2011. doi: 10.1002/minf.201100067.

S. Jain, U. Norinder, S. E. Escher, and B. Zdrazil. Combining in vivo data with in silico predictions for modeling hepatic steatosis by using stratified bagging and conformal prediction. *Chem. Res. Toxicol.*, 34(2), 2020. doi: 10.1021/acs.chemrestox.0c00511.

D. Jiang, T. Lei, Z. Wang, C. Shen, D. Cao, and T. Hou. ADMET evaluation in drug discovery. 20. prediction of breast cancer resistance protein inhibition through mach. learn. *J Cheminf.*, 12(1), 2020. doi: 10.1186/s13321-020-00421-y.

Y. Kido, P. Matsson, and K. M. Giacomini. Profiling of a prescription drug library for potential renal drug–drug interactions mediated by the organic cation transporter 2. *J. Med. Chem.*, 54(13): 4548–4558, 2011. doi: 10.1021/jm2001629.

N. C. Kleinstreuer, A. L. Karmaus, K. Mansouri, D. G. Allen, J. M. Fitzpatrick, and G. Patlewicz. Predictive models for acute oral systemic toxicity: A workshop to bridge the gap from research to regulation. *Computational Toxicology*, 8:21–24, 2018. doi: 10.1016/j.comtox.2018.08.002.

E. Kotsampasakou and G. F. Ecker. Predicting drug-induced cholestasis with the help of hepatic transporters—an in silico modeling approach. *J Chem. Inf. Model.*, 57(3):608–615, 2017. doi: 10.1021/acs.jcim.6b00518.

O. W. Lee, S. Austin, M. Gamma, D. M. Cheff, T. D. Lee, K. M. Wilson, J. Johnson, J. Travers, J. C. Braisted, R. Guha, C. Klumpp-Thomas, M. Shen, and M. D. Hall. Cytotoxic profiling of annotated and diverse chemical libraries using quantitative high-throughput screening. *SLAS DISCOVERY: Advancing the Science of Drug Discovery*, 25(1):9–20, 2019. doi: 10.1177/2472555219873068.

T. Lei, H. Sun, Y. Kang, F. Zhu, H. Liu, W. Zhou, Z. Wang, D. Li, Y. Li, and T. Hou. ADMET evaluation in drug discovery. 18. reliable prediction of chemical-induced urinary tract toxicity by boosting mach. learn. approaches. *Mol. Pharm.*, 14(11):3935–3953, 2017. doi: 10.1021/acs.molpharmaceut.7b00631.

M. Lindh, A. Karlén, and U. Norinder. Predicting the rate of skin penetration using an aggregated conformal prediction framework. *Mol. Pharm.*, 14(5):1571–1576, 2017. doi: 10.1021/acs.molpharmaceut.7b00007. URL `https://doi.org/10.1021/acs.molpharmaceut.7b00007`.

R. Liu, P. Schyman, and A. Wallqvist. Critically assessing the predictive power of QSAR models for human liver microsomal stability. *J Chem. Inf. Model.*, 55(8):1566–1575, 2015. doi: 10.1021/acs.jcim.5b00255.

F. Lombardo and Y. Jing. In silico prediction of volume of distribution in humans. extensive data set and the exploration of linear and nonlinear methods coupled with molecular interaction fields descriptors. *J Chem. Inf. Model.*, 56(10):2042–2052, 2016. doi: 10.1021/acs.jcim.6b00044.

K. Mansouri, N. F. Cariello, A. Korotcov, V. Tkachenko, C. M. Grulke, C. S. Sprankle, D. Allen, W. M. Casey, N. C. Kleinstreuer, and A. J. Williams. Open-source QSAR models for pKa prediction using multiple mach. learn. approaches. *J Cheminf.*, 11(1), 2019. doi: 10.1186/s13321-019-0384-1.

K. S. McLoughlin, C. G. Jeong, T. D. Sweitzer, A. J. Minnich, M. J. Tse, B. J. Bennion, J. E. Allen, S. Calad-Thomson, T. S. Rush, and J. M. Brase. Machine learning models to predict inhibition of the bile salt export pump. *J Chem. Inf. Model.*, 61(2):587–602, 2021. doi: 10.1021/acs.jcim.0c00950.

F. Montanari and G. F. Ecker. BCRP inhibition: from data collection to ligand-based modeling. *Molecular Informatics*, 33(5):322–331, 2014. doi: 10.1002/minf.201400012.

A. M. Orogo, S. S. Choi, B. L. Minnier, and N. L. Kruhlak. Construction and consensus performance of (q)SAR models for predicting phospholipidosis using a dataset of 743 compounds. *Molecular Informatics*, 31(10):725–739, 2012. doi: 10.1002/minf.201200048.

A. L. Perryman, D. Inoyama, J. S. Patel, S. Ekins, and J. S. Freundlich. Pruned machine learning models to predict aqueous solubility. *ACS Omega*, 5(27):16562–16567, July 2020. doi: 10.1021/acsomega.0c01251. URL `https://doi.org/10.1021/acsomega.0c01251`.

J. Rautio, J. E. Humphreys, L. O. Webster, A. Balakrishnan, J. P. Keogh, J. R. Kunta, C. J. Serabjit-Singh, and J. W. Polli. IN VITRO p-GLYCOPROTEIN INHIBITION ASSAYS FOR ASSESSMENT OF CLINICAL DRUG INTERACTION POTENTIAL OF NEW DRUG CANDIDATES: A RECOMMENDATION FOR PROBE SUBSTRATES. *Drug Metabolism and Disposition*, 34(5):786–792, 2006. doi: 10.1124/dmd.105.008615.

F. Schmidt, J. Wenzel, N. Halland, S. Güssregen, L. Delafoy, and A. Czich. Computational investigation of drug phototoxicity: Photosafety assessment, photo-toxophore identification, and mach. learn. *Chem. Res. Toxicol.*, 32(11):2338–2352, 2019. doi: 10.1021/acs.chemrestox.9b00338.

A. Serra, S. Önlü, P. Coretto, and D. Greco. An integrated quantitative structure and mechanism of action-activity relationship model of human serum albumin binding. *J Cheminf.*, 11(1), 2019. doi: 10.1186/s13321-019-0359-2.

B. Shaker, M.-S. Yu, J. S. Song, S. Ahn, J. Y. Ryu, K.-S. Oh, and D. Na. LightBBB: computational prediction model of blood–brain-barrier penetration based on LightGBM. *Bioinformatics*, 2020. doi: 10.1093/bioinformatics/btaa918. URL `https://doi.org/10.1093/bioinformatics/btaa918`.

S. Simeon, D. Montanari, and M. P. Gleeson. Investigation of factors affecting the performance of in silico volume distribution QSAR models for human, rat, mouse, dog & monkey. *Mol. Inf.*, 38(10):1900059, 2019. doi: 10.1002/minf.201900059. URL `https://doi.org/10.1002/minf.201900059`.

V. B. Siramshetty, D.-T. Nguyen, N. J. Martinez, N. T. Southall, A. Simeonov, and A. V. Zakharov. Critical assessment of artificial intelligence methods for prediction of hERG channel inhibition in the "big data" era. *J Chem. Inf. Model.*, 60(12):6007–6019, 2020. doi: 10.1021/acs.jcim.0c00884.

M. C. Sorkun, A. Khetan, and S. Er. AqSolDB, a curated reference set of aqueous solubility and 2d descriptors for a diverse set of compounds. *Sci. Data*, 6(1), 2019. doi: 10.1038/s41597-019-0151-1.

H. Sun, Y. Wang, D. M. Cheff, M. D. Hall, and M. Shen. Predictive models for estimating cytotoxicity on the basis of chemical structures. *Bioorganic & Medicinal Chemistry*, 28(10):115422, 2020. doi: 10.1016/j.bmc.2020.115422.

I. Sushko, S. Novotarskyi, R. Körner, A. K. Pandey, M. Rupp, W. Teetz, S. Brandmaier, A. Abdelaziz, V. V. Prokopenko, V. Y. Tanchuk, R. Todeschini, A. Varnek, G. Marcou, P. Ertl, V. Potemkin, M. Grishina, J. Gasteiger, C. Schwab, I. I. Baskin, V. A. Palyulin, E. V. Radchenko, W. J. Welsh, V. Kholodovych, D. Chekmarev, A. Cherkasov, J. A. de Sousa, Q.-Y. Zhang, A. Bender, F. Nigsch, L. Patiny, A. Williams, V. Tkachenko, and I. V. Tetko. Online chemical modeling environment (OCHEM): web platform for data storage, model development and publishing of chemical information. *J Comp-Aided Mol. Des.*, 25(6):533–554, 2011. doi: 10.1007/s10822-011-9440-2.

I. V. Tetko, S. Novotarskyi, I. Sushko, V. Ivanov, A. E. Petrenko, R. Dieden, F. Lebon, and B. Mathieu. Development of dimethyl sulfoxide solubility models using 163 000 molecules: Using a domain applicability metric to select more reliable predictions. *J Chem. Inf. Model.*, 53(8):1990–2000, 2013. doi: 10.1021/ci400213d.

A. Türková, S. Jain, and B. Zdrazil. Integrative data mining, scaffold analysis, and sequential binary classification models for exploring ligand profiles of hepatic organic anion transporting polypeptides. *J Chem. Inf. Model.*, 59(5):1811–1825, 2018. doi: 10.1021/acs.jcim.8b00466.

M. V. S. Varma, R. S. Obach, C. Rotter, H. R. Miller, G. Chang, S. J. Steyn, A. El-Kattan, and M. D. Troutman. Physicochemical space for optimum oral bioavailability: Contribution of human intestinal absorption and first-pass elimination. *Journal of Medicinal Chemistry*, 53(3):1098–1108, 2010. doi: 10.1021/jm901371v.

H. Veith, N. Southall, R. Huang, T. James, D. Fayne, N. Artemenko, M. Shen, J. Inglese, C. P. Austin, D. G. Lloyd, and D. S. Auld. Comprehensive characterization of cytochrome p450 isozyme selectivity across chemical libraries. *Nat. Biotechnol.*, 27(11):1050–1055, 2009. doi: 10.1038/nbt.1581.

N.-N. Wang, C. Huang, J. Dong, Z.-J. Yao, M.-F. Zhu, Z.-K. Deng, B. Lv, A.-P. Lu, A. F. Chen, and D.-S. Cao. Predicting human intestinal absorption with modified random forest approach: a comprehensive evaluation of molecular representation, unbalanced data, and applicability domain issues. *RSC Adv.*, 7(31):19007–19018, 2017. doi: 10.1039/c6ra28442f.

P.-H. Wang, Y.-S. Tu, and Y. J. Tseng. PgpRules: a decision tree based prediction server for p-glycoprotein substrates and inhibitors. *Bioinformatics*, 35(20):4193–4195, 2019. doi: 10.1093/bioinformatics/btz213.

R. Watanabe, T. Esaki, H. Kawashima, Y. Natsume-Kitatani, C. Nagao, R. Ohashi, and K. Mizuguchi. Predicting fraction unbound in human plasma from chemical structure: Improved accuracy in the low value ranges. *Mol. Pharm.*, 15(11):5302–5311, 2018. doi: 10.1021/acs.molpharmaceut.8b00785.

J. Wenzel, H. Matter, and F. Schmidt. Predictive multitask deep neural network models for ADME-tox properties: Learning from large data sets. *Journal of Chemical Information and Modeling*, 59 (3):1253–1268, 2019. doi: 10.1021/acs.jcim.8b00785.

M. B. Wittwer, A. A. Zur, N. Khuri, Y. Kido, A. Kosaka, X. Zhang, K. M. Morrissey, A. Sali, Y. Huang, and K. M. Giacomini. Discovery of potent, selective multidrug and toxin extrusion transporter 1 (MATE1, SLC47a1) inhibitors through prescription drug profiling and computational modeling. *J. Med. Chem.*, 56(3):781–795, 2013. doi: 10.1021/jm301302s.

C. Xu, F. Cheng, L. Chen, Z. Du, W. Li, G. Liu, P. W. Lee, and Y. Tang. In silico prediction of chemical ames mutagenicity. *J Chem. Inf. Model.*, 52(11):2840–2847, 2012. doi: 10.1021/ci300400a.

Y. Yuan, S. Chang, Z. Zhang, Z. Li, S. Li, P. Xie, W.-P. Yau, H. Lin, W. Cai, Y. Zhang, and X. Xiang. A novel strategy for prediction of human plasma protein binding using mach. learn. techniques. *Chemom. Intell. Lab. Syst.*, 199:103962, 2020. doi: 10.1016/j.chemolab.2020.103962.

Y. E. Yun, R. Tornero-Velez, S. T. Purucker, D. T. Chang, and A. N. Edginton. Evaluation of quantitative structure property relationship algorithms for predicting plasma protein binding in humans. *Computational Toxicology*, 17:100142, Feb. 2021. doi: 10.1016/j.comtox.2020.100142. URL `https://doi.org/10.1016/j.comtox.2020.100142`.

H. Zhang, P. Yu, T.-G. Zhang, Y.-L. Kang, X. Zhao, Y.-Y. Li, J.-H. He, and J. Zhang. In silico prediction of drug-induced myelotoxicity by using naïve bayes method. *Mol. Divers*, 19(4):945–953, 2015. doi: 10.1007/s11030-015-9613-3.

H. Zhang, J.-X. Ma, C.-T. Liu, J.-X. Ren, and L. Ding. Development and evaluation of in silico prediction model for drug-induced respiratory toxicity by using naïve bayes classifier method. *Food Chem. Toxicol.*, 121:593–603, 2018. doi: 10.1016/j.fct.2018.09.051.

H. Zhang, C.-T. Liu, J. Mao, C. Shen, R.-L. Xie, and B. Mu. Development of novel in silico prediction model for drug-induced ototoxicity by using naïve bayes classifier approach. *Toxicol. In Vitro*, 65: 104812, 2020. doi: 10.1016/j.tiv.2020.104812.

L. Zhang, H. Ai, W. Chen, Z. Yin, H. Hu, J. Zhu, J. Zhao, Q. Zhao, and H. Liu. CarcinoPred-EL: Novel models for predicting the carcinogenicity of chemicals using molecular fingerprints and ensemble learning methods. *Sci. Rep.*, 7(1), 2017. doi: 10.1038/s41598-017-02365-0.

X. Zhang, P. Zhao, Z. Wang, X. Xu, G. Liu, Y. Tang, and W. Li. In silico prediction of CYP2c8 inhibition with machine-learning methods. *Chem. Res. Toxicol*, 34(8):1850–1859, 2021. doi: 10.1021/acs.chemrestox.1c00078.

S. Zheng, W. Chang, W. Liu, G. Liang, Y. Xu, and F. Lin. Computational prediction of a new ADMET endpoint for small molecules: Anticommensal effect on human gut microbiota. *J Chem. Inf. Model.*, 59(3):1215–1220, 2018. doi: 10.1021/acs.jcim.8b00600.

S. Zheng, Y. Wang, W. Liu, W. Chang, G. Liang, Y. Xu, and F. Lin. In silico prediction of hemolytic toxicity on the human erythrocytes for small molecules by machine-learning and genetic algorithm. *J. Med. Chem.*, 63(12):6499–6512, 2019. doi: 10.1021/acs.jmedchem.9b00853.

S. Zheng, J. Xiong, Y. Wang, G. Liang, Y. Xu, and F. Lin. Quantitative prediction of hemolytic toxicity for small molecules and their potential hemolytic fragments by mach. learn. and recursive fragmentation methods. *J Chem. Inf. Model.*, 60(6):3231–3245, 2020. doi: 10.1021/acs.jcim.0c00102.
